# Supplementary material for: Seroprevalence of antibodies to SARS-CoV-2 in healthcare workers: a cross-sectional study
Source: BMJ Open. 2021 Feb 12;11(2):e043584. doi: 10.1136/bmjopen-2020-043584 (PMC7883610; doi:10.1136/bmjopen-2020-043584)
Supplement: Supplementary data [file bmjopen-2020-043584supp001.pdf]

SUPPLEMENTAL MATERIAL

Seroprevalence of Antibodies to SARS-CoV-2 in Healthcare Workers:

A Cross-Sectional Study

Ebinger JE, *et al. BMJ Open* 2021; 11:e043584. doi: 10.1136/bmjopen-2020-043584

Supplemental Table 1. Prior Studies Reporting Sensitivity for the Abbott Architect SARS-CoV-2 IgG Assay[1-14]

Author	Positive Tests	Total Tests	Sensitivity %	Sample Description
Abbott[1]	109	115	94.78%	Using data from >=8 days post symptom onset and including 5 immunocompromised samples. Positive subjects who tested positive for SARS-CoV-2 by a polymerase chain reaction (PCR) method and who also presented with Covid-19 symptoms.
Bryan and Pepper et al.[2]	668	689	96.95%	Serum specimens sent for clinical testing from persons who tested RT-PCR positive for SARS-CoV -2 during March and April 2020.
Ng and Goldgof and Shy and Levine and Balcerek and Bapat et al.[15]	328	382	85.86%	Received care at adult inpatient units or clinics and were RT PCR positive for SARS-CoV-2 from nasopharyngeal and/or oropharyngeal swab testing. Using combined data from immunocompromised individuals. Combining data from Day 8 + PSO.
Ekelund et al.[4]	17	20	85.00%	Serum samples from 16 individuals that prior to serum sampling had tested RT-PCR positive for SARS-CoV-2 in nasopharyngeal and/or pharyngeal swabs. The interval between onset of Covid-19 symptoms to serum sample collection ranged from 18 to 52 days (median 38 days).
Phipps and SoRelle et al.[5]	10	21	47.62%	8 or more days PSO. suspected Covid-19 cases with PCR-based nasopharyngeal swab testing on the m2000 Abbott RealTime SARS Cov-2 assay or the Abbott ID NOWTM Covid-19 assay.
Phipps and SoRelle et al.[5]	10	13	76.92%	Indeterminate days from PSO. Suspected Covid-19 cases with PCR-based nasopharyngeal swab testing on the m2000 Abbott RealTime SARS Cov-2 assay or the Abbott ID NOWTM Covid-19 assay.
Chew et al.[6]	65	96	67.71%	Used COVID pts at different stage of disease: results based on 7 + PSO disease stage: ≤6 days (7/81), at 7–13 days (17/39), at 14–20 days (21/25), and at ≥21 days (27/32)
Theel et al.[7]	78	84	92.86%	Anti-SARS-CoV-2 IgG assay sensitivity in convalescent sera and in individual patients tested ≥15 days post-symptom onset or first positive SARS-CoV-2 RT-PCR result
Theel et al.[7]	123	175	70.29%	Included inpatients and outpatients PCR positive from >= 8 PSO
Kohmer et al.[8]	35	45	77.78%	From 45 pts with positive PCR
Stroemer et al.[9]	33	34	97.06%	34 sera obtained from 26 patients between four and 60 days (median 19 days) after a positive real-time RT-PCR.
Nicol et al.[10]	115	141	81.56%	141 serum from 82 patients with positive PCR varying days from PSO
Dellière et al.[11]	86	95	90.53%	Serum samples (n=95) from patients at least 10 days from symptoms onset or positive PCR
Perkmann et al. [12]	55	65	84.62%	65 Covid-19 donors/patients with a symptom onset to analysis time of ≥14 days
Mueller et al.[13]	7	8	87.50%	8 RT-PCR positive individuals
Tang et al.[14]	56	71	78.87%	103 specimens from 48 patients with PCR confirmed SARS-CoV-2 infections from NP, OP or lower respiratory swab. Reported positive results from time from PCR: 0d=12/27, 1-3d= 8/15, 3-7d=13/22, 8-13d=16/23, >14d=13/16. and reported positive from symptoms onset: <3d= 0/12, 3-7d=6/20, 8-13=11/23, >14d=45/48
Cedars-Sinai Department of Pathology and Laboratory Medicine*	53	60	88.33%	All COVID Positive subjects were selected by three criteria: (1) Presentation to Cedars-Sinai Medical Center with symptoms consistent with infection by SARS-CoV-2 virus; (2) Were PCR positive for SARS-CoV-2 viral RNA in at least one nasopharyngeal sample; (3) Had EDTA or heparin plasma available for testing which was collected 8 or more days after onset of symptoms according to physician's notes in the medical record.

*Unpublished data

Ebinger JE, *et al. BMJ Open* 2021; 11:e043584. doi: 10.1136/bmjopen-2020-043584

Supplemental Table 2. Prior Studies Reporting Specificity for the Abbott Architect SARS-CoV-2 IgG Assay

Author	Negative Test	Total Tests	Specificity %	Sample source
Abbott[1]	1066	1070	99.63%	997 specimens were collected prior to September 2019<br>73 specimens were collected in 2020 with signs of respiratory illness and Covid-19 RT-PCR negative
Bryan and Pepper et al.[2]	1019	1020	99.90%	Serum samples from 2018 and 2019
Jääskeläinen et al.[16]	79	81	97.53%	Serum samples from 2018 and 2019
Ng, Goldgof, Shy, Levine, Balcerek and Bapat et al.[15]	1011	1013	99.80%	US blood donors prior to the Covid-19 pandemic
	234	235	99.57%	Plasma samples from 163 Covid-19 RT-PCR negative
Ekelund et al.[4]	100	100	100%	Pre-pandemic samples from 2018
Phipps and SoRelle et al.[5]	656	656	100%	240 samples collected prior to the Covid-19 pandemic (blood donors September through November 2019), and an additional 416 healthy donors without recent illness collected from March to April, 2020
	91	91	100%	23 CMV IgG positive, 8 prior Flu A+, 7 Flu B+, 6 RSV+, 47 endemic coronavirus samples (January 1, 2015- September 30, 2019) with normal or high levels of total IgG with no infusion of intravenous immunoglobulin in the preceding 3 months
	29	29	100%	Lupus patients that were positive for multiple autoantibodies (100% ANA, 62% anti-dsDNA, 75% anti-U1RNP, 55% anti-Sm, 34% anti-Ro52, 170 and 24% anti-La) 2004-2007
	20	20	100%	Rheumatoid arthritis patients positive for rheumatoid factor (85% were also anti-CCP positive) 2011-2014
	96	97	98.97%	Patients with Covid-19 RT-PCR negative
Chew et al.[6]	163	163	100%	
Theel et al.[7]	149	149	100%	Healthy samples from 2018
	104	105	99.05%	Samples negative for Covid-19 but positive for antibodies from other respiratory virus or bacteria (2020)
Kohmer et al.[8]	35	35	100%	
Ströemer et al.[9]	99	100	99.00%	100 archived samples from winter and summer seasons
Nicol et al.[10]	57	57	100%	52 patients with symptoms of Covid-19 but negative RT-PCR
	49	50	98.00%	Residual serum samples collected before Covid-19 in Mar 2019
	25	25	100%	Samples with potential cross-reaction to Covid-19
	10	10	100%	Samples from pregnant women
	10	10	100%	Samples with positive rheumatoid factor

Ebinger JE, *et al. BMJ Open* 2021; 11:e043584. doi: 10.1136/bmjopen-2020-043584

Paiva et al.[17]	1055	1059	99.62%	Combining random Covid-19 samples during March 2020 (negative RT-PCR), pre-pandemic samples, and pre pandemic prenatal samples. False positive tests (4) were from samples with Hepatitis A, Hepatitis B, Rheumatoid Factor and anti-DNA
Brecher et al.[18]	20	20	100%	Patients with PCR Documented Common Cold
Dellière et al.[11]	42	42	100%	42 patients from pre-pandemic. 14 healthy, 16 endemic corona virus, 1 rhino virus, 1 metapneumovirus, 1 influenza A, 1 RSV. 1 HIV, 1 Hepatitis B. 1 toxoplasmosis. 2 Rheumatoid Factor
Perkmann et al.[12]	490	494	99.19%	Cross selection of Viennese population, LEAD study between November and April to enrich seasonal infections
	299	302	99.01%	Healthy voluntary donors
	356	358	99.44%	Patients with rheumatic disease
Mueller et al.[13]	26	26	100%	Patients with suspected Covid but negative neutralization test and PCR
Tang et al.[14]	152	153	99.35%	80 patients symptomatic for Covid-19 but negative RT-PCR. 50 samples collected in 2015. 5 samples with other corona virus infection. 4 samples with Influenza A or B. 14 samples with interfering antibiotics.
Cedars-Sinai Department of Pathology and Laboratory Medicine*	178	178	100%	Samples collected prior to 1/1/2020

Supplemental Table 3. Prevalence of Measurable SARS-CoV-2 IgG Antibody in the Study Sample

	Mean (95% CI)
**Overall**	**4.1 (3.1, 5.7)**
Sex: Female	3.9 (3.0, 5.6)
Sex: Male	4.3 (3.1, 6.3)
Age: <25	4.5 (2.4, 7.7)
Age: 25-29	5.1 (3.4, 7.7)
Age: 30-34	5.1 (3.5, 7.5)
Age: 35-39	3.6 (2.3, 5.3)
Age: 40-44	4 (2.6, 6.1)
Age: 45-49	3.2 (1.8, 5.1)
Age: 50-54	3.7 (2.1, 5.7)
Age: 55-59	3.5 (1.9, 5.6)
Age: 60-64	3.8 (2.2, 6.0)
Age: >65	3.1 (1.5, 5.1)
Race Eth.: Asian	3.4 (2.4, 5.0)
Race Eth.: Black	4.8 (2.8, 8.0)
Race Eth.: Hispanic / Latino	5.7 (3.9, 8.3)
Race Eth.: Other	3.4 (1.8, 5.4)
Race Eth.: White	3.1 (2.1, 4.5)

Ebinger JE, *et al. BMJ Open* 2021; 11:e043584. doi: 10.1136/bmjopen-2020-043584

Supplemental Table 4. Pre-Existing Factors Associated with SARS-CoV-2 Seroprevalence

Predictors	Outcome: Antibody Positive N=6,062 (everybody with a test result)				Outcome: IgG index (divided by 10) N=212 (everybody with a test result)			
	Model 1		Model 2		Model 3		Model 4	
	OR (95% CI)	P	OR (95% CI)	P	Est (SE)	P	Est (SE)	P
Age (per decade)	0.80 (0.70, 0.91)	0.001	0.81 (0.71, 0.92)	0.001	0.02 (0.01)	0.07		
Male Sex	1.19 (0.89, 1.59)	0.24			-0.05 (0.03)	0.11		
Hispanic Ethnicity	1.76 (1.29, 2.40)	<0.001	1.80 (1.31, 2.46)	<0.001	0.00 (0.03)	0.93		
African American Race	1.77 (1.07, 2.93)	0.027	1.72 (1.03, 2.89)	0.04	0.02 (0.05)	0.66		
Smoking	0.83 (0.26, 2.66)	0.76			-0.01 (0.11)	0.91		
Vaping	1.12 (0.40, 3.12)	0.82			-0.08 (0.10)	0.45		
Asthma	0.48 (0.28, 0.83)	0.009	0.48 (0.28, 0.83)	0.009	0.02 (0.05)	0.71		
Autoimmune disease	0.50 (0.18, 1.35)	0.17			-0.07 (0.10)	0.49		
Cancer	0.54 (0.17, 1.72)	0.29			0.01 (0.12)	0.92		
Cardiovascular Disease	0.49 (0.12, 2.02)	0.33			0.06 (0.14)	0.65		
Chronic Obstructive Pulmonary Disease	0.00 (0.00, inf)	0.97						
Diabetes Mellitus	0.66 (0.32, 1.37)	0.26			0.07 (0.07)	0.31		
Hypertension	0.90 (0.58, 1.41)	0.64			0.11 (0.04)	0.013	0.12 (0.04)	0.003
Obesity	0.82 (0.55, 1.24)	0.35			0.01 (0.04)	0.71		

Logistic model 1 is adjusted for age, sex, ethnicity, race.
Logistic model 2 is adjusted for anything that was significant in Model 1 to a P<0.05.

Linear model 3 is adjusted for age, sex
Linear model 4 is adjusted for anything that was significant in Model 3 to a P<0.05.

Supplemental Table 5. Potential COVID Illness Exposure Related Factors Associated with SARS-CoV-2 Seroprevalence

	Outcome: Antibody Positive N=6,062 (everybody with a test result)				Outcome: IgG index (divided by 10) N=212 (everybody with a test result)			
	Model 1		Model 2		Model 3		Model 4	
**Predictors**	**OR (95% CI)**	**P**	**OR (95% CI)**	**P**	**Est (SE)**	**P**	**Est (SE)**	**P**
Age (per decade)	0.80 (0.70, 0.91)	0.001	0.84 (0.73, 0.97)	0.016	0.02 (0.01)	0.07		
Male Sex	1.19 (0.89, 1.59)	0.24			-0.05 (0.03)	0.11		
Hispanic Ethnicity	1.76 (1.28, 2.4)	<0.001	1.84 (1.31, 2.59)	0.001	0.00 (0.03)	0.93		
African American Race	1.77 (1.07, 2.93)	0.027	2.11 (1.24, 3.58)	0.006	0.02 (0.05)	0.66		
# people in home	1.02 (0.94, 1.11)	0.6			0.02 (0.01)	0.038	0.01 (0.01)	0.13
Physician Suspected Covid Diagnosis	10.14 (7.59, 13.55)	<0.001	7.78 (5.73, 10.56)	<0.001	0.16 (0.02)	<0.001	0.13 (0.03)	<0.001
Household Covid Diagnosis	18.93 (11.74, 30.53)	<0.001	9.42 (5.5, 16.13)	<0.001	0.09 (0.04)	0.016	0.02 (0.04)	0.55
Domestic Travel	0.61 (0.44, 0.84)	0.002	0.67 (0.48, 0.94)	0.021	-0.05 (0.03)	0.08		
International Travel	0.93 (0.66, 1.31)	0.68			0.00 (0.03)	0.98		
Covid Unit	1.98 (1.49, 2.63)	<0.001	1.61 (1.18, 2.18)	0.002	0.10 (0.03)	<0.001	0.06 (0.03)	0.026
Dwelling: House	1.20 (0.89, 1.61)	0.23			0.03 (0.03)	0.27		
Dwelling: Other	1.17 (0.58, 2.35)	0.67			0.05 (0.07)	0.44		
Persons <18 in home	0.96 (0.71, 1.29)	0.77			0.03 (0.03)	0.31		
Person <12 in home	0.91 (0.66, 1.26)	0.58			0.02 (0.03)	0.47		
Cats in home	0.98 (0.65, 1.48)	0.92			-0.01 (0.04)	0.87		
Dogs in home	1.34 (1.02, 1.78)	0.039	1.29 (0.95, 1.75)	0.10	0.01 (0.03)	0.78		

Logistic model 1 is adjusted for age, sex, race, ethnicity.
Logistic model 2 is adjusted for anything that was significant in Model 1 to a P<0.05.

Linear model 3 is adjusted age, sex
Linear model 4 is adjusted for anything that was significant in Model 3 to a P<0.05.

Ebinger JE, *et al. BMJ Open* 2021; 11:e043584. doi: 10.1136/bmjopen-2020-043584

Supplemental Table 6. Potential COVID Illness Response Factors Associated with SARS-CoV-2 Seroprevalence

	Outcome: Antibody Positive N=6,062 (everybody with a test result)				Outcome: IgG index (divided by 10) N=212 (everybody with a test result)			
	Model 1		Model 2		Model 3		Model 4	
Predictors	OR (95% CI)	P	OR (95% CI)	P	Est (SE)	P	Est (SE)	P
Age (per decade)	0.8 (0.7, 0.91)	0.001	0.77 (0.66, 0.91)	0.002	0.02 (0.01)	0.07		
Male Sex	1.19 (0.89, 1.59)	0.24			-0.05 (0.03)	0.11		
Hispanic Ethnicity	1.76 (1.29, 2.4)	<0.001	1.93 (1.31, 2.84)	0.001	0 (0.03)	0.93		
African American Race	1.77 (1.07, 2.93)	0.027	1.72 (0.91, 3.26)	0.09	0.02 (0.05)	0.66		
Fever	7.8 (5.81, 10.48)	<0.001	2.2 (1.31, 3.69)	0.003	0.15 (0.03)	<0.001	0.08 (0.04)	0.032
Chills	6.23 (4.67, 8.31)	<0.001	1.28 (0.75, 2.18)	0.36	0.11 (0.03)	<0.001	-0.04 (0.04)	0.31
Headache	2.72 (2.03, 3.64)	<0.001	0.67 (0.43, 1.06)	0.09	0.12 (0.03)	<0.001	0.06 (0.04)	0.11
Conjunctivitis	2.56 (1.45, 4.52)	0.001	0.89 (0.42, 1.86)	0.75	-0.04 (0.06)	0.5		
Anosmia	23.05 (16.98, 31.29)	<0.001	11.91 (7.77, 18.24)	<0.001	0.08 (0.03)	0.002	-0.01 (0.03)	0.81
Nasal Congestion	2.59 (1.95, 3.44)	<0.001	1.22 (0.73, 2.04)	0.44	0.07 (0.03)	0.017	0.01 (0.03)	0.83
Rhinorrhea	1.89 (1.41, 2.52)	<0.001	0.59 (0.36, 0.97)	0.039	0.02 (0.03)	0.41		
Dry Cough	4.28 (3.21, 5.69)	<0.001	1.82 (1.18, 2.81)	0.007	0.09 (0.03)	0.001	-0.04 (0.04)	0.25
Productive Cough	3.01 (2.16, 4.2)	<0.001	0.83 (0.5, 1.37)	0.46	0.09 (0.03)	0.005	0.01 (0.04)	0.73
Sore Throat	2.09 (1.56, 2.8)	<0.001	0.48 (0.31, 0.75)	0.001	0.03 (0.03)	0.3		
Chest Pain	3.2 (2.26, 4.53)	<0.001	0.96 (0.56, 1.63)	0.88	0.07 (0.03)	0.034	-0.05 (0.04)	0.24
Dyspnea	4.08 (3, 5.56)	<0.001	0.88 (0.54, 1.43)	0.6	0.16 (0.03)	<0.001	0.13 (0.04)	0.001
Anorexia	8.57 (6.31, 11.63)	<0.001	2.19 (1.34, 3.57)	0.002	0.14 (0.03)	<0.001	0.06 (0.04)	0.13
Nausea	2.59 (1.86, 3.6)	<0.001	0.88 (0.52, 1.47)	0.62	0.1 (0.03)	0.002	0.08 (0.04)	0.049
Vomiting	2.33 (1.34, 4.03)	0.003	0.67 (0.3, 1.47)	0.31	0.15 (0.05)	0.005	-0.06 (0.06)	0.35
Diarrhea	2.32 (1.69, 3.18)	<0.001	0.82 (0.52, 1.29)	0.39	0.08 (0.03)	0.014	-0.05 (0.04)	0.22
Myalgias	6.36 (4.76, 8.5)	<0.001	1.88 (1.11, 3.17)	0.019	0.13 (0.03)	<0.001	0.04 (0.04)	0.35
Fatigue	5.91 (4.38, 7.98)	<0.001	1.58 (0.93, 2.69)	0.09	0.14 (0.03)	<0.001	0.02 (0.05)	0.67
Skin Changes	1.65 (0.96, 2.83)	0.07			0.01 (0.05)	0.88		
Stroke Symptoms	2.35 (0.71, 7.78)	0.16			0.27 (0.11)	0.019	0.05 (0.13)	0.73
Sneezing	1.72 (1.29, 2.28)	<0.001	0.82 (0.52, 1.31)	0.41	0.03 (0.03)	0.36		

Ebinger JE, *et al. BMJ Open* 2021; 11:e043584. doi: 10.1136/bmjopen-2020-043584

Logistic model 1 is adjusted for age, sex, race, ethnicity.
Logistic model 2 is adjusted for anything that was significant in Model 1 to a P<0.05.

Linear model 3 is adjusted for age, sex.
Linear model 4 is adjusted for anything that was significant in Model 3 to a P<0.05.

Ebinger JE, *et al. BMJ Open* 2021; 11:e043584. doi: 10.1136/bmjopen-2020-043584

Supplemental Table 7. Factors Associated with SARS-CoV-2

Predictors	Outcome: Antibody Positive N=6,062 (everybody with a test result)		Outcome: IgG index (divided by 10) N=212 (everybody with a test result)	
	OR (95% CI)	P	Est (SE)	P
Age (per decade)	0.80 (0.68, 0.94)	0.008		
Hispanic Ethnicity	1.98 (1.34, 2.92)	0.001		
African American Race	2.02 (1.08, 3.76)	0.027		
Asthma	0.25 (0.13, 0.51)	<0.001		
Hypertension			0.1 (0.04)	0.007
Physician Suspected Covid Diagnosis	3.85 (2.6, 5.69)	<0.001	0.1 (0.03)	0.001
Household Covid Diagnosis	5.73 (2.9, 11.32)	<0.001		
Domestic Travel	0.62 (0.42, 0.91)	0.015		
Covid Unit	1.76 (1.24, 2.5)	0.002	0.06 (0.03)	0.021
Fever	2.02 (1.28, 3.18)	0.002	0.03 (0.03)	0.26
Anosmia	11.04 (7.22, 16.88)	<0.001		
Rhinorrhea	0.58 (0.38, 0.88)	0.011		
Dry Cough	1.3 (0.84, 2)	0.23		
Sore Throat	0.53 (0.34, 0.82)	0.004		
Dyspnea			0.08 (0.03)	0.009
Anorexia	1.58 (0.98, 2.54)	0.06		
Nausea			0.06 (0.03)	0.05
Myalgias	1.65 (1.04, 2.63)	0.035		

Logistic and linear models are adjusted for significant predictors from the primary multivariable models examining associations of existing characteristics, exposures and symptoms with antibody positivity and IgG index.

Ebinger JE, et al. BMJ Open 2021; 11:e043584. doi: 10.1136/bmjopen-2020-043584

Supplemental Figure 1.

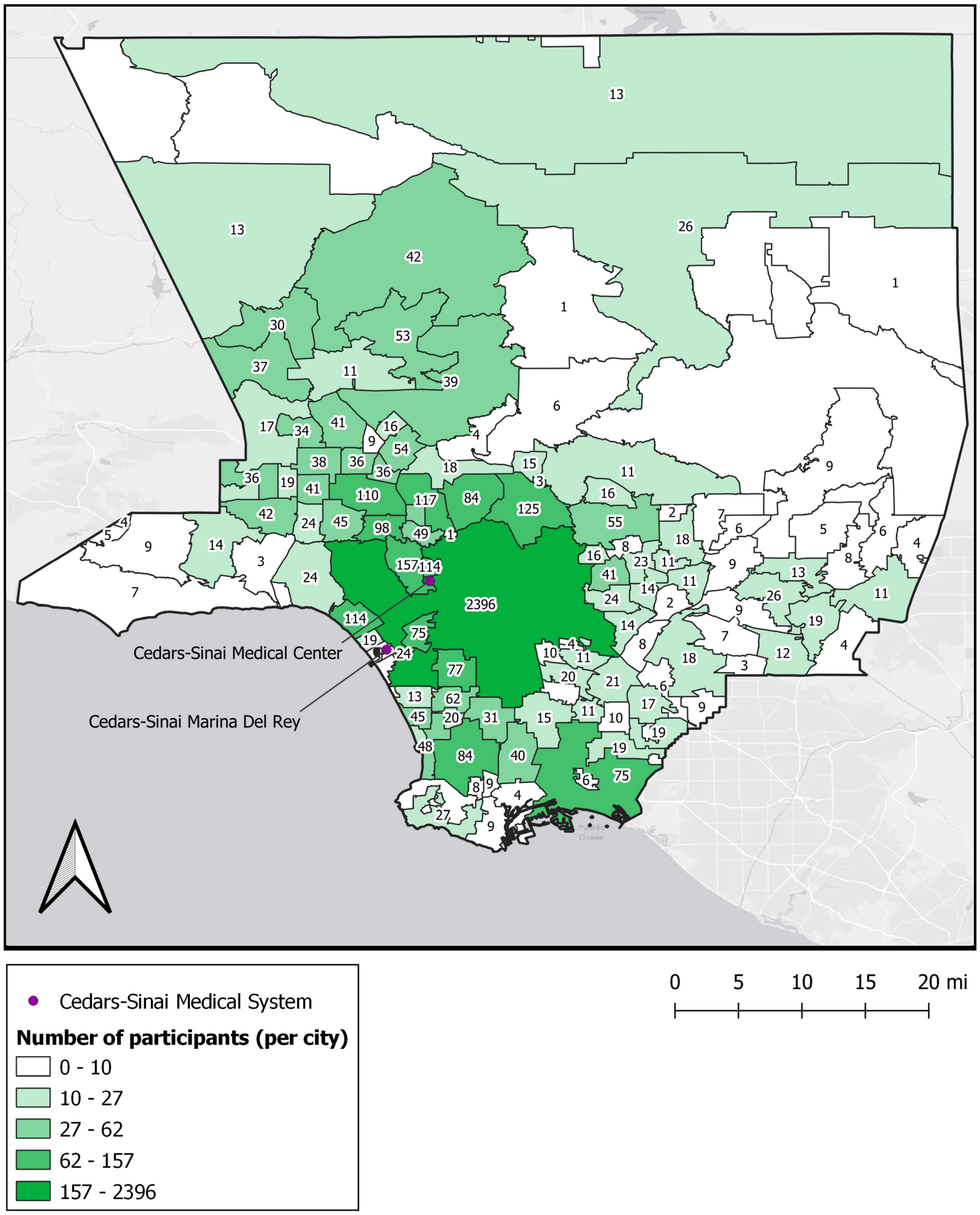

Ebinger JE, *et al. BMJ Open* 2021; 11:e043584. doi: 10.1136/bmjopen-2020-043584

SUPPLEMENTAL REFERENCES

1. Abbott. ARCHITECT SARS-CoV-2 IgG Instructions for Use. 2020.
2. Bryan A, Pepper G, Wener MH, et al. Performance Characteristics of the Abbott Architect SARS-CoV-2 IgG Assay and Seroprevalence in Boise, Idaho. *J Clin Microbiol.* 2020.
3. Ng D, Goldgof G, Shy B, et al. SARS-CoV-2 seroprevalence and neutralizing activity in donor and patient blood from the San Francisco Bay Area. *medRxiv.* 2020:2020.2005.2019.20107482.
4. Ekelund O, Ekblom K, Somajo S, Pattison-Granberg J, Olsson K, Petersson A. High-throughput immunoassays for SARS-CoV-2, considerable differences in performance when comparing three methods. *medRxiv.* 2020:2020.2005.2022.20106294.
5. Phipps WS, SoRelle JA, Li Q-Z, et al. SARS-CoV-2 Antibody responses do not predict COVID-19 disease severity. *medRxiv.* 2020:2020.2005.2015.20103580.
6. Chew KL, Tan SS, Saw S, et al. Clinical evaluation of serological IgG antibody response on the Abbott Architect for established SARS-CoV-2 infection. *Clinical Microbiology and Infection.* 2020.
7. Theel ES, Harring J, Hilgart H, Granger D. Performance Characteristics of Four High-Throughput Immunoassays for Detection of IgG Antibodies against SARS-CoV-2. *Journal of Clinical Microbiology.* 2020:JCM.01243-01220.
8. Kohmer N, Westhaus S, Rühl C, Ciesek S, Rabenau HF. Brief clinical evaluation of six high-throughput SARS-CoV-2 IgG antibody assays. *Journal of Clinical Virology.* 2020;129:104480.
9. Stroemer A, Grobe O, Rose R, Fickenscher H, Lorentz T, Krumbholz A. Diagnostic accuracy of six commercial SARS-CoV-2 IgG/total antibody assays and identification of SARS-CoV-2 neutralizing antibodies in convalescent sera. *medRxiv.* 2020:2020.2006.2015.20131672.

10. Nicol T, Lefeuvre C, Serri O, et al. Assessment of SARS-CoV-2 serological tests for the diagnosis of COVID-19 through the evaluation of three immunoassays: Two automated immunoassays (Euroimmun and Abbott) and one rapid lateral flow immunoassay (NG Biotech). *Journal of Clinical Virology.* 2020;129:104511.

11. Dellière S, Salmona M, Minier M, et al. Evaluation of COVID-19 IgG/IgM Rapid Test from Orient Gene Biotech. *Journal of Clinical Microbiology.* 2020:JCM.01233-01220.

12. Perkmann T, Perkmann-Nagele N, Breyer M-K, et al. Side by side comparison of three fully automated SARS-CoV-2 antibody assays with a focus on specificity. *medRxiv.* 2020:2020.2006.2004.20117911.

13. Mueller L, Ostermann PN, Walker A, et al. Sensitivity of commercial Anti-SARS-CoV-2 serological assays in a high-prevalence setting. *medRxiv.* 2020:2020.2006.2011.20128686.

14. Tang MS, Hock KG, Logsdon NM, et al. Clinical Performance of Two SARS-CoV-2 Serologic Assays. *Clinical Chemistry.* 2020.

15. Guo W, Li M, Dong Y, et al. Diabetes is a risk factor for the progression and prognosis of COVID-19. *Diabetes/metabolism research and reviews.* 2020:e3319.

16. Jääskeläinen AJ, Kuivanen S, Kekäläinen E, et al. Performance of six SARS-CoV-2 immunoassays in comparison with microneutralisation. *medRxiv.* 2020:2020.2005.2018.20101618.

17. Paiva KJ, Grisson RD, Chan PA, et al. Validation and Performance Comparison of Three SARS-CoV-2 Antibody Assays. *bioRxiv.* 2020:2020.2005.2029.124776.

18. Brecher SM, Dryjowicz-Burek J, Yu H, Campbell S, Ratcliffe N, Gupta K. Patients with Common Cold Coronaviruses Tested Negative for IgG Antibody to SARS-CoV-2. *Journal of Clinical Microbiology.* 2020:JCM.01029-01020.

Ebinger JE, *et al. BMJ Open* 2021; 11:e043584. doi: 10.1136/bmjopen-2020-043584
