# Supplementary material for: Seroprevalence of antibodies to SARS-CoV-2 in healthcare workers: a cross-sectional study
Source: BMJ Open. 2021 Feb 12;11(2):e043584. doi: 10.1136/bmjopen-2020-043584 (PMC7883610; doi:10.1136/bmjopen-2020-043584)
Supplement: Author's manuscript [file bmjopen-2020-043584.draft_revisions.pdf]

BMJ Open is committed to open peer review. As part of this commitment we make the peer review history of every article we publish publicly available.

When an article is published we post the peer reviewers' comments and the authors' responses online. We also post the versions of the paper that were used during peer review. These are the versions that the peer review comments apply to.

The versions of the paper that follow are the versions that were submitted during the peer review process. They are not the versions of record or the final published versions. They should not be cited or distributed as the published version of this manuscript.

BMJ Open is an open access journal and the full, final, typeset and author-corrected version of record of the manuscript is available on our site with no access controls, subscription charges or pay-per-view fees (http://bmjopen.bmj.com).

If you have any questions on BMJ Open's open peer review process please email info.bmjopen@bmj.com

BMJ Open

SARS-CoV-2 Seroprevalence Across a Diverse Cohort of Healthcare Workers

Journal:	*BMJ Open*
Manuscript ID	bmjopen-2020-043584
Article Type:	Original research
Date Submitted by the Author:	10-Aug-2020
Complete List of Authors:	Ebinger, Joseph ; Cedars-Sinai Medical Center<br>Botwin, Gregory ; Cedars-Sinai Medical Center<br>Albert, Christine; Cedars-Sinai Medical Center<br>Alotaibi, Mona; University of California San Diego, Department of Pulmonology<br>Arditi, Moshe; Cedars-Sinai Medical Center, Division of Pediatric Infectious Diseases<br>Berg, Anders; Cedars-Sinai Medical Center<br>Binek , Aleksandra; Cedars-Sinai Medical Center<br>Botting, Patrick; Cedars-Sinai Medical Center<br>Fert-Bober, Justyna; Cedars-Sinai Medical Center<br>Figueiredo, Jane; Cedars-Sinai Medical Center<br>Grein, Jonathan; Cedars-Sinai Medical Center<br>Hasan, Wohaib; Cedars-Sinai Medical Center<br>Henglin, Mir; Cedars-Sinai Medical Center<br>Hussain, Shehnaz; Cedars-Sinai Medical Center<br>Jain, Mohit; University of California San Diego, Department of Medicine and Pharmacology<br>Joung, Sandy; Cedars-Sinai Medical Center<br>Karin, Michael; University of California San Diego School of Medicine<br>Kim, Elizabeth; Cedars-Sinai Medical Center<br>Li, Dalin; Cedars-Sinai Medical Center<br>Liu, Yunxian; Cedars-Sinai Medical Center<br>Luong, Eric; Cedars-Sinai Medical Center<br>McGovern, Dermot; Cedars-Sinai Medical Center<br>Merchant, Akil; Cedars-Sinai Medical Center<br>Merin, Noah; Cedars-Sinai Medical Center<br>Miles, Peggy; Cedars-Sinai Medical Center<br>Minissian , Margo; Cedars-Sinai Medical Center<br>Nguyen , Trevor Trung; Cedars-Sinai Medical Center<br>Raedschelders , Koen; Cedars-Sinai Medical Center<br>Rashid, Mohamad; Cedars-Sinai Medical Center<br>Riera, Celine ; Cedars-Sinai Medical Center<br>Riggs, Richard; Cedars-Sinai Medical Center<br>Sharma , Sonia; La Jolla Institute for Allergy and Immunology<br>Sternbach, Sarah; Cedars-Sinai Medical Center<br>Sun, Nancy; Cedars-Sinai Medical Center<br>Tourtellotte, Warren; Cedars-Sinai Medical Center<br>Van Eyk, Jennifer; Cedars-Sinai Medical Center<br>Sobhani , Kimia; Cedars-Sinai Medical Center<br>Braun, Jonathan; Cedars-Sinai Medical Center

	Cheng, Susan; Cedars-Sinai Medical Center, Cardiology
Keywords:	COVID-19, INFECTIOUS DISEASES, Public health < INFECTIOUS DISEASES

SCHOLARONE™
Manuscripts

I, the Submitting Author has the right to grant and does grant on behalf of all authors of the Work (as defined in the below author licence), an exclusive licence and/or a non-exclusive licence for contributions from authors who are: i) UK Crown employees; ii) where BMJ has agreed a CC-BY licence shall apply, and/or iii) in accordance with the terms applicable for US Federal Government officers or employees acting as part of their official duties; on a worldwide, perpetual, irrevocable, royalty-free basis to BMJ Publishing Group Ltd ("BMJ") its licensees and where the relevant Journal is co-owned by BMJ to the co-owners of the Journal, to publish the Work in this journal and any other BMJ products and to exploit all rights, as set out in our licence.

The Submitting Author accepts and understands that any supply made under these terms is made by BMJ to the Submitting Author unless you are acting as an employee on behalf of your employer or a postgraduate student of an affiliated institution which is paying any applicable article publishing charge ("APC") for Open Access articles. Where the Submitting Author wishes to make the Work available on an Open Access basis (and intends to pay the relevant APC), the terms of reuse of such Open Access shall be governed by a Creative Commons licence – details of these licences and which Creative Commons licence will apply to this Work are set out in our licence referred to above.

Other than as permitted in any relevant BMJ Author's Self Archiving Policies, I confirm this Work has not been accepted for publication elsewhere, is not being considered for publication elsewhere and does not duplicate material already published. I confirm all authors consent to publication of this Work and authorise the granting of this licence.

For peer review only

SARS-CoV-2 Seroprevalence Across a Diverse Cohort of Healthcare Workers

Joseph E. Ebinger, MD, MS,[1,2]* Gregory J. Botwin, BS,[3]* Christine M. Albert, MD, MPH,[1,2] Mona Alotaibi,MD,[4] Moshe Arditi, MD,[2,5,6] Anders H. Berg, MD, PhD,[7] Aleksandra Binek, PhD,[8] Patrick Botting, MSPH,[1,2] Justyna Fert-Bober, Phd,[2] Jane C. Figueiredo, PhD[9] Jonathan D. Grein, MD,[10,11] Wohaib Hasan, PhD,[7,12] Mir Henglin, BA,[1,2] Shehnaz K. Hussain, PhD,[9] Mohit Jain, MD, PhD,[13] Sandy Joung, MHDS,[1,2] Michael Karin, PhD,[14] Elizabeth H. Kim, MHDS,[1,2] Dalin Li, PhD,[3] Yunxian Liu, PhD,[1,2] Eric Luong, MPH,[1,2] Dermot P.B. McGovern, MD, PhD,[3] Akil Merchant, MD,[10] Noah Merin, MD, PhD,[15] Peggy B. Miles, MD,[16] Margo Minissian, PhD,[1,2,17] Trevor-Trung Nguyen, BS,[1,2] Koen Raedschelders, PhD,[1,2,8] Mohamad A. Rashid, MBChB,[1,2] Celine E. Riera, PhD,[18,19] Richard V. Riggs, MD,[20] Sonia Sharma, PhD[21] Sarah Sternbach,BS,[2] Nancy Sun, MPS,[1,2] Warren G. Tourtellotte, MD, PhD,[7,12] Jennifer E. Van Eyk, PhD,[1,8,22] Kimia Sobhani, PhD,[7]* Jonathan G. Braun, MD, PhD,[7]* Susan Cheng, MD, MPH[1,2,22]*

From [1]Department of Cardiology, Cedars-Sinai Medical Center, Los Angeles, California, USA; [2]Smidt Heart Institute, Cedars-Sinai Medical Center, Los Angeles, California, USA; [3]F. Widjaja Foundation Inflammatory Bowel and Immunobiology Research Institute, Cedars-Sinai Medical Center, Los Angeles, California, USA; [4]Division of Pulmonary and Critical Care Medicine, University of California, San Diego, San Diego, California, USA; [5]Departments of Pediatrics, Division of Infectious Diseases and Immunology, and Infectious and Immunologic Diseases Research Center (IIDRC), Department of Biomedical Sciences, Cedars-Sinai Medical Center, Los Angeles, California, USA; [6]Department of Pediatrics, David Geffen School of Medicine at UCLA, Los Angeles, California, USA; [7]Department of Pathology and Laboratory Medicine, Cedars-Sinai Medical Center, Los Angeles, California, USA; [8]Advanced Clinical Biosystems Institute, Department of Biomedical Sciences, Cedars-Sinai Medical Center, Los Angeles, California, USA; [9]Cedars-Sinai Cancer and Department of Medicine, Cedars-Sinai Medical Center, Los Angeles, California, USA; [10]Department of Medicine, Cedars-Sinai Medical Center,

Los Angeles, California, USA; [11]Department of Epidemiology, Cedars-Sinai Medical Center, Los Angeles, California, USA; [12]Biobank & Translational Research Core Laboratory, Samuel Oschin Comprehensive Cancer Institute, Cedars-Sinai Medical Center, Los Angeles, California, USA; [13]Department of Medicine, School of Medicine, University of California, San Diego, San Diego, CA; [14]Department of Pharmacology, University of California, San Diego School of Medicine, San Diego, California, USA; [15]Department of Internal Medicine, Division of Hematology Cedars-Sinai Medical Center, Los Angeles, California, USA; [16]Employee Health Services, Department of Medicine, Cedars-Sinai Medical Center, Los Angeles, California, USA; [17]Brawerman Nursing Institute, Cedars-Sinai Medical Center, Los Angeles, California, USA; [18]Center for Neural Science and Medicine, Department of Biomedical Sciences, Board of Governors Regenerative Medicine Institute, Department of Neurology, Cedars-Sinai Medical Center, Los Angeles, California, USA; [19]David Geffen School of Medicine, University of California, Los Angeles, Los Angeles, California, USA; [20]Chief Medical Officer, Cedars-Sinai Medical Center, Los Angeles, California, USA; [21]La Jolla Institute for Allergy and Immunology, La Jolla, California,USA;[22]Barbra Streisand Women's Heart Center, Cedars-Sinai Medical Center, Los Angeles, California, USA.

Correspondence: Kimia Sobhani, PhD, Department of Pathology and Laboratory Medicine, Cedars-Sinai Medical Center, Los Angeles, CA; phone (310) 423-5405; email kimia.sobhani@cshs.org; Jonathan G. Braun, MD, PhD, F. Widjaja Foundation Inflammatory Bowel and Immunobiology Research Institute, Cedars Sinai Medical Center, Los Angeles, CA; phone (310) 423-8717; email jonathan.braun2@cshs.org; Susan Cheng, MD, MPH, Department of Cardiology, Smidt Heart Institute, Cedars Sinai Medical Center, Los Angeles, CA; phone (310) 423-2726; email susan.cheng@cshs.org.

Wordcount: 2,581

Key Words: Covid-19; Antibodies; Anosmia; Disparities; Healthcare workers

only

For peer review only - http://bmjopen.bmj.com/site/about/guidelines.xhtml

ABSTRACT

Objective: We sought to determine the extent of SARS-CoV-2 seroprevalance and the factors associated with seroprevelance across a diverse cohort of healthcare workers.

Design: Observational cohort study of healthcare workers, including SARS-CoV-2 serology testing and participant questionaires.

Settings: A multi-site healthcare delivery system located in Los Angeles County.

Participants: A diverse and unselected population of adults (n=6,062) employed in a multi-site healthcare delivery system located in Los Angeles County, including individuals with direct patient contact and others with non-patient-oriented work functions.

Main Outcomes: Using Bayesian and multi-variate analyses, we estimated seroprevalence and factors associated with seropositivity and antibody titers, including pre-existing demographic and clinical characteristics; potential Covid-19 illness related exposures; and, symptoms consistent with Covid-19 infection.

Results: We observed a seroprevalence rate of 4.1%, with anosmia as the most prominently associated self-reported symptom in addition to fever, dry cough, anorexia, and myalgias. After adjusting for potential confounders, pre-existing medical conditions were not associated with antibody positivity. However, seroprevalence was associated with younger age, Hispanic ethnicity, and African-American race, as well as presence of either a personal or household member having a prior diagnosis of Covid-19. Importantly, African American race and Hispanic ethnicity were associated with antibody positivity even after adjusting for personal Covid-19 diagnosis status, suggesting the contribution of unmeasured structural or societally factors. Notably, number of people, or children, in the home was not associated with antibody positivity.

Conclusion and Relevance: The demographic factors associated with SARS-CoV-2 seroprevalence among our healthcare workers underscore the importance of exposure sources

For peer review only - http://bmjopen.bmj.com/site/about/guidelines.xhtml

beyond the workplace. The size and diversity of our study population, combined with robust survey and modeling techniques, provide a vibrant picture of the demographic factors, exposures, and symptoms that can identify individuals with susceptibility as well as potential to mount an immune response to Covid-19.

STRENGTHS AND LIMITATIONS

- Our study is strengthened by the size and granularity of data available on participants
- The observational nature of the study precludes statements regarding causality.
- The broad definition of healthcare worker, including both patient facing and non-patient facing participants, enhances the generalizability of the results.
- The diverse participant population also enhances generalizability.

For peer review only

INTRODUCTION

Amidst the ongoing global pandemic caused by SARS-CoV-2, the viral agent causing Covid-19, substantial attention[1] turned to antibody testing as an approach to understanding patterns of exposure and immunity across populations. The use and interpretion of antibody testing to assess exposure and immunity remains frought with inconsistencies and unclear clinical correlations, in part due to a dearth of high quality studies among diverse participants.[2,3] Recent publications have pointed to the challenges and importance of understanding how different antibody tests for SARS-CoV-2 perform, and factors that may render one method superior to another.[4,5] Nonetheless, there remains general agreement that antibody testing offers valuable information regarding the probable extent of SARS-CoV-2 exposure, the factors associated with exposure, and the potential nature and determinants of seropositive status.[6]

To that end, we conducted a study of SARS-CoV-2 antibody screening of a large, diverse, and unselected population of adults employed in a multi-site healthcare delivery system located in Los Angeles County, including individuals with direct patient contact and others with non-patient-oriented work functions. Recognizing the range of factors that might influence antibody status in a given individual, we focused our study on not only estimating seroprevalence but also on identifying factors associated with seropositivity and relative antibody levels within the following three categories: (1) pre-existing demographic and clinical characteristics; (2) potential Covid-19 illness related exposures; and, (3) Covid-19 illness related response variables (i.e. different types of self-reported symptoms).

For peer review only - http://bmjopen.bmj.com/site/about/guidelines.xhtml

METHODS

Study Sample

The sampling strategy for our study has been described previously.[7] In brief, beginning on May 11, 2020, we enrolled a total of N=6,318 active employees working at multiple sites comprising the Cedars-Sinai Health System, located in the diverse metropolis of Los Angeles County, California. The Cedars-Sinai organization includes two hospitals (Cedars-Sinai Medical Center and Marina Del Rey Hospital) in addition to multiple clinics in the Cedars-Sinai Medical Delivery Network. All active employees (total N~15,000) were invited to participate in the study by providing a peripheral venous blood sample for serology testing and completing an electronic survey of questions regarding past medical history, social history, and work environment in addition to Covid-19 related symptoms and exposures.[8,9] For the current study, we included all participants who completed both SARS-CoV-2 antibody testing and electronic survey forms (N=6,062). The study protocol was approved by the Cedars-Sinai institutional review board and all participants provided written informed consent.

Serologic Assays

All participant biospecimens underwent serology testing by the Cedars-Sinai Department of Pathology and Laboratory Medicine using the Abbott Diagnostics SARS-CoV-2 IgG chemiluminescent microparticle immunoassay assay (Abbott Diagnostics, Abbott Park, IL) performed on an Abbott Diagnostics Architect ci16200 analyzer. The assay reports a signal-to-cutoff ratio (S/CO) corresponding to the relative light units produced by the test sample compared to the relative light units produced by an assay calibrator sample. The manufacturer recommended S/CO ratio of 1.4 was used to assign binary seropositivity status. This cutoff was validated for high specificity (i.e., >99%) ~14 days post symptom onset.[10] The Abbott assay detects antibodies directed against the nucleocapsid (N) antigen of the SARS-CoV-2 virus, which

For peer review only - http://bmjopen.bmj.com/site/about/guidelines.xhtml

assists with packaging the viral genome after replication, and achieves specificity for IgG by incorporating an anti-human IgG signal antibody.

Statistical Analyses

Estimates of Seroprevalence. We conducted a literature review to identify published data (until June 25, 2020) on the sensitivity and specificity of the Abbott Architect SARS-CoV-2 IgG assay, applied in specific populations using the manufacturer's recommended thresholds. We identified a total of 15 studies assessing sensitivity in 2,114 tests and 18 studies reporting specificity in 7,748 tests (**Supplemental Tables 1-2**); we combined this information with data from an additional independent cohort of 60 case and 178 control specimens used to asses sensitivity and specificity, respectively, within the Cedars-Sinai Department of Pathology and Laboratory Medicine. We noted that studies investigating specificity generally assessed samples collected prior to the SARS-CoV-2 pandemic whereas studies reporting sensitivity included specimens from RT-PCR confirmed individuals (see details provided in **Supplemental Tables 1-2**). We restricted our analyses to a referent cohort of tests conducted on samples from individuals who were assayed ≥7 days following symptoms onset to most closely match our cohort sample characteristics and the situational context for study enrollment. We integrated source population-level demographic data, representative of the entire Cedars-Sinai employee base, with data from our enrolled study sample using an Iterative Proportional Fitting procedure (IPF) to estimate the number of eligible employees within each demographic category (with provided population totals considered the target, using constraints derived from our sample).[11] We then fit a Bayesian multilevel hierarchical logistic regression model using RStan,[12,13] including reported age, gender, race/ethnicity and site as coefficients, to model exposure probability (see **Supplemental Methods** for full details). We estimated the seroprevalence within each post-stratified demographic category based on the averaged and weighted value of the expected number of employees within that category.

For peer review only - http://bmjopen.bmj.com/site/about/guidelines.xhtml

Factors Associated with Seroprevalence. Prior to multivariable-adjusted analyses, age and IgG index were transformed by dividing by 10 for interpretability of coefficients in all models. In adjusted analyses, we compared differences between serology status (i.e. antibody positive versus negative) in each variable of interest, grouped into one of three categories: (1) pre-existing demographic and clinical characteristics (e.g. age, gender, ethnicity, race, and self-reported medical comorbidities); (2) Covid-19 related exposures (e.g. self-reported medical diagnosis of Covid-19 illness, household member with Covid-19 illness, number of people living in the home including children, type of home dwelling, etc); and, (3) Covid-19 related response variables (e.g. self-reported fever, chills, dry cough, anosmia, nausea, myalgias, etc.). In multivariable-adjusted analyses, we used logistic and linear models to examine the extent to which the three categories of variables (predictors) may be associated with antibody positive status (primary outcome) in the total sample or IgG antibody level in the subset of persons with positive antibody status (secondary outcome). Initial models were deliberately sparse, adjusting for a limited number of key covariates (e.g. age, gender) and those variables with associations meeting a significance threshold of $P<0.10$ were advanced for inclusion in a final multivariable model with only other variables identified from the sparse regression included. A final separate multivariable model was constructed for each of the 3 categories of variables.

Patient and Public Involvement. Patients and the public were not involved in the development of this study.

For peer review only

RESULTS

The demographic, clinical, exposure, and symptom response characteristics of the study sample are shown in **Table 1**, by antibody test result status; the study sample included individuals whose residence spanned diverse regions across Los Angeles County (**Supplemental Figure 1**). The overall seroprevalence was 4.1% (95% CI 3.1%, 5.7%), with higher estimates seen in younger compared to older individuals and in Hispanics compared to non-Hispanics (**Figure 1** and **Supplemental Table 3**).

In multivariable-adjusted analyses of pre-existing characteristics (**Figure 2** and **Supplemental Table 4**), the main factors significantly associated with greater odds of seropositive status were Hispanic ethnicity (OR 1.80 [95% CI 1.31, 2.46], P<0.001), and African American race (1.72 [1.03, 2.89], P=0.04), compared to non-Hispanic Whites. The main factors associated with lower odds of being seropositive were older age (0.81 [0.71, 0.92] per age decade, P=0.001), and a history of asthma (0.48 [0.26, 0.80], P=0.009). Among all seropositive persons, hypertension was significantly associated with higher antibody level (beta 0.11 [SE 0.04] per 10-unit increment in the IgG index, P=0.011).

In multivariable-adjusted analyses of Covid-19 related exposures (**Figure 3** and **Supplemental Table 5**), the factors significantly associated with greater odds of seropositive status were having had a medical diagnosis of Covid-19 (7.78 [5.73, 10.56], P<0.001) and a household member previously diagnosed with Covid-19 (9.42 [5.50, 16.13], P<0.001), with a similar trend observed for working in a location where Covid-19 patients are treated (1.61 [1.18, 2.18], P=0.002]. Among seropositive individuals, having a medical diagnosis of Covid-19 was associated with higher antibody level. Notably, dwelling type, number of people in the home, and having children or common domestic pets were not associated with either seroprevalence or antibody titer.

For peer review only - http://bmjopen.bmj.com/site/about/guidelines.xhtml

In multivariable-adjusted analyses of Covid-19 response variables (**Figure 4** and **Supplemental Table 6**), the strongest self-reported symptom associated with greater odds of seropositive status was anosmia (11.53 [7.51, 17.70], P<0.001). Other symptoms associated with the presence of antibodies included dry cough, loss of appetite, and myalgias. Notably, the symptoms associated with lower odds of seropositive status included sore throat and rhinorrhea. Dyspnea was significantly associated with higher titer levels in seropositive individuals (beta 0.13 [SE 0.04], P=0.001).

Significantly predictive pre-existing characteristics, exposures and symptoms from the prior models were subsequently analyzed together. In multivariable analysis, all included predictors, except for dry cough, myalgias and fatigue remained significantly associated with the presence of antibodies. Predictors which remained significantly associated with higher antibody levels included hypertension (beta 0.09 [SE 0.04], P=0.031), prior Covid-19 diagnosis (beta 0.09 [SE 0.03], P=0.002), working in a Covid unit (beta 0.07 [SE 0.03], P=0.008), and dyspnea (beta 0.07 [SE 0.03], P=0.015) (**Figure 5** and **Supplemental Table 7**).

For peer review only - http://bmjopen.bmj.com/site/about/guidelines.xhtml

DISCUSSION

In a large diverse healthcare employee cohort of over 6,000 adults in Los Angeles, we observed a seroprevalence rate of 4.1%, which when accounting for published test characteristics, may range from 3.1% to 5.7%. Seroprevalence varied across demographic, clinical, exposure and symptom based characteristics. Specifically, factors significantly associated with presence of IgG antibodies included younger age, Hispanic ethnicity, and African-American race, as were exposure related factors including the presence of either a personal or household member having a prior medical diagnosis of Covid-19. Among self-reported symptoms, anosmia was most strongly associated with the presence of antibodies, with positive associations also noted for fever, dry cough, anorexia, and myalgias. The size and diversity of this study population, combined with robust survey and modeling techniques, provide a more vibrant picture of the population at highest risk for Covid-19 infection, risks of various potential exposures and symptoms that should alter patients to potential illness.

Most prior seroprevalence studieshave focused on cohorts that included healthcare workers predominantly involved in direct or indirect patient care, persons living within a circumscribed region with high viral exposure rates, or larger geographic areas from which motivated individuals could voluntarily enroll into community screening programs.[14,15] Given that completely unbiased population-scale sampling for seroprevalence studies remains a logistical challenge, we used a sampling approach that involved open enrollment and convenient access to testing facilities made available to all employees working across multiple sites of a large healthcare system; this approach was intended to broadly capture individuals with both patient-related exposures and community-related exposures, while also representative of a relatively wide geographic area in and around Los Angeles County. Although limited to persons who are generally healthy and able to be employed, our study cohort included individuals representing a diversity of demographic

For peer review only - http://bmjopen.bmj.com/site/about/guidelines.xhtml

characteristics including ethnicity and race – leading to findings that reflect the disparities that have been persistently observed and reported for Covid-19 infection rates in our local communities.

Consistent with findings from studies in healthcare workers, seroprevalence patterns in our cohort indicate exposure from not only the work environment but also from the home environment and likely unmeasured community-based factors.[16] It has been well reported that minority populations, particularly African Americans and Hispanics, have been disproportionately effected by the Covid-19 panedmic.[17-19] Our study is consistent with these prior findings, but demonstrates that such differences exist even when all participants work not just in the same field, but for the same organization. Such a finding may indicate that community and non-work related environmental factors are likely playing a significant role in the spread of Covid-19 among certain minority populations. Even after controlling for a medical diagnosis of Covid-19, African American race and Hispanic ethnicity remained risk factors for antibody positivity. The persistence of thse racial and ethnic disparities may represent structural barriers to care or societally mediated risk. Geographic clustering by race and ethnicity in housing, shopping and social gatherings may be one such factor, while socioeconomic status and ability to self-isolate outside of work likely also contribute.[20-22]

No self-reported pre-existing medical conditions were significantly associated with antibody positivity, indicating that infection itself is agnostic to baseline health. In fact, asthma was negatively associated with the presence of antibodies, or at least antibody levels above the current threshold we use for positivity. While reactive airway disease is unlikely a protective factor against Covid-19, participants with such conditions may be more likely to deligently follow social distancing guidelines and practice better adherence to hand hygiene and use of personal protective equipment. Hypertension was the only medical condition associated with higher SARS-

For peer review only - http://bmjopen.bmj.com/site/about/guidelines.xhtml

CoV-2 antibody levels. It remains unclear as to what physiologic mechanism may contribute to this finding, however, unmeasured confounding variables, such as medications or renal disease may function as mediating factors. Further studies will be needed to both verify and elucidate this finding.

Also concordant with prior studies, we found that anosmia was the single strongest symptom associated with SARS-CoV-2 IgG antibody presence.[23-25] Interestingly, neither dyspnea nor diarrhea, two commonly cited symptoms, demonstrated a significant association in multivariable analysis.[26,27] This is likely related to the non-specific nature of these symptoms, which are common to multiple viral and non-viral etiologies. Importantly, dyspnea was associated with a higher antibody level among those with anti-SARS-CoV-2 antibodies, suggesting that dyspnea related to Covid-19 may drive a more robust humoral immune response, potentially related to more severe infection. These findings are concordant with the known phenomenon of proportionate adaptive immune response to higher doses of antigenic stress.[28] The extent to which the generation of measurably higher antibody levels could confer immunity to a larger degree or for a longer duration of time remains unknown. Interestingly, prior studies have demonstrated lower antibody levels among exposed, asymptomatic individuals, a phenomena which may be attributable to a highly efficient cell mediated immune response.[29] It has be suggested that higher T-cell levels, whether virus specific or otherwise, may play a role in this finding, however, further research is required.[30,31]

Further expanding from prior studies, we investigated and observed several factors that appeared notably unassociated with seroprevalence. In particular, we found that recent travel, type of home, and number of people living in the home were not associated with an antibody-based measure of SARS-CoV-2 exposure. The presence of antibodies was also not related to youth or children in the home, or to having domestic pets such as cats or dogs. Although far from definitive, these

For peer review only - http://bmjopen.bmj.com/site/about/guidelines.xhtml
For peer review only

results suggest that these factors do not play an important role in mediating potentially meaningful viral exposure in the communities represented by our study cohort.

Several limitations of this study merit consideration. Of the employees actively employed at our multi-site institution, only a proportion of all eligible participants enrolled; nonetheless, the sample size of the cohort was large, diverse, and representative of the source sample.[7] Our seroprevalence estimates were based on using a validated assay of only IgG antibodies; assays of IgM antibodies may offer complementary information in future studies. Data collected on medical history, exposures, and symptoms were all self-reported, similar to approaches used in prior studies. We were unable to completely verify prior Covid-19 illness using viral test results in part given lack of universally available testing for all individuals, particularly those with minimal to no symptoms.

In conclusion, in a highly diverse population of healthcare workers, demographic factors associated with Covid-19 antibody positivity indicate potential factors outside of the workplace associated with SARS-CoV-2 exposure, although these do not appear related to the number of people or to the presence of children in the home. Further, while for dyspnea may be a marker of more severe disease among those with Covid-19, it's presence alone does not indicate infection.

DATA AVAILABILITY

The data that support the findings of this study are available from Cedars-Sinai Medical Center, upon reasonable request. The data are not publicly available due to the contents including information that could compromise research participant privacy/consent.

AUTHOR CONTRIBUTIONS

All authors contributed to and have approved the final manuscript. JEE and SC took part in conception, data collection, data analysis, drafting of the manuscript, and editing of the

For peer review only - http://bmjopen.bmj.com/site/about/guidelines.xhtml

manuscript. GJB took part in data analysis, drafting of the manuscript, and editing of the manuscript. CMA took part in conception, data analysis, and editing of the manuscript. MAl., MAr., and JFB took part in editing of the manuscript. AHB, AB took part in data collection, data analysis, and editing of the manuscript. PB, WH, MH, and RVR took part in data collection and data analysis. JCF, SJ, EHK, PBM, TTN, MM MAR, and SSt. took part in data collection. JDG, SKH, MJ, YL, EL, DPBM, NM, and WGT took part in data analysis and editing of the manuscript. MK, DL, AM, KR, CER, SSh., and NS, took part in data analysis. KS took part in data collection, data analysis, drafting of the manuscript and editing of the manuscript. JEVE and JGB took part in conception, data analysis, drafting of the manuscript, and editing of the manuscript.

ACKNOWLEDGEMENTS

We are grateful to all the front-line healthcare workers in our healthcare system who continue to be dedicated to delivering the highest quality care for all patients.

FUNDING

This work was supported in part by Cedars Sinai Medical Center and the Erika J. Glazer Family Foundation.

COMPETING INTERESTS

The authors declare that they have no competing interests.

For peer review only - http://bmjopen.bmj.com/site/about/guidelines.xhtml

REFERENCES

1. Bryant JE, Azman AS, Ferrari MJ, et al. Serology for SARS-CoV-2: Apprehensions, opportunities, and the path forward. *Science Immunology.* 2020;5(47):eabc6347.

2. Health CfDaR. Policy for Diagnostic Tests for Coronavirus Disease-2019 during the Public Health Emergency. In: Administraction FaD, ed: Dockets Management; 2020.

3. Nuccetelli M, Pieri M, Grelli S, et al. SARS-CoV-2 infection serology: a useful tool to overcome lockdown? *Cell Death Discov.* 2020;6:38.

4. Petherick A. Developing antibody tests for SARS-CoV-2. *Lancet (London, England).* 2020;395(10230):1101-1102.

5. Mallapaty S. Will antibody tests for the coronavirus really change everything? *Nature.* 2020;580(7805):571-572.

6. Espejo AP, Akgun Y, Al Mana AF, et al. Review of Current Advances in Serologic Testing for COVID-19. *Am J Clin Pathol.* 2020.

7. Ebinger JE, Botwin GJ, Albert CM, et al. An Opportune and Relevant Design for Studying the Health Trajectories of Healthcare Workers. *medRxiv.* 2020:2020.2006.2030.20140046.

8. Harris PA, Taylor R, Thielke R, Payne J, Gonzalez N, Conde JG. Research electronic data capture (REDCap)—A metadata-driven methodology and workflow process for providing translational research informatics support. *Journal of Biomedical Informatics.* 2009;42(2):377-381.

9. Harris PA, Taylor R, Minor BL, et al. The REDCap consortium: Building an international community of software platform partners. *J Biomed Inform.* 2019;95:103208.

10. Bryan A, Pepper G, Wener MH, et al. Performance Characteristics of the Abbott Architect SARS-CoV-2 IgG Assay and Seroprevalence in Boise, Idaho. *J Clin Microbiol.* 2020.

11. Barthélemy J, Suesse T. mipfp: An R Package for Multidimensional Array Fitting and Simulating Multivariate Bernoulli Distributions. *2018.* 2018;86(Code Snippet 2):20.

12. *RStan: the R interface to Stan. R package version 2.19.3* [computer program]. 2020.

13. Carpenter B, Gelman A, Hoffman MD, et al. Stan: A Probabilistic Programming Language. *2017.* 2017;76(1):32.

14. Mughal MS, Kaur IP, Patton CD, Mikhail NH, Vareechon C, Granet KM. The prevalence of severe acute respiratory coronavirus virus 2 (SARS-CoV-2) IgG antibodies in intensive care unit (ICU) healthcare personnel (HCP) and its implications-a single-center, prospective, pilot study. *Infect Control Hosp Epidemiol.* 2020:1-2.

For peer review only - http://bmjopen.bmj.com/site/about/guidelines.xhtml

15. Madsen T, Levin N, Niehus K, et al. Prevalence of IgG antibodies to SARS-CoV-2 among emergency department employees. *Am J Emerg Med.* 2020:S0735-6757(0720)30306-30305.

16. Steensels D, Oris E, Coninx L, et al. Hospital-Wide SARS-CoV-2 Antibody Screening in 3056 Staff in a Tertiary Center in Belgium. *JAMA.* 2020.

17. Chowkwanyun M, Reed AL, Jr. Racial Health Disparities and Covid-19 - Caution and Context. *N Engl J Med.* 2020;383(3):201-203.

18. Rentsch CT, Kidwai-Khan F, Tate JP, et al. Covid-19 by Race and Ethnicity: A National Cohort Study of 6 Million United States Veterans. *medRxiv.* 2020.

19. Tai DBG, Shah A, Doubeni CA, Sia IG, Wieland ML. The Disproportionate Impact of COVID-19 on Racial and Ethnic Minorities in the United States. *Clin Infect Dis.* 2020.

20. Turner-Musa J, Ajayi O, Kemp L. Examining Social Determinants of Health, Stigma, and COVID-19 Disparities. *Healthcare (Basel).* 2020;8(2).

21. Thakur N, Lovinsky-Desir S, Bime C, et al. The Structural and Social Determinants of the Racial/Ethnic Disparities in the U.S. COVID-19 Pandemic: What's Our Role? *Am J Respir Crit Care Med.* 2020.

22. Raifman MA, Raifman JR. Disparities in the Population at Risk of Severe Illness From COVID-19 by Race/Ethnicity and Income. *Am J Prev Med.* 2020;59(1):137-139.

23. Lechien JR, Chiesa-Estomba CM, De Siati DR, et al. Olfactory and gustatory dysfunctions as a clinical presentation of mild-to-moderate forms of the coronavirus disease (COVID-19): a multicenter European study. *Eur Arch Otorhinolaryngol.* 2020;277(8):2251-2261.

24. Tong JY, Wong A, Zhu D, Fastenberg JH, Tham T. The Prevalence of Olfactory and Gustatory Dysfunction in COVID-19 Patients: A Systematic Review and Meta-analysis. *Otolaryngol Head Neck Surg.* 2020;163(1):3-11.

25. Lee DJ, Lockwood J, Das P, Wang R, Grinspun E, Lee JM. Self-reported anosmia and dysgeusia as key symptoms of coronavirus disease 2019. *CJEM.* 2020:1-8.

26. Zhu J, Zhong Z, Ji P, et al. Clinicopathological characteristics of 8697 patients with COVID-19 in China: a meta-analysis. *Fam Med Community Health.* 2020;8(2).

27. Kopel J, Perisetti A, Gajendran M, Boregowda U, Goyal H. Clinical Insights into the Gastrointestinal Manifestations of COVID-19. *Dig Dis Sci.* 2020;65(7):1932-1939.

28. DiazGranados CA, Dunning AJ, Kimmel M, et al. Efficacy of High-Dose versus Standard-Dose Influenza Vaccine in Older Adults. *New England Journal of Medicine.* 2014;371(7):635-645.

For peer review only

For peer review only - http://bmjopen.bmj.com/site/about/guidelines.xhtml

29. Long QX, Tang XJ, Shi QL, et al. Clinical and immunological assessment of asymptomatic SARS-CoV-2 infections. *Nat Med.* 2020.

30. Grifoni A, Weiskopf D, Ramirez SI, et al. Targets of T Cell Responses to SARS-CoV-2 Coronavirus in Humans with COVID-19 Disease and Unexposed Individuals. *Cell.* 2020;181(7):1489-1501 e1415.

31. Weiskopf D, Schmitz KS, Raadsen MP, et al. Phenotype and kinetics of SARS-CoV-2-specific T cells in COVID-19 patients with acute respiratory distress syndrome. *Sci Immunol.* 2020;5(48).

For peer review only

Table 1. Characteristics of the Study Sample

	Antibody Negative N=5850	Antibody Positive N=212
**Pre-Existing Characteristics**		
Age, mean (SD)	41.6 (12.0)	38.5 (11.2)
Male gender (%)	1876 (32)	73 (34)
Hispanic ethnicity (%)	1097 (19)	62 (29)
Race (%)		
Asian	1809 (31)	57 (27)
Black	354 (6)	18 (8)
White	2938 (50)	104 (49)
Other	749 (13)	33 (16)
Current smoker (%)	99 (2)	3 (1)
Current vape user (%)	83 (1)	4 (2)
Medical conditions (%)		
Asthma	733 (13)	14 (7)
Immune	228 (4)	4 (2)
Cancer	195 (4)	3 (1)
Cardiovascular	127 (2)	2 (1)
Chronic Obstructive Pulmonary Disease	84 (2)	0 (0)
Diabetes Mellitus	371 (7)	8 (4)
Hypertension	967 (17)	26 (13)
**Potential Covid-19 Related Exposures**		
Personal diagnosis of Covid-19 (%)	530 (9)	104 (50)
Household member diagnosed with Covid-19 (%)	51 (1)	31 (15)
Domestic travel since September 2019 (%)	2127 (37)	54 (26)
International travel since September 2019 (%)	1324 (23)	44 (21)
Regular contact with Covid-19 patients (%)	1358 (24)	86 (41)
Work on a unit housing/caring for Covid-19 patients (%)	1600 (27)	93 (44)
Type of dwelling (%)		
Apartment	2636 (46)	93 (44)
House	2914 (51)	107 (51)
Other	216 (4)	9 (4)

For peer review only

For peer review only - http://bmjopen.bmj.com/site/about/guidelines.xhtml

No. people living in the home, mean (SD)	2.3 (1.7)	2.4 (1.8)
Any persons in the home under age 18 years (%)	1843 (32)	65 (31)
Any persons in the home under age 12 years (%)	1467 (25)	51 (24)
Cats as household pets (%)	783 (13)	27 (13)
Dogs as household pets (%)	2189 (37)	95 (45)
**Potential Covid-19 Related Responses**		
Fever (%)	497 (9)	87 (43)
Chills (%)	683 (12)	95 (46)
Headache (%)	2061 (36)	126 (61)
Conjunctivitis (%)	162 (3)	14 (7)
Anosmia (%)	252 (4)	107 (52)
Nasal congestion (%)	1611 (28)	104 (51)
Rhinorrhea (%)	1493 (26)	82 (41)
Dry cough (%)	1235 (22)	108 (53)
Productive cough (%)	542 (10)	50 (25)
Sore throat (%)	1368 (24)	81 (40)
Chest pain (%)	453 (8)	45 (22)
Dyspnea (%)	604 (11)	66 (33)
Anorexia (%)	390 (7)	78 (38)
Nausea (%)	657 (12)	52 (25)
Vomiting (%)	188 (3)	15 (8)
Diarrhea (%)	853 (15)	59 (29)
Myalgias (%)	1033 (18)	117 (58)
Fatigue (%)	1447 (25)	135 (66)
Skin changes (%)	261 (5)	15 (8)
Stroke symptoms (%)	35 (1)	3 (2)
Sneezing (%)	1863 (33)	94 (47)

For peer review only - http://bmjopen.bmj.com/site/about/guidelines.xhtml

FIGURE LEGEND

Figure 1. Seroprevalence Overall and by Subgroup

Figure 2. Pre-Existing Factors Associated with SARS-CoV-2 Seroprevalence

Figure 3. Potential COVID Illness Exposure Related Factors Associated with SARS-CoV-2

Figure 4. Potential COVID Illness Response Factors Associated with SARS-CoV-2 Seroprevalence

Figure 5. Factors Associated with SARS-CoV-2

For peer review only

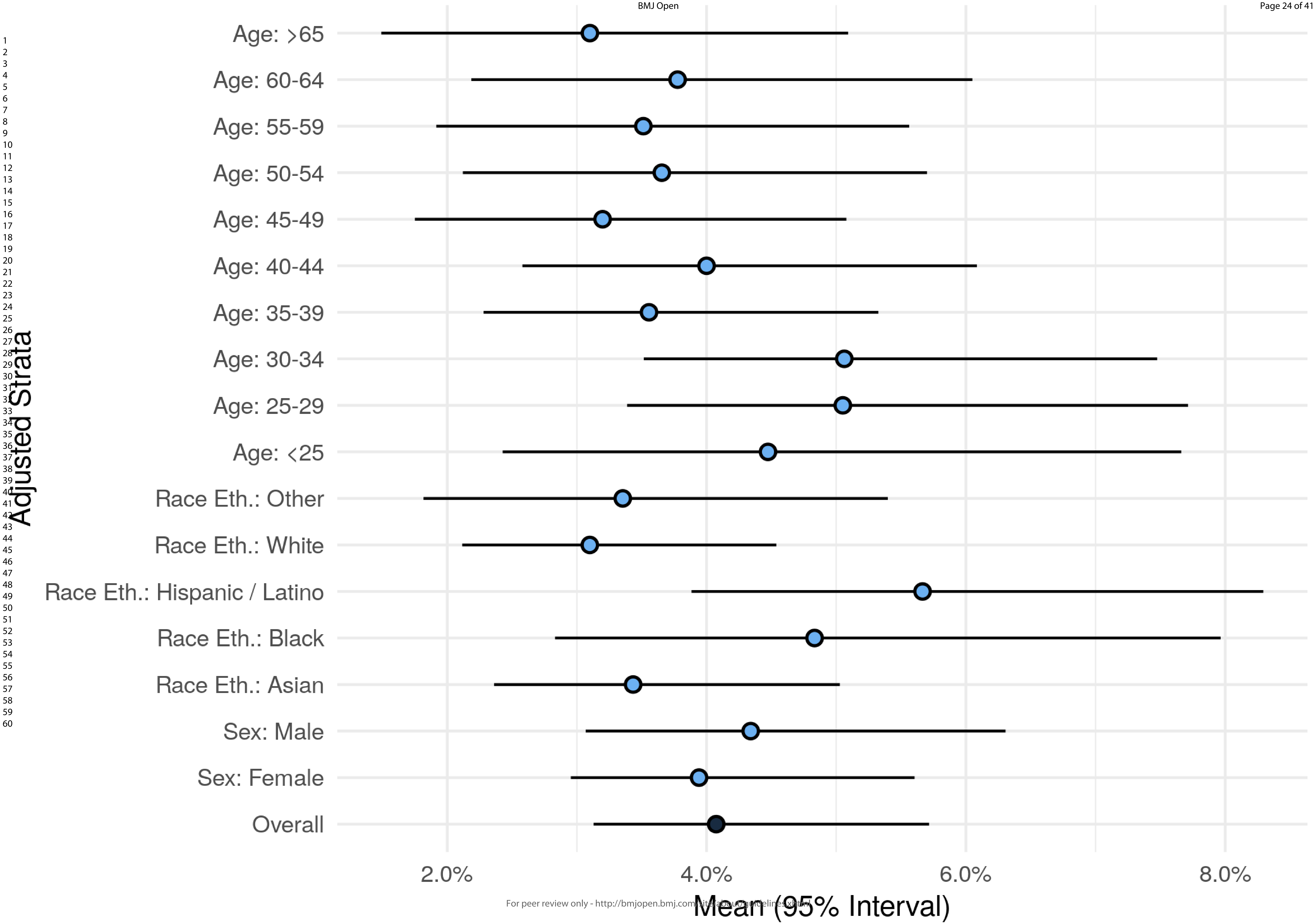
Age: >65
Age: 60-64
Age: 55-59
Age: 50-54
Age: 45-49
Age: 40-44
Age: 35-39
Age: 30-34
Age: 25-29
Age: <25
Race Eth.: Other
Race Eth.: White
Race Eth.: Hispanic / Latino
Race Eth.: Black
Race Eth.: Asian
Sex: Male
Sex: Female
Overall
Adjusted Strata
2.0%
4.0%
6.0%
8.0%
Mean (95% Interval)

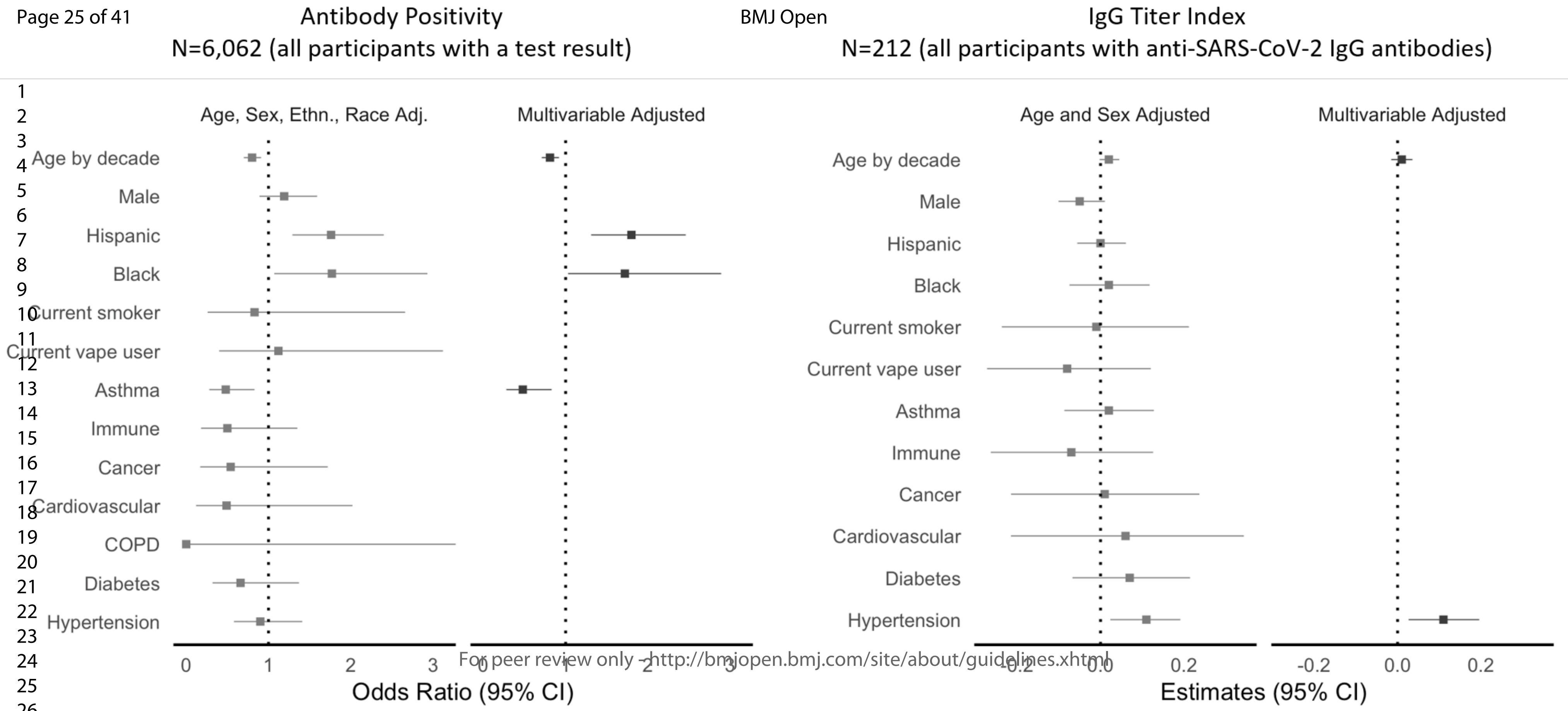
Antibody Positivity
N=6,062 (all participants with a test result)
IgG Titer Index
N=212 (all participants with anti-SARS-CoV-2 IgG antibodies)
Age, Sex, Ethn., Race Adj.
Multivariable Adjusted
Age and Sex Adjusted
Multivariable Adjusted
Age by decade
Male
Hispanic
Black
Current smoker
Current vape user
Asthma
Immune
Cancer
Cardiovascular
COPD
Diabetes
Hypertension
0
1
2
3
Odds Ratio (95% CI)
-0.2
0.0
0.2
Estimates (95% CI)

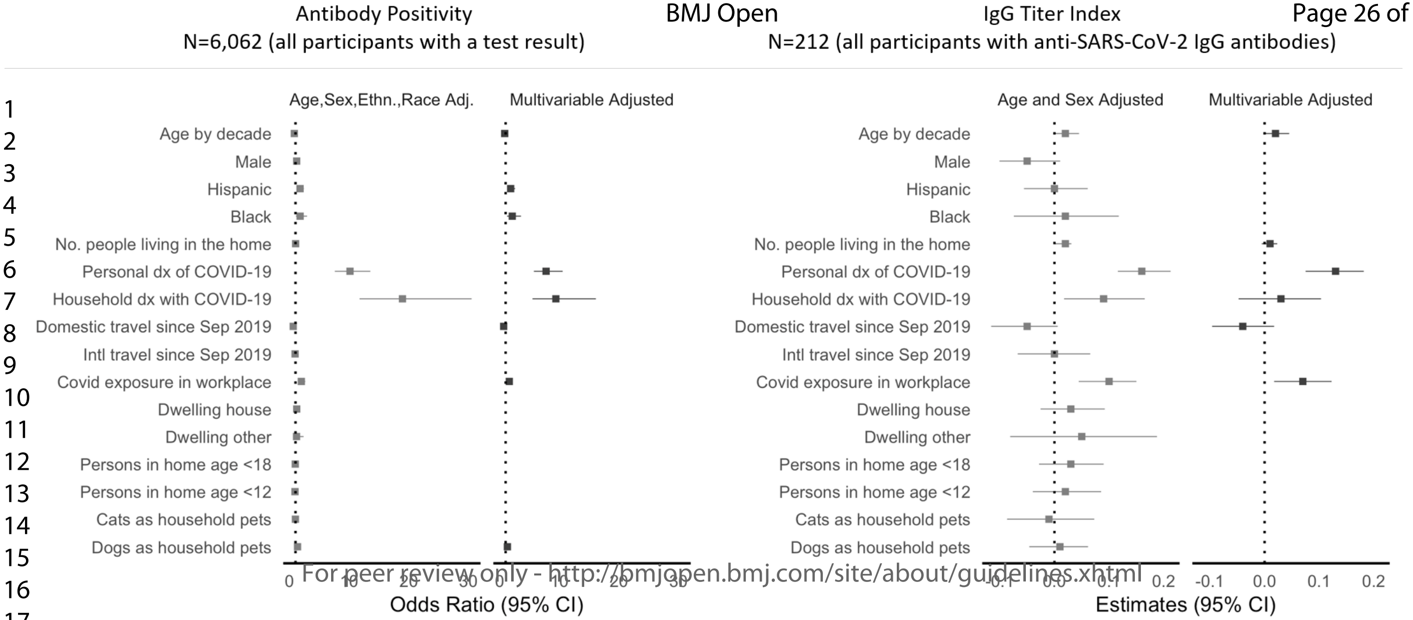

For peer review only - http://bmjopen.bmj.com/site/about/guidelines.xhtml

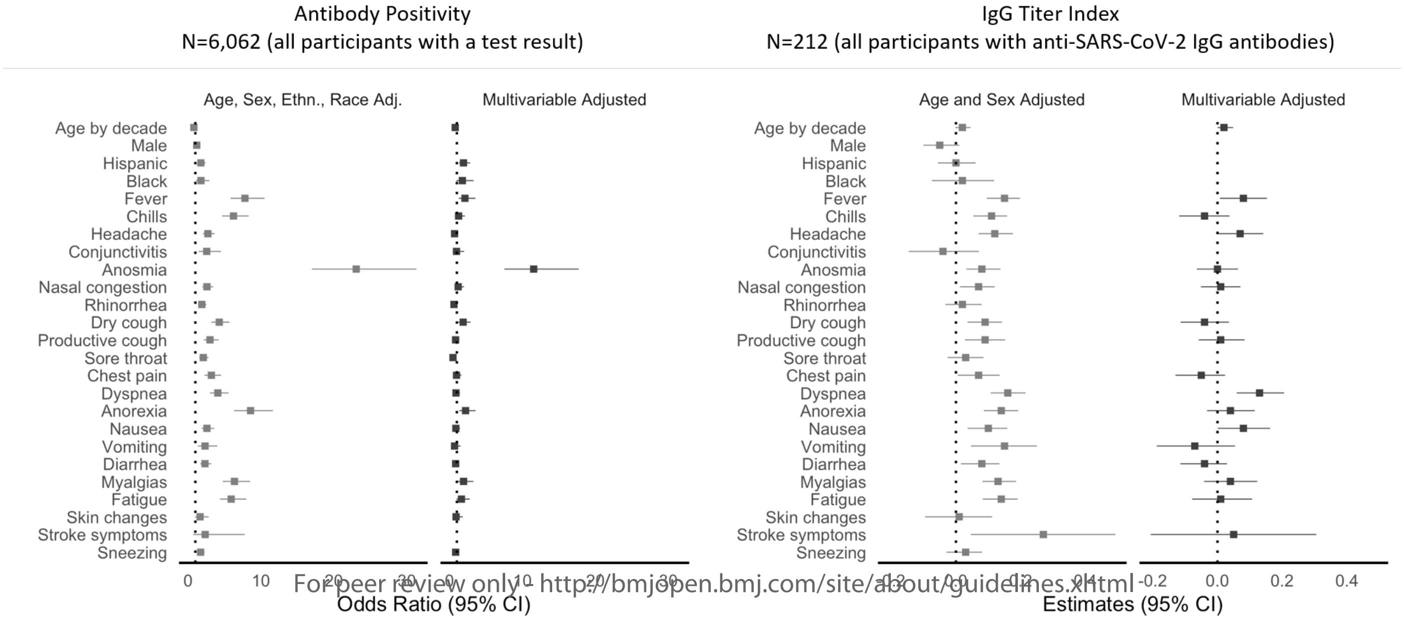

For peer review only - http://bmjopen.bmj.com/site/about/guidelines.xhtml

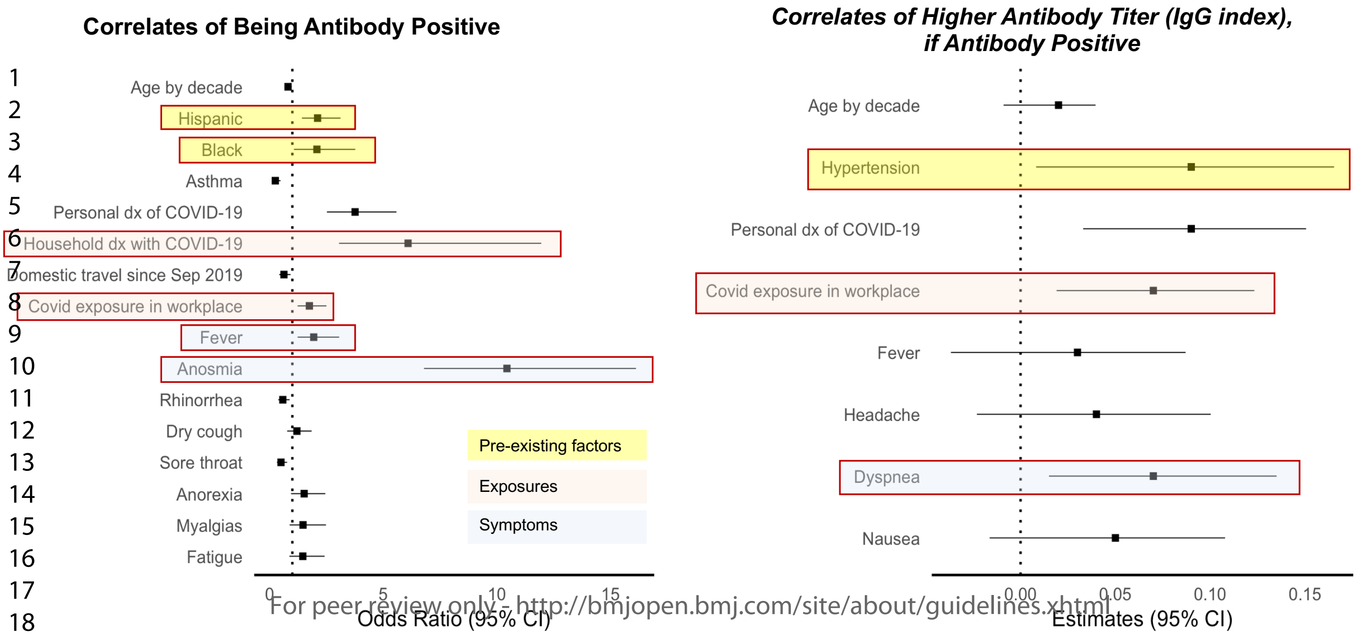

For peer review only - http://bmjopen.bmj.com/site/about/guidelines.xhtml

SUPPLEMENTAL MATERIAL

SARS-CoV-2 Seroprevalence Across a Diverse Cohort of Healthcare Workers

For peer review only

For peer review only - http://bmjopen.bmj.com/site/about/guidelines.xhtml

Supplemental Table 1. Prior Studies Reporting Sensitivity for the Abbott Architect SARS-CoV-2 IgG Assay[1-14]

Author	Positive Tests	Total Tests	Sample Description
Abbott[1]	109	115	Using data from >=8 days post symptom onset and including 5 immunocompromised samples. Positive subjects who tested positive for SARS-CoV-2 by a polymerase chain reaction (PCR) method and who also presented with Covid-19 symptoms.
Bryan and Pepper et al.[2]	668	689	Serum specimens sent for clinical testing from persons who tested RT-PCR positive for SARS-CoV -2 during March and April 2020.
Ng and Goldgof and Shy and Levine and Balcerek and Bapat et al.[15]	328	382	Received care at adult inpatient units or clinics and were RT PCR positive for SARS-CoV-2 from nasopharyngeal and/or oropharyngeal swab testing. Using combined data from immunocompromised individuals. Combining data from Day 8 + PSO.
Ekelund et al.[4]	17	20	Serum samples from 16 individuals that prior to serum sampling had tested RT-PCR positive for SARS-CoV-2 in nasopharyngeal and/or pharyngeal swabs. The interval between onset of Covid-19 symptoms to serum sample collection ranged from 18 to 52 days (median 38 days).
Phipps and SoRelle et al.[5]	10	21	8 or more days PSO. suspected Covid-19 cases with PCR-based nasopharyngeal swab testing on the m2000 Abbott RealTime SARS Cov-2 assay or the Abbott ID NOWTM Covid-19 assay.
Phipps and SoRelle et al.[5]	10	13	Indeterminate days from PSO. Suspected Covid-19 cases with PCR-based nasopharyngeal swab testing on the m2000 Abbott RealTime SARS Cov-2 assay or the Abbott ID NOWTM Covid-19 assay.
Chew et al.[6]	65	96	Used COVID pts at different stage of disease: results based on 7 + PSO disease stage: ≤6 days (7/81), at 7–13 days (17/39), at 14–20 days (21/25), and at ≥21 days (27/32)
Theel et al.[7]	78	84	Anti-SARS-CoV-2 IgG assay sensitivity in convalescent sera and in individual patients tested ≥15 days post-symptom onset or first positive SARS-CoV-2 RT-PCR result
Theel et al.[7]	123	175	Included inpatients and outpatients PCR positive from >= 8 PSO
Kohmer et al.[8]	35	45	From 45 pts with positive PCR
Stroemer et al.[9]	33	34	34 sera obtained from 26 patients between four and 60 days (median 19 days) after a positive real-time RT-PCR.
Nicol et al.[10]	115	141	141 serum from 82 patients with positive PCR varying days from PSO
Dellière et al.[11]	86	95	Serum samples (n=95) from patients at least 10 days from symptoms onset or positive PCR
Perkmann et al. [12]	55	65	65 Covid-19 donors/patients with a symptom onset to analysis time of ≥14 days
Mueller et al.[13]	7	8	8 RT-PCR positive individuals
Tang et al.[14]	56	71	103 specimens from 48 patients with PCR confirmed SARS-CoV-2 infections from NP, OP or lower respiratory swab. Reported positive results from time from PCR: 0d=12/27, 1-3d= 8/15, 3-7d=13/22, 8-13d=16/23, >14d=13/16. and reported positive from symptoms onset: <3d= 0/12, 3-7d=6/20, 8-13=11/23, >14d=45/48
Cedars-Sinai Department of Pathology and Laboratory Medicine*	53	60	All COVID Positive subjects were selected by three criteria: (1) Presentation to Cedars-Sinai Medical Center with symptoms consistent with infection by SARS-CoV-2 virus; (2) Were PCR positive for SARS-CoV-2 viral RNA in at least one nasopharyngeal sample; (3) Had EDTA or heparin plasma available for testing which was collected 8 or more days after onset of symptoms according to physician's notes in the medical record.

*Unpublished data

For peer review only - http://bmjopen.bmj.com/site/about/guidelines.xhtml

Supplemental Table 2. Prior Studies Reporting Specificity for the Abbott Architect SARS-CoV-2 IgG Assay

Author	Negative Test	Total Tests	Sample source
Abbott[1]	1066	1070	997 specimens were collected prior to September 2019<br>73 specimens were collected in 2020 with signs of respiratory illness and Covid-19 RT-PCR negative
Bryan and Pepper et al.[2]	1019	1020	Serum samples from 2018 and 2019
Jääskeläinen et al.[16]	79	81	Serum samples from 2018 and 2019
Ng, Goldgof, Shy, Levine, Balcerek and Bapat et al.[15]	1011	1013	US blood donors prior to the Covid-19 pandemic
	234	235	Plasma samples from 163 Covid-19 RT-PCR negative
Ekelund et al.[4]	100	100	Pre-pandemic samples from 2018
Phipps and SoRelle et al.[5]	656	656	240 samples collected prior to the Covid-19 pandemic (blood donors September through November 2019), and an additional 416 healthy donors without recent illness collected from March to April, 2020
	91	91	23 CMV IgG positive, 8 prior Flu A+, 7 Flu B+, 6 RSV+, 47 endemic coronavirus samples (January 1, 2015- September 30, 2019) with normal or high levels of total IgG with no infusion of intravenous immunoglobulin in the preceding 3 months
	29	29	Lupus patients that were positive for multiple autoantibodies (100% ANA, 62% anti-dsDNA, 75% anti-U1RNP, 55% anti-Sm, 34% anti-Ro52, 170 and 24% anti-La) 2004-2007
	20	20	Rheumatoid arthritis patients positive for rheumatoid factor (85% were also anti-CCP positive) 2011-2014
	96	97	Patients with Covid-19 RT-PCR negative
Chew et al.[6]	163	163	
Theel et al.[7]	149	149	Healthy samples from 2018
	104	105	Samples negative for Covid-19 but positive for antibodies from other respiratory virus or bacteria (2020)
Kohmer et al.[8]	35	35	
Ströemer et al.[9]	99	100	100 archived samples from winter and summer seasons
Nicol et al.[10]	57	57	52 patients with symptoms of Covid-19 but negative RT-PCR
	49	50	Residual serum samples collected before Covid-19 in Mar 2019
	25	25	Samples with potential cross-reaction to Covid-19
	10	10	Samples from pregnant women
	10	10	Samples with positive rheumatoid factor
Paiva et al.[17]	1055	1059	Combining random Covid-19 samples during March 2020 (negative RT-PCR), pre-pandemic samples, and pre pandemic prenatal samples. False positive tests (4) were from samples with Hepatitis A, Hepatitis B, Rheumatoid Factor and anti-DNA
Brecher et al.[18]	20	20	Patients with PCR Documented Common Cold

For peer review only

Dellière et al.[11]	42	42	42 patients from pre-pandemic. 14 healthy, 16 endemic corona virus, 1 rhino virus, 1 metapneumovirus, 1 influenza A, 1 RSV. 1 HIV, 1 Hepatitis B. 1 toxoplasmosis. 2 Rheumatoid Factor
Perkmann et al.[12]	490	494	Cross selection of Viennese population, LEAD study between November and April to enrich seasonal infections
	299	302	Healthy voluntary donors
	356	358	Patients with rheumatic disease
Mueller et al.[13]	26	26	Patients with suspected Covid but negative neutralization test and PCR
Tang et al.[14]	152	153	80 patients symptomatic for Covid-19 but negative RT-PCR. 50 samples collected in 2015. 5 samples with other corona virus infection. 4 samples with Influenza A or B. 14 samples with interfering antibiotics.
Cedars-Sinai Department of Pathology and Laboratory Medicine*	178	178	Samples collected prior to 1/1/2020

For peer review only

Supplemental Table 3. Prevalence of Measurable SARS-CoV-2 IgG Antibody in the Study Sample

	Mean (95% CI)
**Overall**	**4.1 (3.1, 5.7)**
Sex: Female	3.9 (3.0, 5.6)
Sex: Male	4.3 (3.1, 6.3)
Age: <25	4.5 (2.4, 7.7)
Age: 25-29	5.1 (3.4, 7.7)
Age: 30-34	5.1 (3.5, 7.5)
Age: 35-39	3.6 (2.3, 5.3)
Age: 40-44	4 (2.6, 6.1)
Age: 45-49	3.2 (1.8, 5.1)
Age: 50-54	3.7 (2.1, 5.7)
Age: 55-59	3.5 (1.9, 5.6)
Age: 60-64	3.8 (2.2, 6.0)
Age: >65	3.1 (1.5, 5.1)
Race Eth.: Asian	3.4 (2.4, 5.0)
Race Eth.: Black	4.8 (2.8, 8.0)
Race Eth.: Hispanic / Latino	5.7 (3.9, 8.3)
Race Eth.: Other	3.4 (1.8, 5.4)
Race Eth.: White	3.1 (2.1, 4.5)

For peer review only

Supplemental Table 4. Pre-Existing Factors Associated with SARS-CoV-2 Seroprevalence

Predictors	Outcome: Antibody Positive N=6,062 (everybody with a test result)				Outcome: IgG index (divided by 10) N=212 (everybody with a test result)			
	Model 1		Model 2		Model 3		Model 4	
	OR (95% CI)	P	OR (95% CI)	P	Est (SE)	P	Est (SE)	P
Age (per decade)	0.8 (0.7, 0.91)	0.001	0.81 (0.71, 0.92)	0.001	0.02 (0.01)	0.07	0.01 (0.01)	0.43
Male Sex	1.19 (0.89, 1.59)	0.24			-0.05 (0.03)	0.11		
Hispanic Ethnicity	1.76 (1.28, 2.40)	<0.001	1.8 (1.31, 2.46)	<0.001	0 (0.03)	0.93		
African American Race	1.77 (1.07, 2.93)	0.027	1.72 (1.03, 2.89)	0.04	0.02 (0.05)	0.66		
Smoking	0.83 (0.26, 2.66)	0.76			-0.01 (0.11)	0.91		
Vaping	1.12 (0.4, 3.12)	0.82			-0.08 (0.1)	0.45		
Asthma	0.48 (0.28, 0.83)	0.009	0.48 (0.28, 0.8)	0.009	0.02 (0.05)	0.71		
Immune Disorder	0.5 (0.18, 1.35)	0.17			-0.07 (0.1)	0.49		
Cancer	0.54 (0.17, 1.72)	0.29			0.01 (0.12)	0.92		
Cardiovascular Disease	0.49 (0.12, 2.02)	0.33			0.06 (0.14)	0.65		
Chronic Obstructive Pulmonary Disease	0 (0, Inf)	0.97						
Diabetes Mellitus	0.66 (0.32, 1.37)	0.26			0.07 (0.07)	0.31		
Hypertension	0.9 (0.58, 1.41)	0.64			0.11 (0.04)	0.013	0.11 (0.04)	0.011

Model 1 is adjusted for age, sex, ethnicity, race.
Model 2 is adjusted for anything that was significant in Model 1 to a P<0.10.

Model 3 is for age, sex
Model 4 is adjusted for anything that was significant in Model 3 to a P<0.10.

For peer review only - http://bmjopen.bmj.com/site/about/guidelines.xhtml

Supplemental Table 5. Potential COVID Illness Exposure Related Factors Associated with SARS-CoV-2 Seroprevalence

	Outcome: Antibody Positive N=6,062 (everybody with a test result)				Outcome: IgG index (divided by 10) N=212 (everybody with a test result)			
	Model 1		Model 2		Model 3		Model 4	
Predictors	OR (95% CI)	P	OR (95% CI)	P	Est (SE)	P	Est (SE)	P
Age (per decade)	0.8 (0.7, 0.91)	0.001	0.84 (0.73, 0.97)	0.016	0.02 (0.01)	0.07	0.02 (0.01)	0.046
Male Sex	1.19 (0.89, 1.59)	0.24			-0.05 (0.03)	0.11		
Hispanic Ethnicity	1.76 (1.28, 2.4)	<0.001	1.84 (1.31, 2.59)	0.001	0 (0.03)	0.93		
African American Race	1.77 (1.07, 2.93)	0.027	2.11 (1.24, 3.58)	0.006	0.02 (0.05)	0.66		
# people in home	1.02 (0.94, 1.11)	0.6			0.02 (0.01)	0.038	0.01 (0.01)	0.21
Physician Suspected Covid Diagnosis	10.14 (7.59, 13.55)	<0.001	7.78 (5.73, 10.56)	<0.001	0.16 (0.02)	<0.001	0.13 (0.03)	<0.001
Household Covid Diagnosis	18.93 (11.74, 30.53)	<0.001	9.42 (5.5, 16.13)	<0.001	0.09 (0.04)	0.016	0.03 (0.04)	0.47
Domestic Travel	0.61 (0.44, 0.84)	0.002	0.67 (0.48, 0.94)	0.021	-0.05 (0.03)	0.08	-0.04 (0.03)	0.18
International Travel	0.93 (0.66, 1.31)	0.68			0 (0.03)	0.98		
Covid Unit	1.98 (1.49, 2.63)	<0.001	1.61 (1.18, 2.18)	0.002	0.10 (0.03)	<0.001	0.07 (0.03)	0.01
Dwelling: House	1.2 (0.89, 1.61)	0.23			0.03 (0.03)	0.27		
Dwelling: Other	1.17 (0.58, 2.35)	0.67			0.05 (0.07)	0.44		
Persons <18 in home	0.96 (0.71, 1.29)	0.77			0.03 (0.03)	0.31		
Person <12 in home	0.91 (0.66, 1.26)	0.58			0.02 (0.03)	0.47		
Cats in home	0.98 (0.65, 1.48)	0.92			-0.01 (0.04)	0.87		
Dogs in home	1.34 (1.02, 1.78)	0.039	1.29 (0.95, 1.75)	0.10	0.01 (0.03)	0.78		

Model 1 is adjusted for age, sex, race, ethnicity.
Model 2 is adjusted for anything that was significant in Model 1 to a P<0.10.

Model 3 is adjusted age, sex
Model 4 is adjusted for anything that was significant in Model 3 to a P<0.10.

For peer review only - http://bmjopen.bmj.com/site/about/guidelines.xhtml

Supplemental Table 6. Potential COVID Illness Response Factors Associated with SARS-CoV-2 Seroprevalence

	Outcome: Antibody Positive N=6,062 (everybody with a test result)				Outcome: IgG index (divided by 10) N=212 (everybody with a test result)			
	Model 1		Model 2		Model 3		Model 4	
Predictors	OR (95% CI)	P	OR (95% CI)	P	Est (SE)	P	Est (SE)	P
Age (per decade)	0.8 (0.7, 0.91)	0.001	0.77 (0.65, 0.91)	0.002	0.02 (0.01)	0.07	0.02 (0.01)	0.05
Male Sex	1.19 (0.89, 1.59)	0.24			-0.05 (0.03)	0.11		
Hispanic Ethnicity	1.76 (1.29, 2.4)	<0.001	1.91 (1.3, 2.82)	0.001	0 (0.03)	0.93		
African American Race	1.77 (1.07, 2.93)	0.027	1.75 (0.92, 3.3)	0.09	0.02 (0.05)	0.66		
Fever	7.8 (5.81, 10.48)	<0.001	2.11 (1.26, 3.55)	0.005	0.15 (0.03)	<0.001	0.08 (0.04)	0.032
Chills	6.23 (4.67, 8.31)	<0.001	1.24 (0.73, 2.11)	0.44	0.11 (0.03)	<0.001	-0.04 (0.04)	0.31
Headache	2.72 (2.03, 3.64)	<0.001	0.69 (0.44, 1.09)	0.11	0.12 (0.03)	<0.001	0.07 (0.04)	0.06
Conjunctivitis	2.56 (1.45, 4.52)	0.001	0.95 (0.45, 2)	0.89	-0.04 (0.06)	0.5		
Anosmia	23.05 (16.98, 31.29)	<0.001	11.53 (7.51, 17.7)	<0.001	0.08 (0.03)	0.002	0 (0.03)	1
Nasal Congestion	2.59 (1.95, 3.44)	<0.001	1.18 (0.71, 1.97)	0.53	0.07 (0.03)	0.017	0.01 (0.03)	0.75
Rhinorrhea	1.89 (1.41, 2.52)	<0.001	0.6 (0.36, 1)	0.049	0.02 (0.03)	0.41		
Dry Cough	4.28 (3.21, 5.69)	<0.001	1.86 (1.21, 2.88)	0.005	0.09 (0.03)	0.001	-0.04 (0.04)	0.3
Productive Cough	3.01 (2.16, 4.2)	<0.001	0.82 (0.49, 1.36)	0.44	0.09 (0.03)	0.005	0.01 (0.04)	0.72
Sore Throat	2.09 (1.56, 2.8)	<0.001	0.47 (0.3, 0.74)	0.001	0.03 (0.03)	0.3		
Chest Pain	3.2 (2.26, 4.53)	<0.001	0.95 (0.56, 1.62)	0.85	0.07 (0.03)	0.034	-0.05 (0.04)	0.18
Dyspnea	4.08 (3, 5.56)	<0.001	0.88 (0.54, 1.44)	0.61	0.16 (0.03)	<0.001	0.13 (0.04)	0.001
Anorexia	8.57 (6.31, 11.63)	<0.001	2.19 (1.34, 3.57)	0.002	0.14 (0.03)	<0.001	0.04 (0.04)	0.27
Nausea	2.59 (1.86, 3.6)	<0.001	0.86 (0.51, 1.44)	0.56	0.1 (0.03)	0.002	0.08 (0.04)	0.05
Vomiting	2.33 (1.34, 4.03)	0.003	0.69 (0.31, 1.52)	0.36	0.15 (0.05)	0.005	-0.07 (0.06)	0.28
Diarrhea	2.32 (1.69, 3.18)	<0.001	0.83 (0.53, 1.31)	0.43	0.08 (0.03)	0.014	-0.04 (0.04)	0.25
Myalgias	6.36 (4.76, 8.5)	<0.001	1.92 (1.14, 3.25)	0.015	0.13 (0.03)	<0.001	0.04 (0.04)	0.33
Fatigue	5.91 (4.38, 7.98)	<0.001	1.63 (0.95, 2.77)	0.07	0.14 (0.03)	<0.001	0.01 (0.05)	0.76
Skin Changes	1.65 (0.96, 2.83)	0.07	0.89 (0.44, 1.81)	0.75	0.01 (0.05)	0.88		
Stroke Symptoms	2.35 (0.71, 7.78)	0.16			0.27 (0.11)	0.019	0.05 (0.13)	0.7
Sneezing	1.72 (1.29, 2.28)	<0.001	0.83 (0.52, 1.31)	0.42	0.03 (0.03)	0.36		

For peer review only - http://bmjopen.bmj.com/site/about/guidelines.xhtml

Model 1 is adjusted for age, sex, race, ethnicity.
Model 2 is adjusted for anything that was significant in Model 1 to a P<0.10.

Model 3 is adjusted for age, sex.
Model 4 is adjusted for anything that was significant in Model 3 to a P<0.10.

For peer review only

For peer review only - http://bmjopen.bmj.com/site/about/guidelines.xhtml

Supplemental Table 7. Factors Associated with SARS-CoV-2

Predictors	Outcome: Antibody Positive N=6,062 (everybody with a test result)		Outcome: IgG index (divided by 10) N=212 (everybody with a test result)	
	OR (95% CI)	P	Est (SE)	P
Age (per decade)	0.81 (0.69, 0.96)	0.017	0.02 (0.01)	0.22
Hispanic Ethnicity	2.11 (1.43, 3.13)	<0.001		
African American Race	2.08 (1.12, 3.88)	0.021		
Asthma	0.25 (0.13, 0.5)	<0.001		
Hypertension			0.09 (0.04)	0.031
Physician Suspected Covid Diagnosis	3.76 (2.52, 5.59)	<0.001	0.09 (0.03)	0.002
Household Covid Diagnosis	6.09 (3.08, 12.06)	<0.001		
Domestic Travel	0.63 (0.42, 0.92)	0.019		
Covid Unit	1.75 (1.23, 2.5)	0.002	0.07 (0.03)	0.008
Fever	1.94 (1.23, 3.07)	0.004	0.03 (0.03)	0.42
Headache			0.04 (0.03)	0.22
Anosmia	10.44 (6.78, 16.07)	<0.001		
Rhinorrhea	0.58 (0.38, 0.89)	0.012		
Dry Cough	1.2 (0.77, 1.86)	0.42		
Sore Throat	0.5 (0.32, 0.77)	0.002		
Dyspnea			0.07 (0.03)	0.015
Anorexia	1.52 (0.94, 2.46)	0.09		
Nausea			0.05 (0.03)	0.15
Myalgias	1.47 (0.88, 2.48)	0.14		
Fatigue	1.46 (0.87, 2.44)	0.15		

Models are adjusted for significant predictors from the primary multivariable models examining associations of existing characteristics, exposures and symptoms with antibody positivity and IgG titer index.

For peer review only - http://bmjopen.bmj.com/site/about/guidelines.xhtml

Supplemental Figure 1.

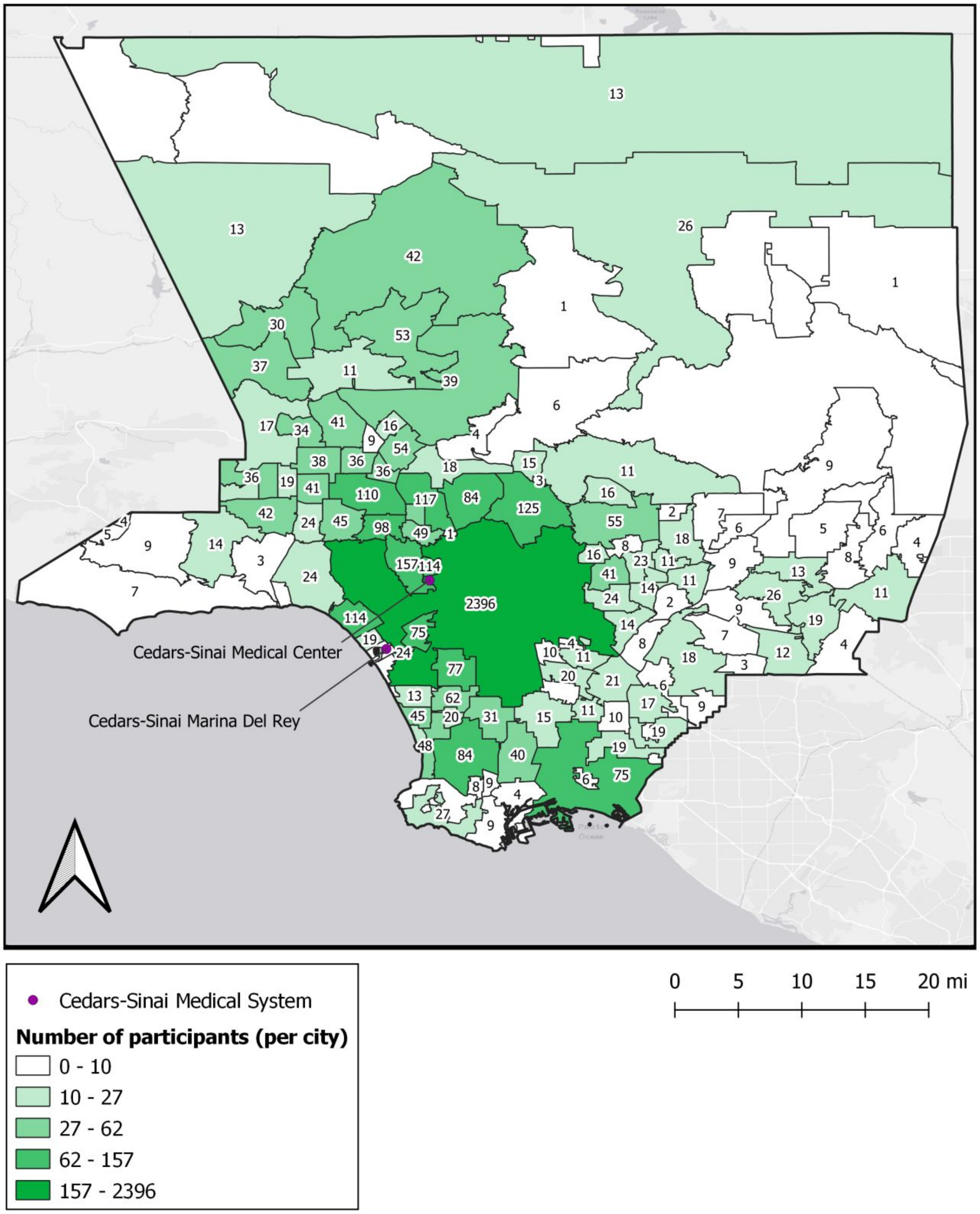

For peer review only - http://bmjopen.bmj.com/site/about/guidelines.xhtml

SUPPLEMENTAL REFERENCES

1. Abbott. ARCHITECT SARS-CoV-2 IgG Instructions for Use. 2020.
2. Bryan A, Pepper G, Wener MH, et al. Performance Characteristics of the Abbott Architect SARS-CoV-2 IgG Assay and Seroprevalence in Boise, Idaho. *J Clin Microbiol.* 2020.
3. Ng D, Goldgof G, Shy B, et al. SARS-CoV-2 seroprevalence and neutralizing activity in donor and patient blood from the San Francisco Bay Area. *medRxiv.* 2020:2020.2005.2019.20107482.
4. Ekelund O, Ekblom K, Somajo S, Pattison-Granberg J, Olsson K, Petersson A. High-throughput immunoassays for SARS-CoV-2, considerable differences in performance when comparing three methods. *medRxiv.* 2020:2020.2005.2022.20106294.
5. Phipps WS, SoRelle JA, Li Q-Z, et al. SARS-CoV-2 Antibody responses do not predict COVID-19 disease severity. *medRxiv.* 2020:2020.2005.2015.20103580.
6. Chew KL, Tan SS, Saw S, et al. Clinical evaluation of serological IgG antibody response on the Abbott Architect for established SARS-CoV-2 infection. *Clinical Microbiology and Infection.* 2020.
7. Theel ES, Harring J, Hilgart H, Granger D. Performance Characteristics of Four High-Throughput Immunoassays for Detection of IgG Antibodies against SARS-CoV-2. *Journal of Clinical Microbiology.* 2020:JCM.01243-01220.
8. Kohmer N, Westhaus S, Rühl C, Ciesek S, Rabenau HF. Brief clinical evaluation of six high-throughput SARS-CoV-2 IgG antibody assays. *Journal of Clinical Virology.* 2020;129:104480.
9. Stroemer A, Grobe O, Rose R, Fickenscher H, Lorentz T, Krumbholz A. Diagnostic accuracy of six commercial SARS-CoV-2 IgG/total antibody assays and identification of SARS-CoV-2 neutralizing antibodies in convalescent sera. *medRxiv.* 2020:2020.2006.2015.20131672.

For peer review only

For peer review only - http://bmjopen.bmj.com/site/about/guidelines.xhtml

10. Nicol T, Lefeuvre C, Serri O, et al. Assessment of SARS-CoV-2 serological tests for the diagnosis of COVID-19 through the evaluation of three immunoassays: Two automated immunoassays (Euroimmun and Abbott) and one rapid lateral flow immunoassay (NG Biotech). *Journal of Clinical Virology.* 2020;129:104511.
11. Dellière S, Salmona M, Minier M, et al. Evaluation of COVID-19 IgG/IgM Rapid Test from Orient Gene Biotech. *Journal of Clinical Microbiology.* 2020:JCM.01233-01220.
12. Perkmann T, Perkmann-Nagele N, Breyer M-K, et al. Side by side comparison of three fully automated SARS-CoV-2 antibody assays with a focus on specificity. *medRxiv.* 2020:2020.2006.2004.20117911.
13. Mueller L, Ostermann PN, Walker A, et al. Sensitivity of commercial Anti-SARS-CoV-2 serological assays in a high-prevalence setting. *medRxiv.* 2020:2020.2006.2011.20128686.
14. Tang MS, Hock KG, Logsdon NM, et al. Clinical Performance of Two SARS-CoV-2 Serologic Assays. *Clinical Chemistry.* 2020.
15. Guo W, Li M, Dong Y, et al. Diabetes is a risk factor for the progression and prognosis of COVID-19. *Diabetes/metabolism research and reviews.* 2020:e3319.
16. Jääskeläinen AJ, Kuivanen S, Kekäläinen E, et al. Performance of six SARS-CoV-2 immunoassays in comparison with microneutralisation. *medRxiv.* 2020:2020.2005.2018.20101618.
17. Paiva KJ, Grisson RD, Chan PA, et al. Validation and Performance Comparison of Three SARS-CoV-2 Antibody Assays. *bioRxiv.* 2020:2020.2005.2029.124776.
18. Brecher SM, Dryjowicz-Burek J, Yu H, Campbell S, Ratcliffe N, Gupta K. Patients with Common Cold Coronaviruses Tested Negative for IgG Antibody to SARS-CoV-2. *Journal of Clinical Microbiology.* 2020:JCM.01029-01020.

For peer review only

For peer review only - http://bmjopen.bmj.com/site/about/guidelines.xhtml

STROBE 2007 (v4) Statement—Checklist of items that should be included in reports of *cross-sectional studies*

Section/Topic	Item #	Recommendation	Reported on page #
**Title and abstract**	1	(*a*) Indicate the study's design with a commonly used term in the title or the abstract	Pg.3
		(*b*) Provide in the abstract an informative and balanced summary of what was done and what was found	Pg. 3-4
**Introduction**			
Background/rationale	2	Explain the scientific background and rationale for the investigation being reported	Pg.5
Objectives	3	State specific objectives, including any prespecified hypotheses	Pg.5
**Methods**			
Study design	4	Present key elements of study design early in the paper	Pg.6
Setting	5	Describe the setting, locations, and relevant dates, including periods of recruitment, exposure, follow-up, and data collection	Pg.6
Participants	6	(*a*) Give the eligibility criteria, and the sources and methods of selection of participants	Pg.6
Variables	7	Clearly define all outcomes, exposures, predictors, potential confounders, and effect modifiers. Give diagnostic criteria, if applicable	Pg.7-8
Data sources/ measurement	8*	For each variable of interest, give sources of data and details of methods of assessment (measurement). Describe comparability of assessment methods if there is more than one group	Pg.6-8
Bias	9	Describe any efforts to address potential sources of bias	Pg.6-7
Study size	10	Explain how the study size was arrived at	Pg.6
Quantitative variables	11	Explain how quantitative variables were handled in the analyses. If applicable, describe which groupings were chosen and why	Pg.7
Statistical methods	12	(*a*) Describe all statistical methods, including those used to control for confounding	Pg.7-8
		(*b*) Describe any methods used to examine subgroups and interactions	Pg.7-8
		(*c*) Explain how missing data were addressed	Pg.7-8
		(*d*) If applicable, describe analytical methods taking account of sampling strategy	Pg.7-8
		(*e*) Describe any sensitivity analyses	Pg.7-8
**Results**			

For peer review only

For peer review only - http://bmjopen.bmj.com/site/about/guidelines.xhtml

Participants	13*	(a) Report numbers of individuals at each stage of study—eg numbers potentially eligible, examined for eligibility, confirmed eligible, included in the study, completing follow-up, and analysed	Pg.9
		(b) Give reasons for non-participation at each stage	Pg.6
		(c) Consider use of a flow diagram	N/A
Descriptive data	14*	(a) Give characteristics of study participants (eg demographic, clinical, social) and information on exposures and potential confounders	Pg. 9
		(b) Indicate number of participants with missing data for each variable of interest	N/A
Outcome data	15*	Report numbers of outcome events or summary measures	Pg. 9-10
Main results	16	(*a*) Give unadjusted estimates and, if applicable, confounder-adjusted estimates and their precision (eg, 95% confidence interval). Make clear which confounders were adjusted for and why they were included	Pg.9-10
		(*b*) Report category boundaries when continuous variables were categorized	Pg.9-10
		(*c*) If relevant, consider translating estimates of relative risk into absolute risk for a meaningful time period	NA
Other analyses	17	Report other analyses done—eg analyses of subgroups and interactions, and sensitivity analyses	Pg.7-8
**Discussion**			
Key results	18	Summarise key results with reference to study objectives	Pg. 11-14
Limitations	19	Discuss limitations of the study, taking into account sources of potential bias or imprecision. Discuss both direction and magnitude of any potential bias	Pg. 14
Interpretation	20	Give a cautious overall interpretation of results considering objectives, limitations, multiplicity of analyses, results from similar studies, and other relevant evidence	Pg. 14
Generalisability	21	Discuss the generalisability (external validity) of the study results	Pg. 13-14
**Other information**			
Funding	22	Give the source of funding and the role of the funders for the present study and, if applicable, for the original study on which the present article is based	Pg. 15

*Give information separately for cases and controls in case-control studies and, if applicable, for exposed and unexposed groups in cohort and cross-sectional studies.

Note: An Explanation and Elaboration article discusses each checklist item and gives methodological background and published examples of transparent reporting. The STROBE checklist is best used in conjunction with this article (freely available on the Web sites of PLoS Medicine at http://www.plosmedicine.org/, Annals of Internal Medicine at http://www.annals.org/, and Epidemiology at http://www.epidem.com/). Information on the STROBE Initiative is available at www.strobe-statement.org.

For peer review only

BMJ Open

Seroprevalence of Antibodies to SARS-CoV-2 in Healthcare Workers: A Cross-Sectional Study

Journal:	*BMJ Open*
Manuscript ID	bmjopen-2020-043584.R1
Article Type:	Original research
Date Submitted by the Author:	30-Nov-2020

Complete List of Authors:	Ebinger, Joseph ; Cedars-Sinai Medical Center<br>Botwin, Gregory ; Cedars-Sinai Medical Center<br>Albert, Christine; Cedars-Sinai Medical Center<br>Alotaibi, Mona; University of California San Diego, Department of Pulmonology<br>Arditi, Moshe; Cedars-Sinai Medical Center, Division of Pediatric Infectious Diseases<br>Berg, Anders; Cedars-Sinai Medical Center<br>Binek , Aleksandra; Cedars-Sinai Medical Center<br>Botting, Patrick; Cedars-Sinai Medical Center<br>Fert-Bober, Justyna; Cedars-Sinai Medical Center<br>Figueiredo, Jane; Cedars-Sinai Medical Center<br>Grein, Jonathan; Cedars-Sinai Medical Center<br>Hasan, Wohaib; Cedars-Sinai Medical Center<br>Henglin, Mir; Cedars-Sinai Medical Center<br>Hussain, Shehnaz; Cedars-Sinai Medical Center<br>Jain, Mohit; University of California San Diego, Department of Medicine and Pharmacology<br>Joung, Sandy; Cedars-Sinai Medical Center<br>Karin, Michael; University of California San Diego School of Medicine<br>Kim, Elizabeth; Cedars-Sinai Medical Center<br>Li, Dalin; Cedars-Sinai Medical Center<br>Liu, Yunxian; Cedars-Sinai Medical Center<br>Luong, Eric; Cedars-Sinai Medical Center<br>McGovern, Dermot; Cedars-Sinai Medical Center<br>Merchant, Akil; Cedars-Sinai Medical Center<br>Merin, Noah; Cedars-Sinai Medical Center<br>Miles, Peggy; Cedars-Sinai Medical Center<br>Minissian , Margo; Cedars-Sinai Medical Center<br>Nguyen , Trevor Trung; Cedars-Sinai Medical Center<br>Raedschelders , Koen; Cedars-Sinai Medical Center<br>Rashid, Mohamad; Cedars-Sinai Medical Center<br>Riera, Celine ; Cedars-Sinai Medical Center<br>Riggs, Richard; Cedars-Sinai Medical Center<br>Sharma , Sonia; La Jolla Institute for Allergy and Immunology<br>Sternbach, Sarah; Cedars-Sinai Medical Center<br>Sun, Nancy; Cedars-Sinai Medical Center<br>Tourtellotte, Warren; Cedars-Sinai Medical Center<br>Van Eyk, Jennifer; Cedars-Sinai Medical Center<br>Sobhani , Kimia; Cedars-Sinai Medical Center<br>Braun, Jonathan; Cedars-Sinai Medical Center

	Cheng, Susan; Cedars-Sinai Medical Center, Cardiology
**Primary Subject Heading**:	Public health
Secondary Subject Heading:	Epidemiology
Keywords:	COVID-19, INFECTIOUS DISEASES, Public health < INFECTIOUS DISEASES

SCHOLARONE™
Manuscripts

I, the Submitting Author has the right to grant and does grant on behalf of all authors of the Work (as defined in the below author licence), an exclusive licence and/or a non-exclusive licence for contributions from authors who are: i) UK Crown employees; ii) where BMJ has agreed a CC-BY licence shall apply, and/or iii) in accordance with the terms applicable for US Federal Government officers or employees acting as part of their official duties; on a worldwide, perpetual, irrevocable, royalty-free basis to BMJ Publishing Group Ltd ("BMJ") its licensees and where the relevant Journal is co-owned by BMJ to the co-owners of the Journal, to publish the Work in this journal and any other BMJ products and to exploit all rights, as set out in our licence.

The Submitting Author accepts and understands that any supply made under these terms is made by BMJ to the Submitting Author unless you are acting as an employee on behalf of your employer or a postgraduate student of an affiliated institution which is paying any applicable article publishing charge ("APC") for Open Access articles. Where the Submitting Author wishes to make the Work available on an Open Access basis (and intends to pay the relevant APC), the terms of reuse of such Open Access shall be governed by a Creative Commons licence – details of these licences and which Creative Commons licence will apply to this Work are set out in our licence referred to above.

Other than as permitted in any relevant BMJ Author's Self Archiving Policies, I confirm this Work has not been accepted for publication elsewhere, is not being considered for publication elsewhere and does not duplicate material already published. I confirm all authors consent to publication of this Work and authorise the granting of this licence.

For peer review only

Seroprevalence of Antibodies to SARS-CoV-2 in Healthcare Workers: A Cross-Sectional Study

Joseph E. Ebinger, MD, MS,[1,2]* Gregory J. Botwin, BS,[3]* Christine M. Albert, MD, MPH,[1,2] Mona Alotaibi,MD,[4] Moshe Arditi, MD,[2,5,6] Anders H. Berg, MD, PhD,[7] Aleksandra Binek, PhD,[8] Patrick Botting, MSPH,[1,2] Justyna Fert-Bober, Phd,[2] Jane C. Figueiredo, PhD[9] Jonathan D. Grein, MD,[10,11] Wohaib Hasan, PhD,[7,12] Mir Henglin, BA,[1,2] Shehnaz K. Hussain, PhD,[9] Mohit Jain, MD, PhD,[13] Sandy Joung, MHDS,[1,2] Michael Karin, PhD,[14] Elizabeth H. Kim, MHDS,[1,2] Dalin Li, PhD,[3] Yunxian Liu, PhD,[1,2] Eric Luong, MPH,[1,2] Dermot P.B. McGovern, MD, PhD,[3] Akil Merchant, MD,[10] Noah Merin, MD, PhD,[15] Peggy B. Miles, MD,[16] Margo Minissian, PhD,[1,2,17] Trevor-Trung Nguyen, BS,[1,2] Koen Raedschelders, PhD,[1,2,8] Mohamad A. Rashid, MBChB,[1,2] Celine E. Riera, PhD,[18,19] Richard V. Riggs, MD,[20] Sonia Sharma, PhD[21] Sarah Sternbach,BS,[2] Nancy Sun, MPS,[1,2] Warren G. Tourtellotte, MD, PhD,[7,12] Jennifer E. Van Eyk, PhD,[1,8,22] Kimia Sobhani, PhD,[7]* Jonathan G. Braun, MD, PhD,[7]* Susan Cheng, MD, MPH[1,2,22]*

From [1]Department of Cardiology, Cedars-Sinai Medical Center, Los Angeles, California, USA; [2]Smidt Heart Institute, Cedars-Sinai Medical Center, Los Angeles, California, USA; [3]F. Widjaja Foundation Inflammatory Bowel and Immunobiology Research Institute, Cedars-Sinai Medical Center, Los Angeles, California, USA; [4]Division of Pulmonary and Critical Care Medicine, University of California, San Diego, San Diego, California, USA; [5]Departments of Pediatrics, Division of Infectious Diseases and Immunology, and Infectious and Immunologic Diseases Research Center (IIDRC), Department of Biomedical Sciences, Cedars-Sinai Medical Center, Los Angeles, California, USA; [6]Department of Pediatrics, David Geffen School of Medicine at UCLA, Los Angeles, California, USA; [7]Department of Pathology and Laboratory Medicine, Cedars-Sinai Medical Center, Los Angeles, California, USA; [8]Advanced Clinical Biosystems Institute, Department of Biomedical Sciences, Cedars-Sinai Medical Center, Los Angeles, California, USA; [9]Cedars-Sinai Cancer and Department of Medicine, Cedars-Sinai Medical

For peer review only - http://bmjopen.bmj.com/site/about/guidelines.xhtml

Center, Los Angeles, California, USA; [10]Department of Medicine, Cedars-Sinai Medical Center, Los Angeles, California, USA; [11]Department of Epidemiology, Cedars-Sinai Medical Center, Los Angeles, California, USA; [12]Biobank & Translational Research Core Laboratory, Samuel Oschin Comprehensive Cancer Institute, Cedars-Sinai Medical Center, Los Angeles, California, USA; [13]Department of Medicine, School of Medicine, University of California, San Diego, San Diego, CA; [14]Department of Pharmacology, University of California, San Diego School of Medicine, San Diego, California, USA; [15]Department of Internal Medicine, Division of Hematology Cedars-Sinai Medical Center, Los Angeles, California, USA; [16]Employee Health Services, Department of Medicine, Cedars-Sinai Medical Center, Los Angeles, California, USA; [17]Brawerman Nursing Institute, Cedars-Sinai Medical Center, Los Angeles, California, USA; [18]Center for Neural Science and Medicine, Department of Biomedical Sciences, Board of Governors Regenerative Medicine Institute, Department of Neurology, Cedars-Sinai Medical Center, Los Angeles, California, USA; [19]David Geffen School of Medicine, University of California, Los Angeles, Los Angeles, California, USA; [20]Chief Medical Officer, Cedars-Sinai Medical Center, Los Angeles, California, USA; [21]La Jolla Institute for Allergy and Immunology, La Jolla, California,USA;[22]Barbra Streisand Women's Heart Center, Cedars-Sinai Medical Center, Los Angeles, California, USA.

Correspondence: Kimia Sobhani, PhD, Department of Pathology and Laboratory Medicine, Cedars-Sinai Medical Center, Los Angeles, CA; phone (310) 423-5405; email kimia.sobhani@cshs.org; Jonathan G. Braun, MD, PhD, F. Widjaja Foundation Inflammatory Bowel and Immunobiology Research Institute, Cedars Sinai Medical Center, Los Angeles, CA; phone (310) 423-8717; email jonathan.braun2@cshs.org; Susan Cheng, MD, MPH, Department of Cardiology, Smidt Heart Institute, Cedars Sinai Medical Center, Los Angeles, CA; phone (310) 423-2726; email susan.cheng@cshs.org.

only

Wordcount: 3,027

For peer review only - http://bmjopen.bmj.com/site/about/guidelines.xhtml

Key Words: COVID-19; Antibodies; Anosmia; Disparities; Healthcare workers

For peer review only

For peer review only - http://bmjopen.bmj.com/site/about/guidelines.xhtml

ABSTRACT (300 word limit)

Objective: We sought to determine the extent of severe acute respiratory syndrome coronavirus 2 (SARS-CoV-2) seroprevalance and the factors associated with seroprevelance across a diverse cohort of healthcare workers.

Design: Observational cohort study of healthcare workers, including SARS-CoV-2 serology testing and participant questionaires.

Settings: A multi-site healthcare delivery system located in Los Angeles County.

Participants: A diverse and unselected population of adults (n=6,062) employed in a multi-site healthcare delivery system located in Los Angeles County, including individuals with direct patient contact and others with non-patient-oriented work functions.

Main Outcomes: Using Bayesian and multi-variate analyses, we estimated seroprevalence and factors associated with seropositivity and antibody levels, including pre-existing demographic and clinical characteristics; potential coronavirus disease 2019 (COVID-19) illness related exposures; and, symptoms consistent with COVID-19 infection.

Results: We observed a seroprevalence rate of 4.1%, with anosmia as the most prominently associated self-reported symptom (OR 11.04, P<0.001) in addition to fever (OR 2.02, P=0.002) and myalgias (OR 1.65, P=0.035). After adjusting for potential confounders, seroprevalence was also associated with Hispanic ethnicity (OR 1.98, P=0.001) and African-American race (OR 2.02, P=0.027) as well as contact with a COVID-19 diagnosed individual in the household (OR 5.73, P<0.001) or clinical work setting (OR 1.76, P=0.002). Importantly, African American race and Hispanic ethnicity were associated with antibody positivity even after adjusting for personal COVID-19 diagnosis status, suggesting the contribution of unmeasured structural or societally factors.

For peer review only - http://bmjopen.bmj.com/site/about/guidelines.xhtml
For peer review only

Conclusion and Relevance: The demographic factors associated with SARS-CoV-2 seroprevalence among our healthcare workers underscore the importance of exposure sources beyond the workplace. The size and diversity of our study population, combined with robust survey and modeling techniques, provide a vibrant picture of the demographic factors, exposures, and symptoms that can identify individuals with susceptibility as well as potential to mount an immune response to COVID-19.

For peer review only

STRENGTHS AND LIMITATIONS

- Our study was strengthened by the size and granularity of data available on participants.
- Our broad definition of healthcare worker, including patient facing and non-patient facing employees, enhanced diversity of the study and generalizability of the results.
- Data collected on medical history, exposures, and symptoms were self-reported.
- Variations in the timing of prior symptom onset in relation to the immunoassay likely resulted in underestimation of seroprevalence.
- Additional data on the specific roles and nature of clinical care performed by healthcare workers, including roles involving nasopharygeal or respiratory procedures, are needed for future investigations.

For peer review only - http://bmjopen.bmj.com/site/about/guidelines.xhtml

INTRODUCTION

Amidst the ongoing global pandemic caused by severe acute respiratory syndrome coronavirus 2 (SARS-CoV-2), the viral agent causing coronavirus disease 2019 (COVID-19), substantial attention[1] turned to antibody testing as an approach to understanding patterns of exposure and immunity across populations. The use and interpretion of antibody testing to assess exposure and immunity remains frought with inconsistencies and unclear clinical correlations, in part due to a dearth of high quality studies among diverse participants.[2,3] Recent publications have pointed to the challenges and importance of understanding how different antibody tests for SARS-CoV-2 perform, and factors that may render one method superior to another.[4,5] Nonetheless, there remains general agreement that antibody testing offers valuable information regarding the probable extent of SARS-CoV-2 exposure, the factors associated with exposure, and the potential nature and determinants of seropositive status.[6]

To that end, we conducted a study of SARS-CoV-2 antibody screening of a large, diverse, and unselected population of adults employed in a multi-site healthcare delivery system located in Los Angeles County, including individuals with direct patient contact and others with non-patient-oriented work functions. Recognizing the range of factors that might influence antibody status in a given individual, we focused our study on not only estimating seroprevalence but also on identifying factors associated with seropositivity and relative antibody levels within the following three categories: (1) pre-existing demographic and clinical characteristics; (2) potential COVID-19 illness related exposures; and, (3) COVID-19 illness related response variables (i.e. different types of self-reported symptoms).

For peer review only - http://bmjopen.bmj.com/site/about/guidelines.xhtml

METHODS

Study Sample

The sampling strategy for our study has been described previously.[7] In brief, beginning on May 11, 2020, we enrolled a total of N=6,318 active employees working at multiple sites comprising the Cedars-Sinai Health System, located in the diverse metropolis of Los Angeles County, California. The Cedars-Sinai organization includes two hospitals (Cedars-Sinai Medical Center and Marina Del Rey Hospital) in addition to multiple clinics in the Cedars-Sinai Medical Delivery Network. All active employees (total N~15,000) were invited to participate in the study by providing a peripheral venous blood sample for serology testing and completing an electronic survey of questions regarding past medical history, social history, and work environment in addition to COVID-19 related symptoms and exposures.[8,9] For the current study, we included all participants who completed both SARS-CoV-2 antibody testing and electronic survey forms (N=6,062). Survey forms collected data on pre-existing traits, exposure factors including work location, and previously experienced symptoms. Work location was specified as spending most working hours in an ICU (COVID-19 or non-COVID-19 designated), non-ICU ward (COVID-19 or non-COVID-19 designated), outpatient clinic, office, work-from-home, or other location. The study protocol was approved by the Cedars-Sinai institutional review board and all participants provided written informed consent.

Serologic Assays

For all participants, EDTA plasma specimens were transported within 1 hour of phlebotomy to the Cedars-Sinai Department of Pathology and Laboratory Medicine and underwent serology testing using the Abbott Diagnostics SARS-CoV-2 IgG chemiluminescent microparticle immunoassay (Abbott Diagnostics, Abbott Park, IL) performed on an Abbott Diagnostics Architect ci16200 analyzer. The assay reports a signal-to-cutoff ratio (S/CO) corresponding to the relative light units

For peer review only - http://bmjopen.bmj.com/site/about/guidelines.xhtml

produced by the test sample compared to the relative light units produced by an assay calibrator sample. The manufacturer recommended S/CO ratio of 1.4 was used to assign binary seropositivity status. This cutoff was validated for high specificity (i.e., >99%) ~14 days post symptom onset.[10] The Abbott assay detects antibodies directed against the nucleocapsid (N) antigen of the SARS-CoV-2 virus, which assists with packaging the viral genome after replication, and achieves specificity for IgG by incorporating an anti-human IgG signal antibody. To verify local performance of the assay, we used samples obtained at our institution from 60 cases of COVID-19 (hospitalized between March and May 2020) and 178 controls that were identified based on positive or negative PCR assay (RT-qPCR assay based on A*STAR Fortitude Kit 2.0) with a time lapse between symptom onset and antibody assay of ~7 to 14 days. We found a sensitivity or positive percent agreement (PPA) of 88.3%, with CVs of ≤1.4% for positive and negative controls.

Statistical Analyses

Estimates of Seroprevalence. We conducted a comprehensive literature review to identify published data (through June 25, 2020) on the sensitivity and specificity of the Abbott Architect SARS-CoV-2 IgG assay, as applied in specific populations using the manufacturer's recommended thresholds. We identified a total of 15 studies assessing sensitivity in 2,114 tests and 18 studies reporting specificity in 7,748 tests (**Supplemental Tables 1-2**); we combined this information with data from an additional independent cohort of 60 case and 178 control specimens used to asses sensitivity and specificity, respectively, within the Cedars-Sinai Department of Pathology and Laboratory Medicine. We noted that studies investigating specificity generally assessed samples collected prior to the SARS-CoV-2 pandemic whereas studies reporting sensitivity included specimens from RT-PCR confirmed individuals (see details provided in **Supplemental Tables 1-2**). We restricted our analyses to a referent cohort of tests conducted on samples from individuals who were assayed ≥7 days following symptoms onset to most closely

For peer review only - http://bmjopen.bmj.com/site/about/guidelines.xhtml

match our cohort sample characteristics and the situational context for study enrollment. Given that our study cohort included a large number, yet not the total number, of all eligible healthcare workers employed in our health system, we used the iterative proportional fitting (IPF) procedure to account for any possible sampling bias; notably, the IPF has been applied effectively in prior as well as contemporary studies related to SARS-CoV-2 exposure.[11] Accordingly, we integrated source population-level demographic data, representative of the entire Cedars-Sinai employee base, with data from our enrolled study sample and then used IPF to estimate the number of eligible employees within each demographic category (with provided population totals considered the target, using constraints derived from our sample).[12] In addition to accounting for potential bias from sampling, we also recognized the need to account for potential bias related to the previously reported sensitivity and specificity of the antibody assay (**Supplemental Tables 1-2**). Thus, in accordance with methods applied in similar seroprevalence studies,[13,14] we fit a Bayesian multilevel hierarchical logistic regression model using RStan,[15,16] including reported age, gender, race/ethnicity and site as coefficients, to model exposure probability. We then estimated the seroprevalence within each post-stratified demographic category based on the averaged and weighted value of the expected number of employees within that category.

Factors Associated with Seroprevalence. Prior to logistic and linear multivariable-adjusted analyses, age and IgG index were transformed by dividing by 10 for interpretability of coefficients in all models. In adjusted analyses, we compared differences between serology status (i.e. antibody positive versus negative) in each variable of interest, grouped into one of three categories: (1) pre-existing demographic and clinical characteristics (e.g. age, gender, ethnicity, race, and self-reported medical comorbidities); (2) COVID-19 related exposures (e.g. self-reported medical diagnosis of COVID-19 illness, household member with COVID-19 illness, number of people living in the home including children, type of home dwelling, etc); and, (3) COVID-19 related response variables (e.g. self-reported fever, chills, dry cough, anosmia,

For peer review only - http://bmjopen.bmj.com/site/about/guidelines.xhtml

nausea, myalgias, etc.). In multivariable-adjusted analyses, we used logistic and linear models to examine the extent to which the three categories of variables (predictors) may be associated with antibody positive status (primary outcome) in the total sample or IgG antibody level in the subset of persons with positive antibody status (secondary outcome). Initial models were deliberately sparse, adjusting for a limited number of key covariates (e.g. age, gender) and those variables with associations meeting a significance threshold of P<0.05 were advanced for inclusion in a final multivariable model along with only other variables identified as significant from the sparse regressions. A final separate logistic or linear multivariable model was constructed for each of the 3 categories of variables in relation to the binary outcome of seropositivity or the continuous outcome of IgG antibody level, respectively.

Patient and Public Involvement. Patients and the public were not involved in the development of this study.

For peer review only

For peer review only - http://bmjopen.bmj.com/site/about/guidelines.xhtml

RESULTS

The demographic, clinical, exposure, and symptom response characteristics of the study sample are shown in **Table 1**, by antibody test result status; the study sample included individuals whose residence spanned diverse regions across Los Angeles County (**Supplemental Figure 1**). The overall seroprevalence was 4.1% (95% CI 3.1%, 5.7%), with higher estimates seen in younger compared to older individuals and in Hispanics compared to non-Hispanics (**Figure 1** and **Supplemental Table 3**).

In multivariable-adjusted analyses of pre-existing characteristics (**Figure 2** and **Supplemental Table 4**), the main factors significantly associated with greater odds of seropositive status were Hispanic ethnicity (OR 1.80 [95% CI 1.31, 2.46], P<0.001), and African American race (1.72 [1.03, 2.89], P=0.04), compared to non-Hispanic Whites. The main factors associated with lower odds of being seropositive were older age (0.81 [0.71, 0.92] per age decade, P=0.001), and a history of asthma (0.48 [0.28, 0.83], P=0.009). Among all seropositive persons, hypertension was significantly associated with higher antibody level (beta 0.12 [SE 0.04] per 10-unit increment in the IgG index, P=0.003).

In multivariable-adjusted analyses of COVID-19 related exposures (**Figure 3** and **Supplemental Table 5**), the factors significantly associated with greater odds of seropositive status were having had a medical diagnosis of COVID-19 (7.78 [5.73, 10.56], P<0.001) and a household member previously diagnosed with COVID-19 (9.42 [5.50, 16.13], P<0.001), with a similar trend observed for working in a location where COVID-19 patients are treated (1.61 [1.18, 2.18], P=0.002]. Among seropositive individuals, having a medical diagnosis of COVID-19 was associated with higher antibody level. Notably, domestic travel, dwelling type, number of people in the home, and having children or common domestic pets were not associated with either seroprevalence or antibody

For peer review only - http://bmjopen.bmj.com/site/about/guidelines.xhtml

level in the more completely adjusted multivariable models, which can account at least partially for the effects unmeasured confounders that are not captured in the sparser models.

In multivariable-adjusted analyses of COVID-19 response variables (**Figure 4** and **Supplemental Table 6**), the strongest self-reported symptom associated with greater odds of seropositive status was anosmia (11.91 [7.77, 18.24], P<0.001). Other symptoms associated with the presence of antibodies included dry cough, loss of appetite, and myalgias. Notably, the symptoms associated with lower odds of seropositive status included sore throat and rhinorrhea. Dyspnea was significantly associated with higher IgG index levels in seropositive individuals (beta 0.13 [SE 0.04], P=0.001).

Significantly predictive pre-existing characteristics, exposures and symptoms from the prior models were subsequently analyzed together. In multivariable analysis, all included predictors, except for dry cough remained significantly associated with the presence of antibodies. Predictors which remained significantly associated with higher antibody levels included hypertension (beta 0.1 [SE 0.04], P=0.007), prior COVID-19 diagnosis (beta 0.1 [SE 0.03], P=0.001), working in a Covid unit (beta 0.06 [SE 0.03], P=0.021), dyspnea (beta 0.08 [SE 0.03], P=0.009), and nausea (beta 0.06 [SE 0.03], P=0.05. (**Figure 5** and **Supplemental Table 7**).

For peer review only - http://bmjopen.bmj.com/site/about/guidelines.xhtml

DISCUSSION

In a large diverse healthcare employee cohort of over 6,000 adults in Los Angeles, we observed a seroprevalence rate of 4.1%, which when accounting for published test characteristics, may range from 3.1% to 5.7%. Seroprevalence varied across demographic, clinical, exposure and symptom based characteristics. Specifically, factors significantly associated with presence of IgG antibodies included younger age, Hispanic ethnicity, and African-American race, as were exposure related factors including the presence of either a personal or household member having a prior medical diagnosis of COVID-19. Among self-reported symptoms, anosmia was most strongly associated with the presence of antibodies, with positive associations also noted for fever, dry cough, anorexia, and myalgias. The size and diversity of this study population, combined with robust survey and modeling techniques, provide a more vibrant picture of the population at highest risk for COVID-19 infection, risks of various potential exposures and symptoms that should alter patients to potential illness.

Most prior seroprevalence studies have focused on cohorts that included healthcare workers predominantly involved in direct or indirect patient care, persons living within a circumscribed region with high viral exposure rates, or larger geographic areas from which motivated individuals could voluntarily enroll into community screening programs.[17,18] Given that completely unbiased population-scale sampling for seroprevalence studies remains a logistical challenge, we used a sampling approach that involved open enrollment and convenient access to testing facilities made available to all employees working across multiple sites of a large healthcare system; this approach was intended to broadly capture individuals with both patient-related exposures and community-related exposures, while also representative of a relatively wide geographic area in and around Los Angeles County. Although limited to persons who are generally healthy and able to be employed, our study cohort included individuals representing a diversity of demographic

For peer review only - http://bmjopen.bmj.com/site/about/guidelines.xhtml

characteristics including ethnicity and race – leading to findings that reflect the disparities that have been persistently observed and reported for COVID-19 infection rates in our local communities. Similar to prior seroprevalence studies conducted across large samples sizes in other regions,[19] results from immunoassays performed at a single timepoint are likely to underestimate the true prior exposure and infection rate particularly given that SARS-CoV-2 IgG antibody levels are known to wane over a period of weeks to months.[20] Notwithstanding underestimated prior infection rates, related also to variable sensitivity of most IgG immunoassays in relation to timing of symptoms (ranging from ≥7 days to 6 months in our study), the overall seroprevalence that we observed is consistent with that reported for regionally proximate populations evaluated during a relatively contemporaneous time period.[21]

Consistent with findings from studies in healthcare workers, seroprevalence patterns in our cohort indicate exposure from not only the work environment but also from the home environment and likely unmeasured community-based factors.[22] It has been well reported that minority populations, particularly African Americans and Hispanics, have been disproportionately effected by the COVID-19 panedmic.[23-25] Our study is consistent with these prior findings, but demonstrates that such differences exist even when all participants work not just in the same field, but for the same organization. Such a finding may indicate that community and non-work related environmental factors are likely playing a significant role in the spread of COVID-19 among certain minority populations. Even after controlling for a medical diagnosis of COVID-19, African American race and Hispanic ethnicity remained risk factors for antibody positivity. The persistence of thse racial and ethnic disparities may represent structural barriers to care or societally mediated risk. Geographic clustering by race and ethnicity in housing, shopping and social gatherings may be one such factor, while socioeconomic status and ability to self-isolate outside of work likely also contribute.[26-28]

For peer review only - http://bmjopen.bmj.com/site/about/guidelines.xhtml

No self-reported pre-existing medical conditions were significantly associated with antibody positivity, indicating that infection itself is agnostic to baseline health. In fact, asthma was negatively associated with the presence of antibodies, or at least antibody levels above the current threshold we use for positivity. While reactive airway disease is unlikely a protective factor against COVID-19, participants with such conditions may be more likely to deligently follow social distancing guidelines and practice better adherence to hand hygiene and use of personal protective equipment. Hypertension was the only medical condition associated with higher SARS-CoV-2 antibody levels. It remains unclear as to what physiologic mechanism may contribute to this finding, however, unmeasured confounding variables, such as medications or renal disease may function as mediating factors. Further studies will be needed to both verify and elucidate this finding.

Also concordant with prior studies, we found that anosmia was the single strongest symptom associated with SARS-CoV-2 IgG antibody presence.[29-31] Anosmia is recognized as not only highly specific among the symptoms attributable to COVID-19 but is also known to be a particularly frequent finding among younger compared to older infected persons – which likely accounts in part for its especially prominent association with the ability to mount an immune response reflected by degree of detectable seropositivity. Interestingly, neither dyspnea nor diarrhea, two commonly cited symptoms, demonstrated a significant association in multivariable analysis.[32,33] This is likely related to the non-specific nature of these symptoms, which are common to multiple viral and non-viral etiologies. Importantly, dyspnea was associated with a higher antibody level among those with anti-SARS-CoV-2 antibodies, suggesting that dyspnea related to COVID-19 may drive a more robust humoral immune response, potentially related to more severe infection. These findings are concordant with the known phenomenon of proportionate adaptive immune response to higher doses of antigenic stress.[34] The extent to which the generation of measurably higher antibody levels could confer immunity to a larger

For peer review only - http://bmjopen.bmj.com/site/about/guidelines.xhtml

degree or for a longer duration of time remains unknown. Interestingly, prior studies have demonstrated lower antibody levels among exposed, asymptomatic individuals, a phenomena which may be attributable to a highly efficient cell mediated immune response.[35] It has be suggested that higher T-cell levels, whether virus specific or otherwise, may play a role in this finding, however, further research is required.[36,37]

Several limitations of this study merit consideration. Of the employees actively employed at our multi-site institution, only a proportion of all eligible participants enrolled; nonetheless, the sample size of the cohort was large, diverse, and representative of the source sample.[7] Our seroprevalence estimates were based on using a validated assay of only IgG antibodies; assays of IgM antibodies may offer complementary information in future studies. Data collected on medical history, exposures, and symptoms were all self-reported, similar to approaches used in prior studies. We were unable to completely verify prior COVID-19 illness using viral test results in part given lack of universally available testing for all individuals, particularly those with minimal to no symptoms. We observed that history of asthma was associated with lower odds of seropositivity, potentially related to use of corticosteroids or other immunosuppressive therapies; because information on these medications was not available in the current study, they warrant attention in future investigations. Although we collected information on work locations, data regarding specific professions and roles were not consistently captured. Further studies, including potentially training level and seniority of healthcare worker roles, are warranted. Additional details regarding the nature of clinical care provided in certain work areas, particularly those involving nasopharygeal or respiratory procedures, would also be important for future investigations.

In conclusion, in a highly diverse population of healthcare workers, demographic factors associated with COVID-19 antibody positivity indicate potential factors outside of the workplace associated with SARS-CoV-2 exposure, although these do not appear related to the number of

For peer review only - http://bmjopen.bmj.com/site/about/guidelines.xhtml

people or to the presence of children in the home. Further, while for dyspnea may be a marker of more severe disease among those with COVID-19, it's presence alone does not indicate infection.

DATA AVAILABILITY

The data that support the findings of this study are available from Cedars-Sinai Medical Center, upon reasonable request. The data are not publicly available due to the contents including information that could compromise research participant privacy/consent.

AUTHOR CONTRIBUTIONS

All authors contributed to and have approved the final manuscript. JEE and SC took part in conception, data collection, data analysis, drafting of the manuscript, and editing of the manuscript. GJB took part in data analysis, drafting of the manuscript, and editing of the manuscript. CMA took part in conception, data analysis, and editing of the manuscript. MAl., MAr., and JFB took part in editing of the manuscript. AHB, AB took part in data collection, data analysis, and editing of the manuscript. PB, WH, MH, and RVR took part in data collection and data analysis. JCF, SJ, EHK, PBM, TTN, MM MAR, and SSt. took part in data collection. JDG, SKH, MJ, YL, EL, DPBM, NM, and WGT took part in data analysis and editing of the manuscript. MK, DL, AM, KR, CER, SSh., and NS, took part in data analysis. KS took part in data collection, data analysis, drafting of the manuscript and editing of the manuscript. JEVE and JGB took part in conception, data analysis, drafting of the manuscript, and editing of the manuscript.

ACKNOWLEDGEMENTS

We are grateful to all the front-line healthcare workers in our healthcare system who continue to be dedicated to delivering the highest quality care for all patients.

FUNDING

This work was supported in part by Cedars Sinai Medical Center, the Erika J. Glazer Family Foundation, and NIH/NCI Grant U54-CA260591.

COMPETING INTERESTS

For peer review only - http://bmjopen.bmj.com/site/about/guidelines.xhtml

The authors declare that they have no competing interests.

For peer review only

For peer review only - http://bmjopen.bmj.com/site/about/guidelines.xhtml

REFERENCES

1. Bryant JE, Azman AS, Ferrari MJ, et al. Serology for SARS-CoV-2: Apprehensions, opportunities, and the path forward. *Science Immunology.* 2020;5(47):eabc6347.

2. Health CfDaR. Policy for Diagnostic Tests for Coronavirus Disease-2019 during the Public Health Emergency. In: Administraction FaD, ed: Dockets Management; 2020.

3. Nuccetelli M, Pieri M, Grelli S, et al. SARS-CoV-2 infection serology: a useful tool to overcome lockdown? *Cell Death Discov.* 2020;6:38.

4. Petherick A. Developing antibody tests for SARS-CoV-2. *Lancet.* 2020;395(10230):1101-1102.

5. Mallapaty S. Will antibody tests for the coronavirus really change everything? *Nature.* 2020;580(7805):571-572.

6. Espejo AP, Akgun Y, Al Mana AF, et al. Review of Current Advances in Serologic Testing for COVID-19. *Am J Clin Pathol.* 2020.

7. Ebinger JE, Botwin GJ, Albert CM, et al. An Opportune and Relevant Design for Studying the Health Trajectories of Healthcare Workers. *medRxiv.* 2020:2020.2006.2030.20140046.

8. Harris PA, Taylor R, Thielke R, Payne J, Gonzalez N, Conde JG. Research electronic data capture (REDCap)—A metadata-driven methodology and workflow process for providing translational research informatics support. *Journal of Biomedical Informatics.* 2009;42(2):377-381.

9. Harris PA, Taylor R, Minor BL, et al. The REDCap consortium: Building an international community of software platform partners. *J Biomed Inform.* 2019;95:103208.

10. Bryan A, Pepper G, Wener MH, et al. Performance Characteristics of the Abbott Architect SARS-CoV-2 IgG Assay and Seroprevalence in Boise, Idaho. *J Clin Microbiol.* 2020.

11. Menachemi N, Yiannoutsos CT, Dixon BE, et al. Population Point Prevalence of SARS-CoV-2 Infection Based on a Statewide Random Sample - Indiana, April 25-29, 2020. *MMWR Morb Mortal Wkly Rep.* 2020;69(29):960-964.

12. Barthélemy J, Suesse T. mipfp: An R Package for Multidimensional Array Fitting and Simulating Multivariate Bernoulli Distributions. *2018.* 2018;86(Code Snippet 2):20.

13. Dong Q, Gao X. Bayesian Estimation of the Seroprevalence of Antibodies to SARS-CoV-2. *JAMIA Open.* 2020.

14. Tilley K, Ayvazyan V, Martinez L, et al. A Cross-Sectional Study Examining the Seroprevalence of Severe Acute Respiratory Syndrome Coronavirus 2 Antibodies in a University Student Population. *J Adolesc Health.* 2020;67(6):763-768.

15. *RStan: the R interface to Stan. R package version 2.19.3* [computer program]. 2020.

For peer review only

For peer review only - http://bmjopen.bmj.com/site/about/guidelines.xhtml

16. Carpenter B, Gelman A, Hoffman MD, et al. Stan: A Probabilistic Programming Language. *2017.* 2017;76(1):32.

17. Mughal MS, Kaur IP, Patton CD, Mikhail NH, Vareechon C, Granet KM. The prevalence of severe acute respiratory coronavirus virus 2 (SARS-CoV-2) IgG antibodies in intensive care unit (ICU) healthcare personnel (HCP) and its implications-a single-center, prospective, pilot study. *Infect Control Hosp Epidemiol.* 2020:1-2.

18. Madsen T, Levin N, Niehus K, et al. Prevalence of IgG antibodies to SARS-CoV-2 among emergency department employees. *Am J Emerg Med.* 2020:S0735-6757(0720)30306-30305.

19. Moscola J, Sembajwe G, Jarrett M, et al. Prevalence of SARS-CoV-2 Antibodies in Health Care Personnel in the New York City Area. *JAMA.* 2020;324(9):893-895.

20. Stephens DS, McElrath MJ. COVID-19 and the Path to Immunity. *JAMA.* 2020;324(13):1279-1281.

21. Sood N, Simon P, Ebner P, et al. Seroprevalence of SARS-CoV-2-Specific Antibodies Among Adults in Los Angeles County, California, on April 10-11, 2020. *JAMA.* 2020;323(23):2425-2427.

22. Steensels D, Oris E, Coninx L, et al. Hospital-Wide SARS-CoV-2 Antibody Screening in 3056 Staff in a Tertiary Center in Belgium. *JAMA.* 2020.

23. Chowkwanyun M, Reed AL, Jr. Racial Health Disparities and Covid-19 - Caution and Context. *N Engl J Med.* 2020;383(3):201-203.

24. Rentsch CT, Kidwai-Khan F, Tate JP, et al. Covid-19 by Race and Ethnicity: A National Cohort Study of 6 Million United States Veterans. *medRxiv.* 2020.

25. Tai DBG, Shah A, Doubeni CA, Sia IG, Wieland ML. The Disproportionate Impact of COVID-19 on Racial and Ethnic Minorities in the United States. *Clin Infect Dis.* 2020.

26. Turner-Musa J, Ajayi O, Kemp L. Examining Social Determinants of Health, Stigma, and COVID-19 Disparities. *Healthcare (Basel).* 2020;8(2).

27. Thakur N, Lovinsky-Desir S, Bime C, et al. The Structural and Social Determinants of the Racial/Ethnic Disparities in the U.S. COVID-19 Pandemic: What's Our Role? *Am J Respir Crit Care Med.* 2020.

28. Raifman MA, Raifman JR. Disparities in the Population at Risk of Severe Illness From COVID-19 by Race/Ethnicity and Income. *Am J Prev Med.* 2020;59(1):137-139.

29. Lechien JR, Chiesa-Estomba CM, De Siati DR, et al. Olfactory and gustatory dysfunctions as a clinical presentation of mild-to-moderate forms of the coronavirus disease (COVID-19): a multicenter European study. *Eur Arch Otorhinolaryngol.* 2020;277(8):2251-2261.

30. Tong JY, Wong A, Zhu D, Fastenberg JH, Tham T. The Prevalence of Olfactory and Gustatory Dysfunction in COVID-19 Patients: A Systematic Review and Meta-analysis. *Otolaryngol Head Neck Surg.* 2020;163(1):3-11.

For peer review only - http://bmjopen.bmj.com/site/about/guidelines.xhtml

31. Lee DJ, Lockwood J, Das P, Wang R, Grinspun E, Lee JM. Self-reported anosmia and dysgeusia as key symptoms of coronavirus disease 2019. *CJEM.* 2020:1-8.

32. Zhu J, Zhong Z, Ji P, et al. Clinicopathological characteristics of 8697 patients with COVID-19 in China: a meta-analysis. *Fam Med Community Health.* 2020;8(2).

33. Kopel J, Perisetti A, Gajendran M, Boregowda U, Goyal H. Clinical Insights into the Gastrointestinal Manifestations of COVID-19. *Dig Dis Sci.* 2020;65(7):1932-1939.

34. DiazGranados CA, Dunning AJ, Kimmel M, et al. Efficacy of High-Dose versus Standard-Dose Influenza Vaccine in Older Adults. *New England Journal of Medicine.* 2014;371(7):635-645.

35. Long QX, Tang XJ, Shi QL, et al. Clinical and immunological assessment of asymptomatic SARS-CoV-2 infections. *Nat Med.* 2020.

36. Grifoni A, Weiskopf D, Ramirez SI, et al. Targets of T Cell Responses to SARS-CoV-2 Coronavirus in Humans with COVID-19 Disease and Unexposed Individuals. *Cell.* 2020;181(7):1489-1501 e1415.

37. Weiskopf D, Schmitz KS, Raadsen MP, et al. Phenotype and kinetics of SARS-CoV-2-specific T cells in COVID-19 patients with acute respiratory distress syndrome. *Sci Immunol.* 2020;5(48).

For peer review only

For peer review only - http://bmjopen.bmj.com/site/about/guidelines.xhtml

Table 1. Characteristics of the Study Sample

	Antibody Negative N=5850	Antibody Positive N=212
**Pre-Existing Characteristics**		
Age, mean (SD)	41.6 (12.0)	38.5 (11.2)
Male gender (%)	1876 (32)	73 (34)
Hispanic ethnicity (%)	1097 (19)	62 (29)
Race (%)		
Asian	1809 (31)	57 (27)
Black	354 (6)	18 (8)
White	2938 (50)	104 (49)
Other	749 (13)	33 (16)
Current smoker (%)	99 (2)	3 (1)
Current vape user (%)	83 (1)	4 (2)
Medical conditions (%)		
Asthma	733 (13)	14 (7)
Autoimmune disease	228 (4)	4 (2)
Cancer	195 (4)	3 (1)
Cardiovascular	127 (2)	2 (1)
Chronic Obstructive Pulmonary Disease	84 (2)	0 (0)
Diabetes Mellitus	371 (7)	8 (4)
Hypertension	967 (17)	26 (13)
BMI, mean (SD)	26.7 (5.6)	26.3 (5.1)
Obesity, BMI ≥ 30 (%)	998 (23)	32 (21)
**Potential COVID-19 Related Exposures**		
Personal diagnosis of COVID-19 (%)	530 (9)	104 (50)
Household member diagnosed with COVID-19 (%)	51 (1)	31 (15)
Domestic travel since September 2019 (%)	2127 (37)	54 (26)
International travel since September 2019 (%)	1324 (23)	44 (21)
Regular contact with COVID-19 patients (%)	1358 (24)	86 (41)
Work on a unit housing/caring for COVID-19 patients (%)	1600 (27)	93 (44)
Type of dwelling (%)		
Apartment	2636 (46)	93 (44)

For peer review only - http://bmjopen.bmj.com/site/about/guidelines.xhtml
For peer review only

House	2914 (51)	107 (51)
Other	216 (4)	9 (4)
No. people living in the home, mean (SD)	2.3 (1.7)	2.4 (1.8)
Any persons in the home under age 18 years (%)	1843 (32)	65 (31)
Any persons in the home under age 12 years (%)	1467 (25)	51 (24)
Cats as household pets (%)	783 (13)	27 (13)
Dogs as household pets (%)	2189 (37)	95 (45)
**Potential COVID-19 Related Responses**		
Fever (%)	497 (9)	87 (43)
Chills (%)	683 (12)	95 (46)
Headache (%)	2061 (36)	126 (61)
Conjunctivitis (%)	162 (3)	14 (7)
Anosmia (%)	252 (4)	107 (52)
Nasal congestion (%)	1611 (28)	104 (51)
Rhinorrhea (%)	1493 (26)	82 (41)
Dry cough (%)	1235 (22)	108 (53)
Productive cough (%)	542 (10)	50 (25)
Sore throat (%)	1368 (24)	81 (40)
Chest pain (%)	453 (8)	45 (22)
Dyspnea (%)	604 (11)	66 (33)
Anorexia (%)	390 (7)	78 (38)
Nausea (%)	657 (12)	52 (25)
Vomiting (%)	188 (3)	15 (8)
Diarrhea (%)	853 (15)	59 (29)
Myalgias (%)	1033 (18)	117 (58)
Fatigue (%)	1447 (25)	135 (66)
Skin changes (%)	261 (5)	15 (8)
Stroke symptoms (%)	35 (1)	3 (2)
Sneezing (%)	1863 (33)	94 (47)

For peer review only

FIGURE LEGEND

Figure 1. Seroprevalence Overall and by Subgroup

Figure 2. Pre-Existing Factors Associated with SARS-CoV-2 Seroprevalence

Figure 3. Potential COVID Illness Exposure Related Factors Associated with SARS-CoV-2

Figure 4. Potential COVID Illness Response Factors Associated with SARS-CoV-2 Seroprevalence

Figure 5. Factors Associated with SARS-CoV-2

For peer review only

For peer review only - http://bmjopen.bmj.com/site/about/guidelines.xhtml

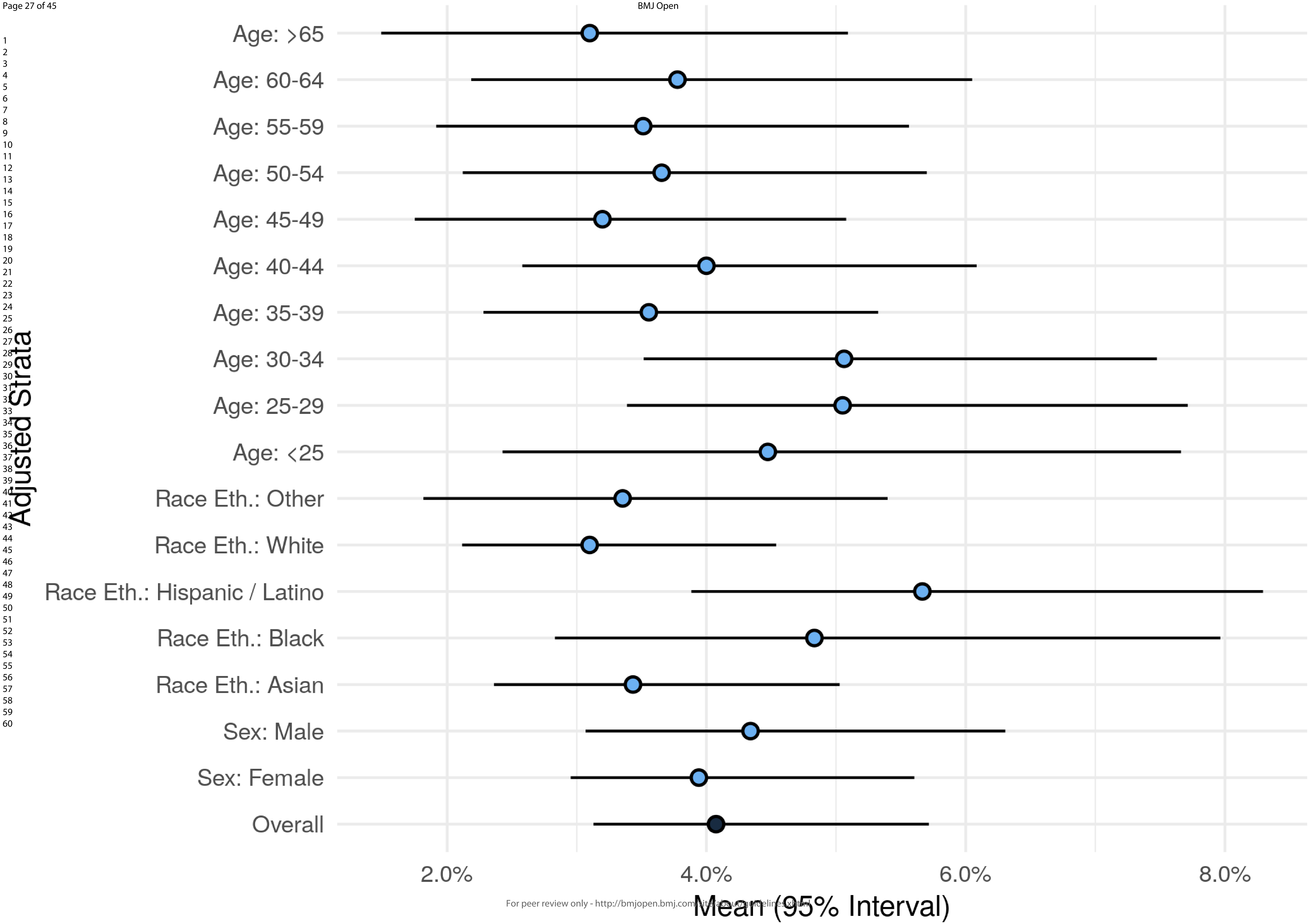
Age: >65
Age: 60-64
Age: 55-59
Age: 50-54
Age: 45-49
Age: 40-44
Age: 35-39
Age: 30-34
Age: 25-29
Age: <25
Race Eth.: Other
Race Eth.: White
Race Eth.: Hispanic / Latino
Race Eth.: Black
Race Eth.: Asian
Sex: Male
Sex: Female
Overall
Adjusted Strata
2.0%
4.0%
6.0%
8.0%
Mean (95% Interval)

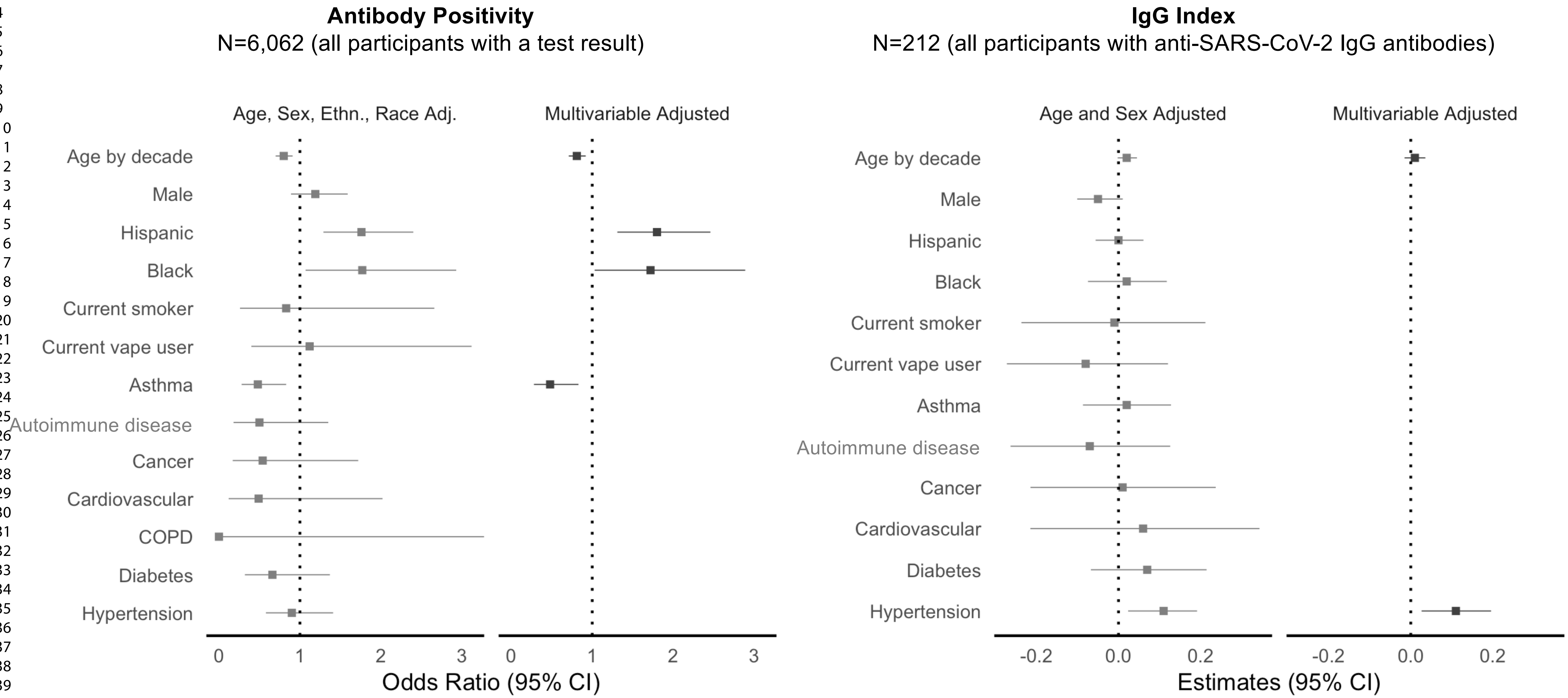
Antibody Positivity
N=6,062 (all participants with a test result)
Age, Sex, Ethn., Race Adj.
Multivariable Adjusted
Age by decade
Male
Hispanic
Black
Current smoker
Current vape user
Asthma
Autoimmune disease
Cancer
Cardiovascular
COPD
Diabetes
Hypertension
0
1
2
3
Odds Ratio (95% CI)
IgG Index
N=212 (all participants with anti-SARS-CoV-2 IgG antibodies)
Age and Sex Adjusted
Multivariable Adjusted
-0.2
0.0
0.2
Estimates (95% CI)

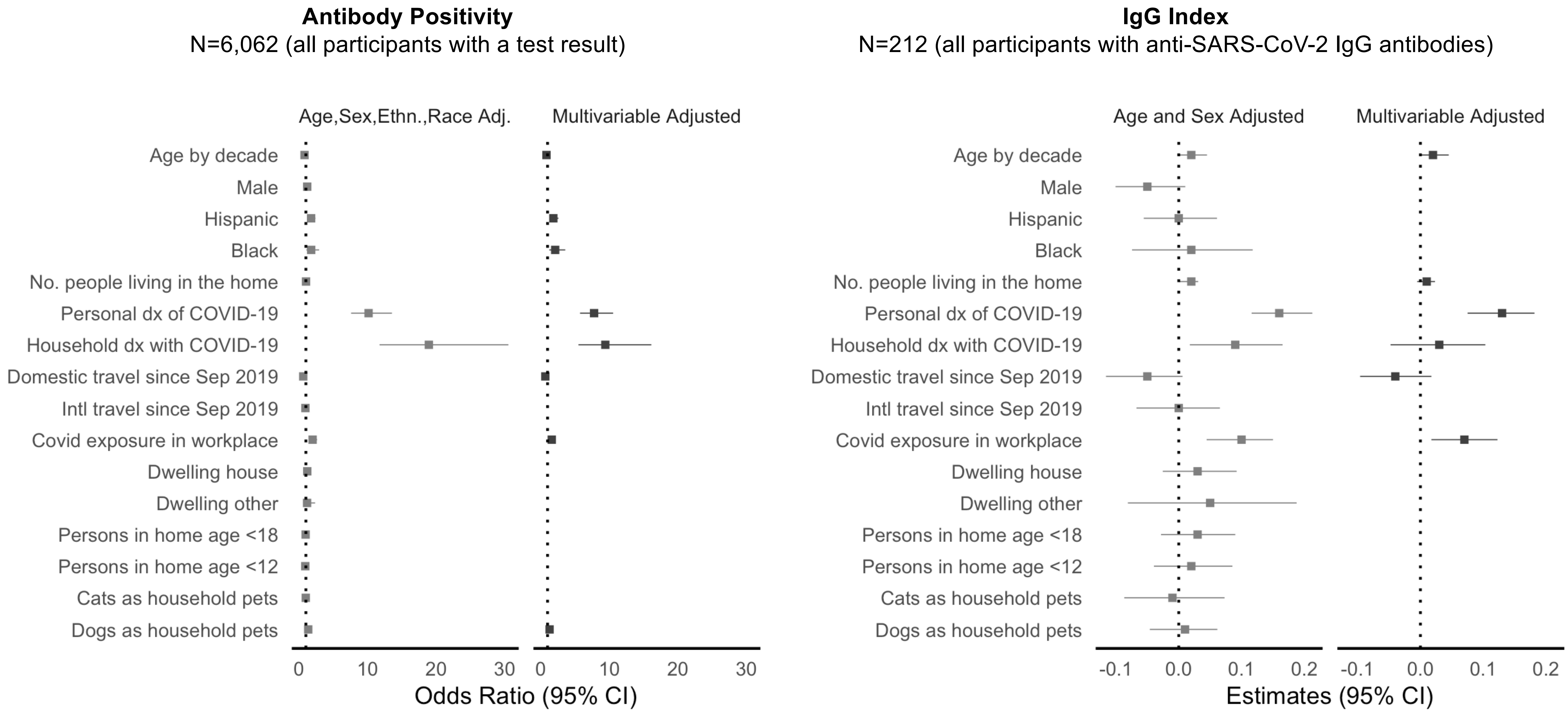
Antibody Positivity
N=6,062 (all participants with a test result)
Age,Sex,Ethn.,Race Adj.
Multivariable Adjusted
Age by decade
Male
Hispanic
Black
No. people living in the home
Personal dx of COVID-19
Household dx with COVID-19
Domestic travel since Sep 2019
Intl travel since Sep 2019
Covid exposure in workplace
Dwelling house
Dwelling other
Persons in home age <18
Persons in home age <12
Cats as household pets
Dogs as household pets
0 10 20 30
Odds Ratio (95% CI)
IgG Index
N=212 (all participants with anti-SARS-CoV-2 IgG antibodies)
Age and Sex Adjusted
Multivariable Adjusted
-0.1 0.0 0.1 0.2
Estimates (95% CI)

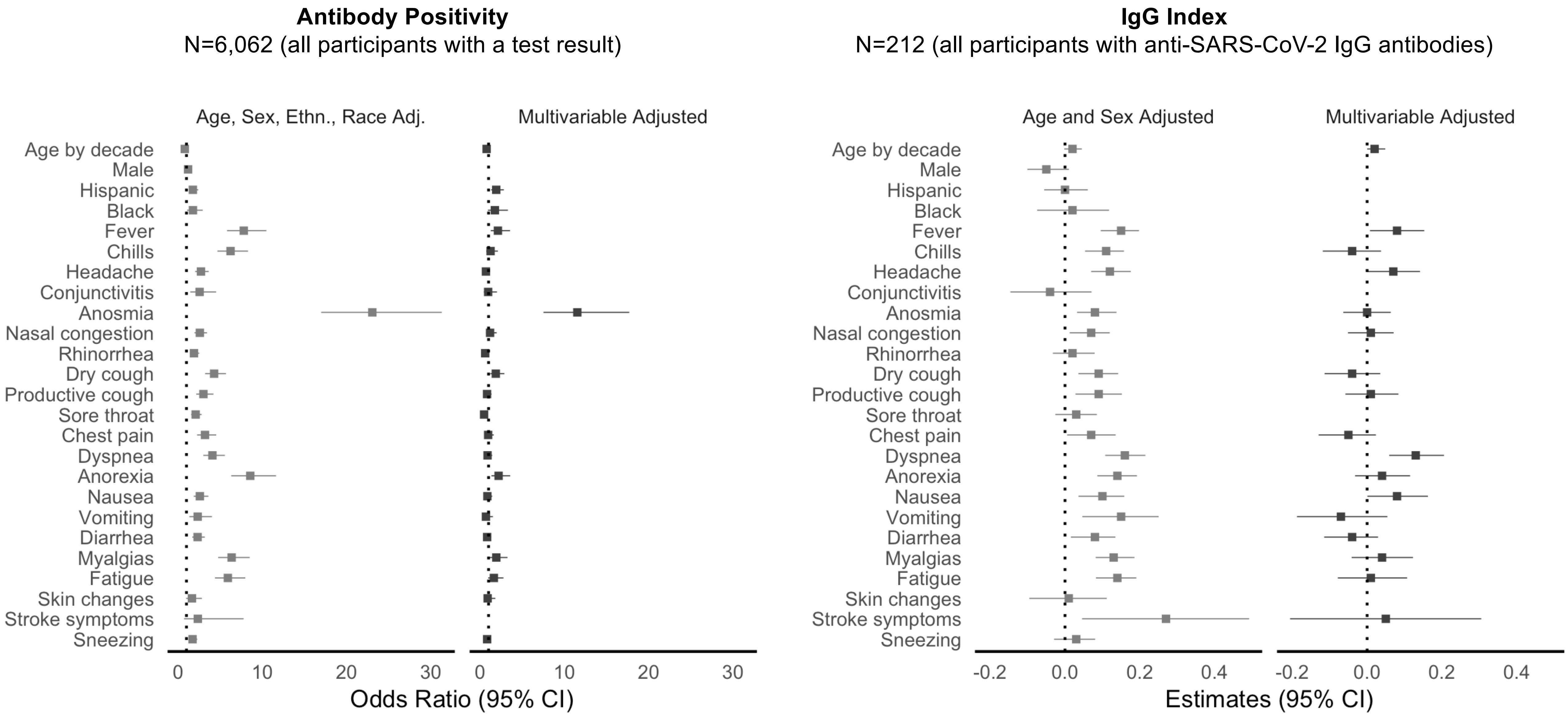
Antibody Positivity
N=6,062 (all participants with a test result)
Age, Sex, Ethn., Race Adj.
Multivariable Adjusted
Age by decade
Male
Hispanic
Black
Fever
Chills
Headache
Conjunctivitis
Anosmia
Nasal congestion
Rhinorrhea
Dry cough
Productive cough
Sore throat
Chest pain
Dyspnea
Anorexia
Nausea
Vomiting
Diarrhea
Myalgias
Fatigue
Skin changes
Stroke symptoms
Sneezing
0
10
20
30
Odds Ratio (95% CI)
IgG Index
N=212 (all participants with anti-SARS-CoV-2 IgG antibodies)
Age and Sex Adjusted
Multivariable Adjusted
-0.2
0.0
0.2
0.4
Estimates (95% CI)

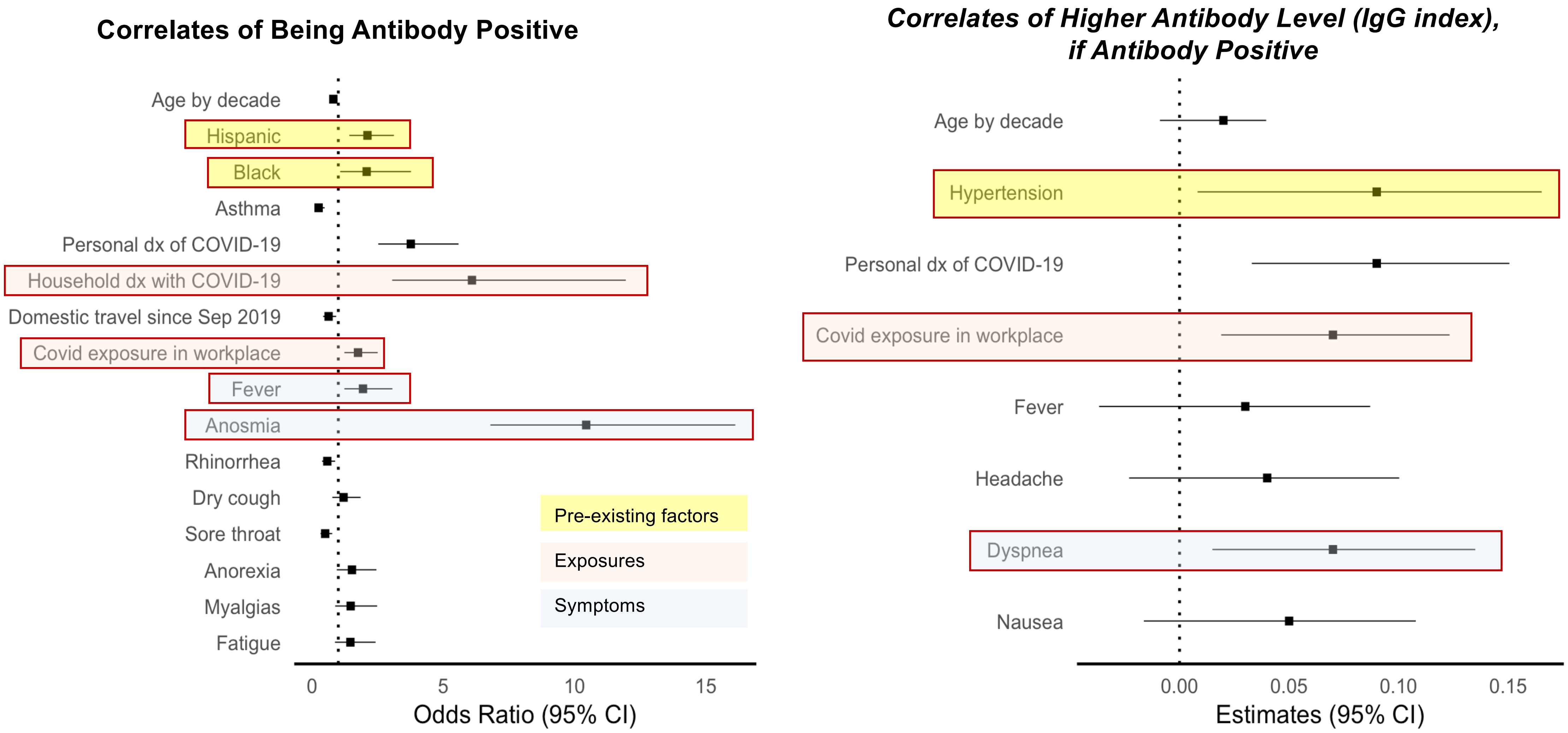
Correlates of Being Antibody Positive
Age by decade
Hispanic
Black
Asthma
Personal dx of COVID-19
Household dx with COVID-19
Domestic travel since Sep 2019
Covid exposure in workplace
Fever
Anosmia
Rhinorrhea
Dry cough
Sore throat
Anorexia
Myalgias
Fatigue
Pre-existing factors
Exposures
Symptoms
0
5
10
15
Odds Ratio (95% CI)
Correlates of Higher Antibody Level (IgG index), if Antibody Positive
Age by decade
Hypertension
Personal dx of COVID-19
Covid exposure in workplace
Fever
Headache
Dyspnea
Nausea
0.00
0.05
0.10
0.15
Estimates (95% CI)

SUPPLEMENTAL MATERIAL

Seroprevalence of Antibodies to SARS-CoV-2 in Healthcare Workers:

A Cross-Sectional Study

For peer review only

For peer review only - http://bmjopen.bmj.com/site/about/guidelines.xhtml

Supplemental Table 1. Prior Studies Reporting Sensitivity for the Abbott Architect SARS-CoV-2 IgG Assay[1-14]

Author	Positive Tests	Total Tests	Sensitivity %	Sample Description
Abbott[1]	109	115	94.78%	Using data from >=8 days post symptom onset and including 5 immunocompromised samples. Positive subjects who tested positive for SARS-CoV-2 by a polymerase chain reaction (PCR) method and who also presented with Covid-19 symptoms.
Bryan and Pepper et al.[2]	668	689	96.95%	Serum specimens sent for clinical testing from persons who tested RT-PCR positive for SARS-CoV -2 during March and April 2020.
Ng and Goldgof and Shy and Levine and Balcerek and Bapat et al.[15]	328	382	85.86%	Received care at adult inpatient units or clinics and were RT PCR positive for SARS-CoV-2 from nasopharyngeal and/or oropharyngeal swab testing. Using combined data from immunocompromised individuals. Combining data from Day 8 + PSO.
Ekelund et al.[4]	17	20	85.00%	Serum samples from 16 individuals that prior to serum sampling had tested RT-PCR positive for SARS-CoV-2 in nasopharyngeal and/or pharyngeal swabs. The interval between onset of Covid-19 symptoms to serum sample collection ranged from 18 to 52 days (median 38 days).
Phipps and SoRelle et al.[5]	10	21	47.62%	8 or more days PSO. suspected Covid-19 cases with PCR-based nasopharyngeal swab testing on the m2000 Abbott RealTime SARS Cov-2 assay or the Abbott ID NOWTM Covid-19 assay.
Phipps and SoRelle et al.[5]	10	13	76.92%	Indeterminate days from PSO. Suspected Covid-19 cases with PCR-based nasopharyngeal swab testing on the m2000 Abbott RealTime SARS Cov-2 assay or the Abbott ID NOWTM Covid-19 assay.
Chew et al.[6]	65	96	67.71%	Used COVID pts at different stage of disease: results based on 7 + PSO disease stage: ≤6 days (7/81), at 7–13 days (17/39), at 14–20 days (21/25), and at ≥21 days (27/32)
Theel et al.[7]	78	84	92.86%	Anti-SARS-CoV-2 IgG assay sensitivity in convalescent sera and in individual patients tested ≥15 days post-symptom onset or first positive SARS-CoV-2 RT-PCR result
Theel et al.[7]	123	175	70.29%	Included inpatients and outpatients PCR positive from >= 8 PSO
Kohmer et al.[8]	35	45	77.78%	From 45 pts with positive PCR
Stroemer et al.[9]	33	34	97.06%	34 sera obtained from 26 patients between four and 60 days (median 19 days) after a positive real-time RT-PCR.
Nicol et al.[10]	115	141	81.56%	141 serum from 82 patients with positive PCR varying days from PSO
Dellière et al.[11]	86	95	90.53%	Serum samples (n=95) from patients at least 10 days from symptoms onset or positive PCR
Perkmann et al. [12]	55	65	84.62%	65 Covid-19 donors/patients with a symptom onset to analysis time of ≥14 days
Mueller et al.[13]	7	8	87.50%	8 RT-PCR positive individuals
Tang et al.[14]	56	71	78.87%	103 specimens from 48 patients with PCR confirmed SARS-CoV-2 infections from NP, OP or lower respiratory swab. Reported positive results from time from PCR: 0d=12/27, 1-3d= 8/15, 3-7d=13/22, 8-13d=16/23, >14d=13/16. and reported positive from symptoms onset: <3d= 0/12, 3-7d=6/20, 8-13=11/23, >14d=45/48
Cedars-Sinai Department of Pathology and Laboratory Medicine*	53	60	88.33%	All COVID Positive subjects were selected by three criteria: (1) Presentation to Cedars-Sinai Medical Center with symptoms consistent with infection by SARS-CoV-2 virus; (2) Were PCR positive for SARS-CoV-2 viral RNA in at least one nasopharyngeal sample; (3) Had EDTA or heparin plasma available for testing which was collected 8 or more days after onset of symptoms according to physician's notes in the medical record.

For peer review only

*Unpublished data

For peer review only

For peer review only - http://bmjopen.bmj.com/site/about/guidelines.xhtml

Supplemental Table 2. Prior Studies Reporting Specificity for the Abbott Architect SARS-CoV-2 IgG Assay

Author	Negative Test	Total Tests	Specificity %	Sample source
Abbott[1]	1066	1070	99.63%	997 specimens were collected prior to September 2019<br>73 specimens were collected in 2020 with signs of respiratory illness and Covid-19 RT-PCR negative
Bryan and Pepper et al.[2]	1019	1020	99.90%	Serum samples from 2018 and 2019
Jääskeläinen et al.[16]	79	81	97.53%	Serum samples from 2018 and 2019
Ng, Goldgof, Shy, Levine, Balcerek and Bapat et al.[15]	1011	1013	99.80%	US blood donors prior to the Covid-19 pandemic
	234	235	99.57%	Plasma samples from 163 Covid-19 RT-PCR negative
Ekelund et al.[4]	100	100	100%	Pre-pandemic samples from 2018
Phipps and SoRelle et al.[5]	656	656	100%	240 samples collected prior to the Covid-19 pandemic (blood donors September through November 2019), and an additional 416 healthy donors without recent illness collected from March to April, 2020
	91	91	100%	23 CMV IgG positive, 8 prior Flu A+, 7 Flu B+, 6 RSV+, 47 endemic coronavirus samples (January 1, 2015- September 30, 2019) with normal or high levels of total IgG with no infusion of intravenous immunoglobulin in the preceding 3 months
	29	29	100%	Lupus patients that were positive for multiple autoantibodies (100% ANA, 62% anti-dsDNA, 75% anti-U1RNP, 55% anti-Sm, 34% anti-Ro52, 170 and 24% anti-La) 2004-2007
	20	20	100%	Rheumatoid arthritis patients positive for rheumatoid factor (85% were also anti-CCP positive) 2011-2014
	96	97	98.97%	Patients with Covid-19 RT-PCR negative
Chew et al.[6]	163	163	100%	
Theel et al.[7]	149	149	100%	Healthy samples from 2018
	104	105	99.05%	Samples negative for Covid-19 but positive for antibodies from other respiratory virus or bacteria (2020)
Kohmer et al.[8]	35	35	100%	
Ströemer et al.[9]	99	100	99.00%	100 archived samples from winter and summer seasons
Nicol et al.[10]	57	57	100%	52 patients with symptoms of Covid-19 but negative RT-PCR
	49	50	98.00%	Residual serum samples collected before Covid-19 in Mar 2019
	25	25	100%	Samples with potential cross-reaction to Covid-19
	10	10	100%	Samples from pregnant women
	10	10	100%	Samples with positive rheumatoid factor

For peer review only

For peer review only - http://bmjopen.bmj.com/site/about/guidelines.xhtml

Paiva et al.[17]	1055	1059	99.62%	Combining random Covid-19 samples during March 2020 (negative RT-PCR), pre-pandemic samples, and pre pandemic prenatal samples. False positive tests (4) were from samples with Hepatitis A, Hepatitis B, Rheumatoid Factor and anti-DNA
Brecher et al.[18]	20	20	100%	Patients with PCR Documented Common Cold
Dellière et al.[11]	42	42	100%	42 patients from pre-pandemic. 14 healthy, 16 endemic corona virus, 1 rhino virus, 1 metapneumovirus, 1 influenza A, 1 RSV. 1 HIV, 1 Hepatitis B. 1 toxoplasmosis. 2 Rheumatoid Factor
Perkmann et al.[12]	490	494	99.19%	Cross selection of Viennese population, LEAD study between November and April to enrich seasonal infections
	299	302	99.01%	Healthy voluntary donors
	356	358	99.44%	Patients with rheumatic disease
Mueller et al.[13]	26	26	100%	Patients with suspected Covid but negative neutralization test and PCR
Tang et al.[14]	152	153	99.35%	80 patients symptomatic for Covid-19 but negative RT-PCR. 50 samples collected in 2015. 5 samples with other corona virus infection. 4 samples with Influenza A or B. 14 samples with interfering antibiotics.
Cedars-Sinai Department of Pathology and Laboratory Medicine*	178	178	100%	Samples collected prior to 1/1/2020

For peer review only

Supplemental Table 3. Prevalence of Measurable SARS-CoV-2 IgG Antibody in the Study Sample

	**Mean (95% CI)**
**Overall**	**4.1 (3.1, 5.7)**
Sex: Female	3.9 (3.0, 5.6)
Sex: Male	4.3 (3.1, 6.3)
Age: <25	4.5 (2.4, 7.7)
Age: 25-29	5.1 (3.4, 7.7)
Age: 30-34	5.1 (3.5, 7.5)
Age: 35-39	3.6 (2.3, 5.3)
Age: 40-44	4 (2.6, 6.1)
Age: 45-49	3.2 (1.8, 5.1)
Age: 50-54	3.7 (2.1, 5.7)
Age: 55-59	3.5 (1.9, 5.6)
Age: 60-64	3.8 (2.2, 6.0)
Age: >65	3.1 (1.5, 5.1)
Race Eth.: Asian	3.4 (2.4, 5.0)
Race Eth.: Black	4.8 (2.8, 8.0)
Race Eth.: Hispanic / Latino	5.7 (3.9, 8.3)
Race Eth.: Other	3.4 (1.8, 5.4)
Race Eth.: White	3.1 (2.1, 4.5)

For peer review only

For peer review only - http://bmjopen.bmj.com/site/about/guidelines.xhtml

Supplemental Table 4. Pre-Existing Factors Associated with SARS-CoV-2 Seroprevalence

Predictors	Outcome: Antibody Positive N=6,062 (everybody with a test result)				Outcome: IgG index (divided by 10) N=212 (everybody with a test result)			
	Model 1		Model 2		Model 3		Model 4	
	OR (95% CI)	P	OR (95% CI)	P	Est (SE)	P	Est (SE)	P
Age (per decade)	0.80 (0.70, 0.91)	0.001	0.81 (0.71, 0.92)	0.001	0.02 (0.01)	0.07		
Male Sex	1.19 (0.89, 1.59)	0.24			-0.05 (0.03)	0.11		
Hispanic Ethnicity	1.76 (1.29, 2.40)	<0.001	1.80 (1.31, 2.46)	<0.001	0.00 (0.03)	0.93		
African American Race	1.77 (1.07, 2.93)	0.027	1.72 (1.03, 2.89)	0.04	0.02 (0.05)	0.66		
Smoking	0.83 (0.26, 2.66)	0.76			-0.01 (0.11)	0.91		
Vaping	1.12 (0.40, 3.12)	0.82			-0.08 (0.10)	0.45		
Asthma	0.48 (0.28, 0.83)	0.009	0.48 (0.28, 0.83)	0.009	0.02 (0.05)	0.71		
Autoimmune disease	0.50 (0.18, 1.35)	0.17			-0.07 (0.10)	0.49		
Cancer	0.54 (0.17, 1.72)	0.29			0.01 (0.12)	0.92		
Cardiovascular Disease	0.49 (0.12, 2.02)	0.33			0.06 (0.14)	0.65		
Chronic Obstructive Pulmonary Disease	0.00 (0.00, inf)	0.97						
Diabetes Mellitus	0.66 (0.32, 1.37)	0.26			0.07 (0.07)	0.31		
Hypertension	0.90 (0.58, 1.41)	0.64			0.11 (0.04)	0.013	0.12 (0.04)	0.003
Obesity	0.82 (0.55, 1.24)	0.35			0.01 (0.04)	0.71		

Logistic model 1 is adjusted for age, sex, ethnicity, race.
Logistic model 2 is adjusted for anything that was significant in Model 1 to a P<0.05.

Linear model 3 is adjusted for age, sex
Linear model 4 is adjusted for anything that was significant in Model 3 to a P<0.05.

For peer review only - http://bmjopen.bmj.com/site/about/guidelines.xhtml

Supplemental Table 5. Potential COVID Illness Exposure Related Factors Associated with SARS-CoV-2 Seroprevalence

	Outcome: Antibody Positive N=6,062 (everybody with a test result)				Outcome: IgG index (divided by 10) N=212 (everybody with a test result)			
	Model 1		Model 2		Model 3		Model 4	
Predictors	OR (95% CI)	P	OR (95% CI)	P	Est (SE)	P	Est (SE)	P
Age (per decade)	0.80 (0.70, 0.91)	0.001	0.84 (0.73, 0.97)	0.016	0.02 (0.01)	0.07		
Male Sex	1.19 (0.89, 1.59)	0.24			-0.05 (0.03)	0.11		
Hispanic Ethnicity	1.76 (1.28, 2.4)	<0.001	1.84 (1.31, 2.59)	0.001	0.00 (0.03)	0.93		
African American Race	1.77 (1.07, 2.93)	0.027	2.11 (1.24, 3.58)	0.006	0.02 (0.05)	0.66		
# people in home	1.02 (0.94, 1.11)	0.6			0.02 (0.01)	0.038	0.01 (0.01)	0.13
Physician Suspected Covid Diagnosis	10.14 (7.59, 13.55)	<0.001	7.78 (5.73, 10.56)	<0.001	0.16 (0.02)	<0.001	0.13 (0.03)	<0.001
Household Covid Diagnosis	18.93 (11.74, 30.53)	<0.001	9.42 (5.5, 16.13)	<0.001	0.09 (0.04)	0.016	0.02 (0.04)	0.55
Domestic Travel	0.61 (0.44, 0.84)	0.002	0.67 (0.48, 0.94)	0.021	-0.05 (0.03)	0.08		
International Travel	0.93 (0.66, 1.31)	0.68			0.00 (0.03)	0.98		
Covid Unit	1.98 (1.49, 2.63)	<0.001	1.61 (1.18, 2.18)	0.002	0.10 (0.03)	<0.001	0.06 (0.03)	0.026
Dwelling: House	1.20 (0.89, 1.61)	0.23			0.03 (0.03)	0.27		
Dwelling: Other	1.17 (0.58, 2.35)	0.67			0.05 (0.07)	0.44		
Persons <18 in home	0.96 (0.71, 1.29)	0.77			0.03 (0.03)	0.31		
Person <12 in home	0.91 (0.66, 1.26)	0.58			0.02 (0.03)	0.47		
Cats in home	0.98 (0.65, 1.48)	0.92			-0.01 (0.04)	0.87		
Dogs in home	1.34 (1.02, 1.78)	0.039	1.29 (0.95, 1.75)	0.10	0.01 (0.03)	0.78		

Logistic model 1 is adjusted for age, sex, race, ethnicity.
Logistic model 2 is adjusted for anything that was significant in Model 1 to a P<0.05.

Linear model 3 is adjusted age, sex
Linear model 4 is adjusted for anything that was significant in Model 3 to a P<0.05.

For peer review only - http://bmjopen.bmj.com/site/about/guidelines.xhtml

Supplemental Table 6. Potential COVID Illness Response Factors Associated with SARS-CoV-2 Seroprevalence

	Outcome: Antibody Positive N=6,062 (everybody with a test result)				Outcome: IgG index (divided by 10) N=212 (everybody with a test result)			
	Model 1		Model 2		Model 3		Model 4	
Predictors	OR (95% CI)	P	OR (95% CI)	P	Est (SE)	P	Est (SE)	P
Age (per decade)	0.8 (0.7, 0.91)	0.001	0.77 (0.66, 0.91)	0.002	0.02 (0.01)	0.07		
Male Sex	1.19 (0.89, 1.59)	0.24			-0.05 (0.03)	0.11		
Hispanic Ethnicity	1.76 (1.29, 2.4)	<0.001	1.93 (1.31, 2.84)	0.001	0 (0.03)	0.93		
African American Race	1.77 (1.07, 2.93)	0.027	1.72 (0.91, 3.26)	0.09	0.02 (0.05)	0.66		
Fever	7.8 (5.81, 10.48)	<0.001	2.2 (1.31, 3.69)	0.003	0.15 (0.03)	<0.001	0.08 (0.04)	0.032
Chills	6.23 (4.67, 8.31)	<0.001	1.28 (0.75, 2.18)	0.36	0.11 (0.03)	<0.001	-0.04 (0.04)	0.31
Headache	2.72 (2.03, 3.64)	<0.001	0.67 (0.43, 1.06)	0.09	0.12 (0.03)	<0.001	0.06 (0.04)	0.11
Conjunctivitis	2.56 (1.45, 4.52)	0.001	0.89 (0.42, 1.86)	0.75	-0.04 (0.06)	0.5		
Anosmia	23.05 (16.98, 31.29)	<0.001	11.91 (7.77, 18.24)	<0.001	0.08 (0.03)	0.002	-0.01 (0.03)	0.81
Nasal Congestion	2.59 (1.95, 3.44)	<0.001	1.22 (0.73, 2.04)	0.44	0.07 (0.03)	0.017	0.01 (0.03)	0.83
Rhinorrhea	1.89 (1.41, 2.52)	<0.001	0.59 (0.36, 0.97)	0.039	0.02 (0.03)	0.41		
Dry Cough	4.28 (3.21, 5.69)	<0.001	1.82 (1.18, 2.81)	0.007	0.09 (0.03)	0.001	-0.04 (0.04)	0.25
Productive Cough	3.01 (2.16, 4.2)	<0.001	0.83 (0.5, 1.37)	0.46	0.09 (0.03)	0.005	0.01 (0.04)	0.73
Sore Throat	2.09 (1.56, 2.8)	<0.001	0.48 (0.31, 0.75)	0.001	0.03 (0.03)	0.3		
Chest Pain	3.2 (2.26, 4.53)	<0.001	0.96 (0.56, 1.63)	0.88	0.07 (0.03)	0.034	-0.05 (0.04)	0.24
Dyspnea	4.08 (3, 5.56)	<0.001	0.88 (0.54, 1.43)	0.6	0.16 (0.03)	<0.001	0.13 (0.04)	0.001
Anorexia	8.57 (6.31, 11.63)	<0.001	2.19 (1.34, 3.57)	0.002	0.14 (0.03)	<0.001	0.06 (0.04)	0.13
Nausea	2.59 (1.86, 3.6)	<0.001	0.88 (0.52, 1.47)	0.62	0.1 (0.03)	0.002	0.08 (0.04)	0.049
Vomiting	2.33 (1.34, 4.03)	0.003	0.67 (0.3, 1.47)	0.31	0.15 (0.05)	0.005	-0.06 (0.06)	0.35
Diarrhea	2.32 (1.69, 3.18)	<0.001	0.82 (0.52, 1.29)	0.39	0.08 (0.03)	0.014	-0.05 (0.04)	0.22
Myalgias	6.36 (4.76, 8.5)	<0.001	1.88 (1.11, 3.17)	0.019	0.13 (0.03)	<0.001	0.04 (0.04)	0.35
Fatigue	5.91 (4.38, 7.98)	<0.001	1.58 (0.93, 2.69)	0.09	0.14 (0.03)	<0.001	0.02 (0.05)	0.67
Skin Changes	1.65 (0.96, 2.83)	0.07			0.01 (0.05)	0.88		
Stroke Symptoms	2.35 (0.71, 7.78)	0.16			0.27 (0.11)	0.019	0.05 (0.13)	0.73
Sneezing	1.72 (1.29, 2.28)	<0.001	0.82 (0.52, 1.31)	0.41	0.03 (0.03)	0.36		

Logistic model 1 is adjusted for age, sex, race, ethnicity.
Logistic model 2 is adjusted for anything that was significant in Model 1 to a P<0.05.

Linear model 3 is adjusted for age, sex.
Linear model 4 is adjusted for anything that was significant in Model 3 to a P<0.05.

For peer review only

Supplemental Table 7. Factors Associated with SARS-CoV-2

Predictors	Outcome: Antibody Positive N=6,062 (everybody with a test result)		Outcome: IgG index (divided by 10) N=212 (everybody with a test result)	
	OR (95% CI)	P	Est (SE)	P
Age (per decade)	0.80 (0.68, 0.94)	0.008		
Hispanic Ethnicity	1.98 (1.34, 2.92)	0.001		
African American Race	2.02 (1.08, 3.76)	0.027		
Asthma	0.25 (0.13, 0.51)	<0.001		
Hypertension			0.1 (0.04)	0.007
Physician Suspected Covid Diagnosis	3.85 (2.6, 5.69)	<0.001	0.1 (0.03)	0.001
Household Covid Diagnosis	5.73 (2.9, 11.32)	<0.001		
Domestic Travel	0.62 (0.42, 0.91)	0.015		
Covid Unit	1.76 (1.24, 2.5)	0.002	0.06 (0.03)	0.021
Fever	2.02 (1.28, 3.18)	0.002	0.03 (0.03)	0.26
Anosmia	11.04 (7.22, 16.88)	<0.001		
Rhinorrhea	0.58 (0.38, 0.88)	0.011		
Dry Cough	1.3 (0.84, 2)	0.23		
Sore Throat	0.53 (0.34, 0.82)	0.004		
Dyspnea			0.08 (0.03)	0.009
Anorexia	1.58 (0.98, 2.54)	0.06		
Nausea			0.06 (0.03)	0.05
Myalgias	1.65 (1.04, 2.63)	0.035		

Logistic and linear models are adjusted for significant predictors from the primary multivariable models examining associations of existing characteristics, exposures and symptoms with antibody positivity and IgG index.

For peer review only - http://bmjopen.bmj.com/site/about/guidelines.xhtml

Supplemental Figure 1.

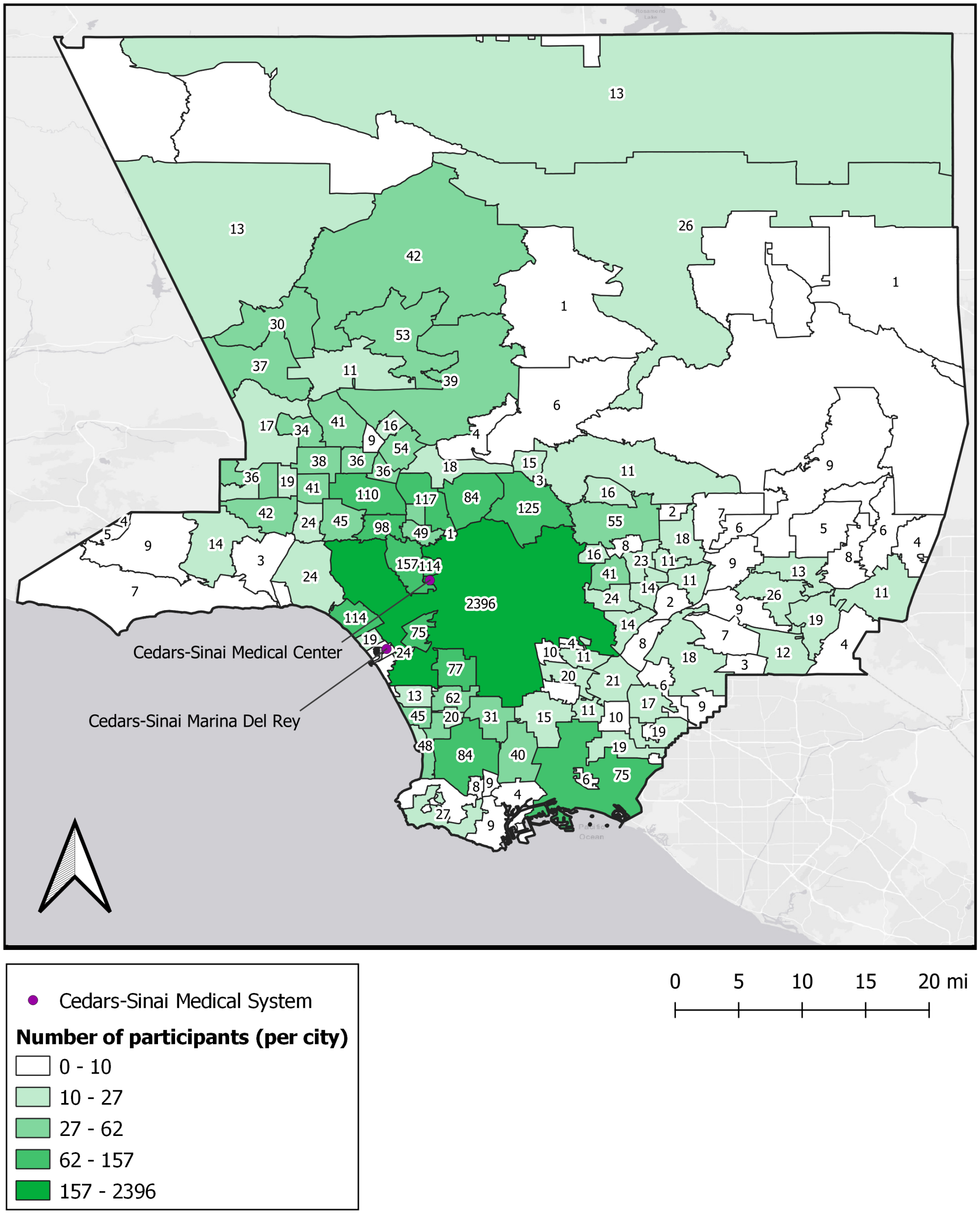

SUPPLEMENTAL REFERENCES

1. Abbott. ARCHITECT SARS-CoV-2 IgG Instructions for Use. 2020.
2. Bryan A, Pepper G, Wener MH, et al. Performance Characteristics of the Abbott Architect SARS-CoV-2 IgG Assay and Seroprevalence in Boise, Idaho. *J Clin Microbiol.* 2020.
3. Ng D, Goldgof G, Shy B, et al. SARS-CoV-2 seroprevalence and neutralizing activity in donor and patient blood from the San Francisco Bay Area. *medRxiv.* 2020:2020.2005.2019.20107482.
4. Ekelund O, Ekblom K, Somajo S, Pattison-Granberg J, Olsson K, Petersson A. High-throughput immunoassays for SARS-CoV-2, considerable differences in performance when comparing three methods. *medRxiv.* 2020:2020.2005.2022.20106294.
5. Phipps WS, SoRelle JA, Li Q-Z, et al. SARS-CoV-2 Antibody responses do not predict COVID-19 disease severity. *medRxiv.* 2020:2020.2005.2015.20103580.
6. Chew KL, Tan SS, Saw S, et al. Clinical evaluation of serological IgG antibody response on the Abbott Architect for established SARS-CoV-2 infection. *Clinical Microbiology and Infection.* 2020.
7. Theel ES, Harring J, Hilgart H, Granger D. Performance Characteristics of Four High-Throughput Immunoassays for Detection of IgG Antibodies against SARS-CoV-2. *Journal of Clinical Microbiology.* 2020:JCM.01243-01220.
8. Kohmer N, Westhaus S, Rühl C, Ciesek S, Rabenau HF. Brief clinical evaluation of six high-throughput SARS-CoV-2 IgG antibody assays. *Journal of Clinical Virology.* 2020;129:104480.
9. Stroemer A, Grobe O, Rose R, Fickenscher H, Lorentz T, Krumbholz A. Diagnostic accuracy of six commercial SARS-CoV-2 IgG/total antibody assays and identification of SARS-CoV-2 neutralizing antibodies in convalescent sera. *medRxiv.* 2020:2020.2006.2015.20131672.

For peer review only - http://bmjopen.bmj.com/site/about/guidelines.xhtml

10. Nicol T, Lefeuvre C, Serri O, et al. Assessment of SARS-CoV-2 serological tests for the diagnosis of COVID-19 through the evaluation of three immunoassays: Two automated immunoassays (Euroimmun and Abbott) and one rapid lateral flow immunoassay (NG Biotech). *Journal of Clinical Virology.* 2020;129:104511.

11. Dellière S, Salmona M, Minier M, et al. Evaluation of COVID-19 IgG/IgM Rapid Test from Orient Gene Biotech. *Journal of Clinical Microbiology.* 2020:JCM.01233-01220.

12. Perkmann T, Perkmann-Nagele N, Breyer M-K, et al. Side by side comparison of three fully automated SARS-CoV-2 antibody assays with a focus on specificity. *medRxiv.* 2020:2020.2006.2004.20117911.

13. Mueller L, Ostermann PN, Walker A, et al. Sensitivity of commercial Anti-SARS-CoV-2 serological assays in a high-prevalence setting. *medRxiv.* 2020:2020.2006.2011.20128686.

14. Tang MS, Hock KG, Logsdon NM, et al. Clinical Performance of Two SARS-CoV-2 Serologic Assays. *Clinical Chemistry.* 2020.

15. Guo W, Li M, Dong Y, et al. Diabetes is a risk factor for the progression and prognosis of COVID-19. *Diabetes/metabolism research and reviews.* 2020:e3319.

16. Jääskeläinen AJ, Kuivanen S, Kekäläinen E, et al. Performance of six SARS-CoV-2 immunoassays in comparison with microneutralisation. *medRxiv.* 2020:2020.2005.2018.20101618.

17. Paiva KJ, Grisson RD, Chan PA, et al. Validation and Performance Comparison of Three SARS-CoV-2 Antibody Assays. *bioRxiv.* 2020:2020.2005.2029.124776.

18. Brecher SM, Dryjowicz-Burek J, Yu H, Campbell S, Ratcliffe N, Gupta K. Patients with Common Cold Coronaviruses Tested Negative for IgG Antibody to SARS-CoV-2. *Journal of Clinical Microbiology.* 2020:JCM.01029-01020.

For peer review only

For peer review only - http://bmjopen.bmj.com/site/about/guidelines.xhtml

STROBE 2007 (v4) Statement—Checklist of items that should be included in reports of *cross-sectional studies*

Section/Topic	Item #	Recommendation	Reported on page #
**Title and abstract**	1	(*a*) Indicate the study's design with a commonly used term in the title or the abstract	Pg.3
		(*b*) Provide in the abstract an informative and balanced summary of what was done and what was found	Pg. 3-4
**Introduction**			
Background/rationale	2	Explain the scientific background and rationale for the investigation being reported	Pg.5
Objectives	3	State specific objectives, including any prespecified hypotheses	Pg.5
**Methods**			
Study design	4	Present key elements of study design early in the paper	Pg.6
Setting	5	Describe the setting, locations, and relevant dates, including periods of recruitment, exposure, follow-up, and data collection	Pg.6
Participants	6	(*a*) Give the eligibility criteria, and the sources and methods of selection of participants	Pg.6
Variables	7	Clearly define all outcomes, exposures, predictors, potential confounders, and effect modifiers. Give diagnostic criteria, if applicable	Pg.7-8
Data sources/ measurement	8*	For each variable of interest, give sources of data and details of methods of assessment (measurement). Describe comparability of assessment methods if there is more than one group	Pg.6-8
Bias	9	Describe any efforts to address potential sources of bias	Pg.6-7
Study size	10	Explain how the study size was arrived at	Pg.6
Quantitative variables	11	Explain how quantitative variables were handled in the analyses. If applicable, describe which groupings were chosen and why	Pg.7
Statistical methods	12	(*a*) Describe all statistical methods, including those used to control for confounding	Pg.7-8
		(*b*) Describe any methods used to examine subgroups and interactions	Pg.7-8
		(*c*) Explain how missing data were addressed	Pg.7-8
		(*d*) If applicable, describe analytical methods taking account of sampling strategy	Pg.7-8
		(*e*) Describe any sensitivity analyses	Pg.7-8
**Results**			

For peer review only

Participants	13*	(a) Report numbers of individuals at each stage of study—eg numbers potentially eligible, examined for eligibility, confirmed eligible, included in the study, completing follow-up, and analysed	Pg.9
		(b) Give reasons for non-participation at each stage	Pg.6
		(c) Consider use of a flow diagram	N/A
Descriptive data	14*	(a) Give characteristics of study participants (eg demographic, clinical, social) and information on exposures and potential confounders	Pg. 9
		(b) Indicate number of participants with missing data for each variable of interest	N/A
Outcome data	15*	Report numbers of outcome events or summary measures	Pg. 9-10
Main results	16	(*a*) Give unadjusted estimates and, if applicable, confounder-adjusted estimates and their precision (eg, 95% confidence interval). Make clear which confounders were adjusted for and why they were included	Pg.9-10
		(*b*) Report category boundaries when continuous variables were categorized	Pg.9-10
		(*c*) If relevant, consider translating estimates of relative risk into absolute risk for a meaningful time period	NA
Other analyses	17	Report other analyses done—eg analyses of subgroups and interactions, and sensitivity analyses	Pg.7-8
**Discussion**			
Key results	18	Summarise key results with reference to study objectives	Pg. 11-14
Limitations	19	Discuss limitations of the study, taking into account sources of potential bias or imprecision. Discuss both direction and magnitude of any potential bias	Pg. 14
Interpretation	20	Give a cautious overall interpretation of results considering objectives, limitations, multiplicity of analyses, results from similar studies, and other relevant evidence	Pg. 14
Generalisability	21	Discuss the generalisability (external validity) of the study results	Pg. 13-14
**Other information**			
Funding	22	Give the source of funding and the role of the funders for the present study and, if applicable, for the original study on which the present article is based	Pg. 15

*Give information separately for cases and controls in case-control studies and, if applicable, for exposed and unexposed groups in cohort and cross-sectional studies.

Note: An Explanation and Elaboration article discusses each checklist item and gives methodological background and published examples of transparent reporting. The STROBE checklist is best used in conjunction with this article (freely available on the Web sites of PLoS Medicine at http://www.plosmedicine.org/, Annals of Internal Medicine at http://www.annals.org/, and Epidemiology at http://www.epidem.com/). Information on the STROBE Initiative is available at www.strobe-statement.org.

For peer review only
